# Unsupervised assessment of microarray data quality using a Gaussian mixture model

**DOI:** 10.1186/1471-2105-10-191

**Published:** 2009-06-22

**Authors:** Brian E Howard, Beate Sick, Steffen Heber

**Affiliations:** 1 Bioinformatics Research Center, North Carolina State University, Raleigh, NC, USA; 2 Institute of Data Analysis and Process Design, Zurich University of Applied Science, Winterthur, Switzerland

## Abstract

**Background:**

Quality assessment of microarray data is an important and often challenging aspect of gene expression analysis. This task frequently involves the examination of a variety of summary statistics and diagnostic plots. The interpretation of these diagnostics is often subjective, and generally requires careful expert scrutiny.

**Results:**

We show how an unsupervised classification technique based on the Expectation-Maximization (EM) algorithm and the naïve Bayes model can be used to automate microarray quality assessment. The method is flexible and can be easily adapted to accommodate alternate quality statistics and platforms. We evaluate our approach using Affymetrix 3' gene expression and exon arrays and compare the performance of this method to a similar supervised approach.

**Conclusion:**

This research illustrates the efficacy of an unsupervised classification approach for the purpose of automated microarray data quality assessment. Since our approach requires only unannotated training data, it is easy to customize and to keep up-to-date as technology evolves. In contrast to other "black box" classification systems, this method also allows for intuitive explanations.

## Background

Recently, the MicroArray Quality Control (MAQC) consortium found that most microarray platforms will generate reproducible data when used correctly by experienced researchers [1]. Despite this positive result, it has been suggested that 20% or more of the data available in public microarray data repositories may be of questionable quality [2]. For this reason, discriminating between high and low quality microarray data is of the highest importance, and several recent publications have dealt with this problem; detailed reviews are provided by Wilkes *et al. *[3] and Eads *et al. *[4].

Several approaches have emphasized the importance of measuring, either directly or indirectly, the integrity of the RNA samples used in the experiment (e.g. [5-7]). Other research has focused on spatial artifacts: problems that typically arise during hybridization due to bubbling, scratches and edge effects [8,9].

In the case of Affymetrix GeneChips, which we will use to demonstrate our method, there are standard benchmark tests provided by the manufacturer [10]. A standard complementary approach is to use the R statistical software, along with the BioConductor [11] "affy" [12] and "affyPLM" [13] packages, to produce a series of diagnostic plots for the assessment of GeneChip quality (see additional file 1: Fig S3, S4). A review of the quality control features available in BioConductor can be found in [14], and a variety of software packages are now available to assist in the automation of this process [15-19].

In general, the goal of these approaches is to identify chips that are outliers – either in relation to other chips in the same experiment or the entire theoretical population of similar chips. Often, it is assumed that a rational decision regarding data quality is made only after considering several quasi-orthogonal dimensions of quality. Chips are typically rejected only after a preponderance of the evidence indicates poor quality; a slightly unusual score on a single metric is frequently ignored, while a number of moderately or highly unusual scores on a variety of quality metrics is often grounds for exclusion of a particular chip from further analysis. However, there are no universal, robust thresholds available for the identification of outliers according to the various quality variables. Instead, decisions are necessarily made using historical data, either implicitly or explicitly.

Therefore, recent efforts have focused on providing a "holistic", accurate, and automatic interpretation of diagnostic plots and quality metrics. Burgoon *et al. *[20] describe a custom, in-house protocol for assessing data quality of two-color spotted cDNA arrays. The authors advocate an integrated "Quality Assurance Plan" which attempts to integrate quality control at every level of the experimental procedure.

Another example is the RACE system [15,16]. This system utilizes various statistics extracted from the BioConductor diagnostic plots, along with a random forest classifier, to automatically identify low quality data. However, like the quality assurance protocol described by Burgoon *et al.*, the RACE system relies on a large expert-annotated data set. For this reason, it is difficult to keep the system up-to-date in the face of rapidly changing technology, with new chip types continually being introduced into the market. A further challenge is to adapt such a system to similar, but slightly different, types of data such as Affymetrix SNP arrays, exon arrays, or arrays produced by other manufacturers such as Illumina and Agilent.

In this paper we investigate a method for unsupervised classification that was designed with these considerations in mind. First, we describe how to frame the interpretation of microarray quality indicators as an unsupervised classification problem using a Gaussian mixture model. We show how the model parameters can be estimated using the Expectation-Maximization (EM) algorithm [21], and how they can be used to construct a Naïve Bayes classifier for identifying low quality data.

Previous work has demonstrated that naïve Bayes classifiers perform well with labeled training sets in the supervised version of the problem discussed in this paper [15]. The combination of Naïve Bayes together with EM has been used with considerable success in other problem domains, including text classification [22]. Gaussian mixture models have been applied to automatic quality assessment of phone signal clarity [23] and mass spectrometry data [24], and in other stages of the microarray processing pipe-line, including identification of differentially expressed genes [25], assessment of the concordance between sets of similar microarray data sets [26], and even quality control at the spot detection and image fluorescence analysis level [27]. However, this is the first research we are aware of that employs this estimation approach, in conjunction with a naïve Bayes classifier, for the purpose of array-level quality control of microarray data.

In the following sections, we describe the datasets used in this research, and explain the implementation of both the supervised and unsupervised versions of the quality classifier. We demonstrate that the performance of the unsupervised classifier is comparable to a supervised classifier constructed from expert-labeled data. We also apply the algorithm to Affymetrix exon array data, and compare the observed quality indicator distributions with those obtained from 3' expression arrays.

## Methods

### Datasets

Our first dataset is a set of 603 Affymetrix raw intensity microarray data files, from 32 distinct experiments downloaded from the NCBI GEO database [28]. A variety of Affymetrix GeneChip 3' Expression array types are represented in the dataset, including: ath1121501 (Arabidopsis, 248 chips; GEO accession numbers: GSE5770, GSE5759, GSE911 [29], GSE2538 [30], GSE3350 [31], GSE3416 [32], GSE5534, GSE5535, GSE5530, GSE5529, GSE5522, GSE5520, GSE1491 [33], GSE2169, GSE2473), hgu133a (human, 72 chips; GSE1420 [34], GSE1922), hgu95av2 (human, 51 chips; GSE1563 [35]), hgu95d (human, 22 chips; GSE1007 [36]), hgu95e (human, 21 chips; GSE1007), mgu74a (mouse, 60 chips; GSE76, GSE1912 [37]), mgu74av2 (mouse, 29 chips; GSE1947 [38], GSE1419 [39,40]), moe430a (mouse, 10 chips; GSE1873 [41]), mouse4302 (mouse, 20 chips; GSE5338 [42], GSE1871 [43]), rae230a (rat, 26 chips; GSE1918, GSE2470), and rgu34a (rat, 44 chips; GSE5789 [44], GSE1567 [45], GSE471 [46]). These experiments cover many of the species commonly analyzed using the GeneChip platform, and were selected to represent a variety of tissue types and experimental treatments.

The BioConductor rma() function was used to perform probeset summarization, background subtraction and quantile normalization, with each raw intensity (.CEL) file preprocessed together with the other chips from the same GEO experiment. A variety of quality control indicators, listed in Table 1, were then computed for each chip. A list of all the .CEL files and their GEO identifiers, along with quality control feature scores and expert annotations, can be found in additional file 2. Also included in the file are descriptions explaining how each of the 29 quality control feature scores is computed from the raw expression data.

**Table 1:** BioConductor Quality Control Statistics

**Quality Statistic**^1^	**Description**
*mean.raw.int, sd.raw.int, median.raw.int, interQuartile.raw.int*	mean, standard deviation, median and inter-quartile range of raw log intensity distribution.

*q.5.raw.int, q.95.raw.int*	5th and 95th percentile of raw log intensity distribution.

*slope.bias, p.bias*	slope parameter and associated p-value of linear regression of log expression level versus probe number, as computed by R affy library function AffyRNAdeg().

*mean.norm.int, sd.norm.int, median.norm.int, interQuartile.norm.int, q.5.norm.int, q.95.norm.int*	mean, standard deviation, median, inter-quartile range, and 5th and 95th percentiles of normalized log intensity distribution.

*PLM.w.q.0.001, PLM.w.q.0.01, PLM.w.q.0.1, PLM.w.q.0.2*	0.1th, 1st, 10th and 20th percentile of the probe-level model weights, computed using affyPLM library functionality.

*PLM.res.q.0.01, PLM.res.q.0.1, PLM.res.q.0.25, PLM.res.q.0.75, PLM.res.q.0.9, PLM.res.q.0.99*	1st, 10th, 25th, 75th, 90th, and 99th percentile of probe-level model residuals, computed using affyPLM library functionality.

*RLE.median, RLE.interQuartile, RLE.lower.whisker, RLE.upper.whisker*	median, inter-quartile range, lower tail and upper tail of "relative log intensity", computed using affyPLM library functionality.

^1. ^The "SCORE" function was used to normalize values for each statistic, *t*, for each chip, *i*, relative to the values observed in other chips from the same experiment: ; with *median*() and *mad*() computed across all chips in the experiment.

The second dataset consists of all of the exon array .CEL files available in the GEO database at the time of this analysis (540 .CEL files). Fourteen different experiments are represented (GSE10599 [47], GSE10666 [48], GSE11150 [49], GSE11344 [50], GSE11967 [51], GSE12064 [52], GSE6976 [53], GSE7760 [54], GSE7761 [55], GSE8945 [56], GSE9342, GSE9372 [57], GSE9385 [58], GSE9566 [59]). The dataset includes examples of the Mouse Exon 1.0 ST array and several versions of the Human Exon 1.0 ST array. This dataset was processed using two different methods. First, the same set of quality indicators described above for the 3' expression dataset was prepared using the BioConductor packages in R. The "aroma" .cdf annotation files [60] were used to read in expression values for the core probes on the arrays. In addition, this second dataset was also processed using the Affymetrix Expression Console software. Only the "core" probesets were considered and the software was used to perform "gene-level" probeset summarization, background subtraction and quantile normalization using the "RMA sketch" option in the software. Several alternative quality indicators were then computed (Table 2). A list of the .CEL files and their GEO identifiers and also the various quality control feature scores is included in additional file 3. Detailed descriptions of the Affymetrix Expression Console quality control features can be found in [61].

**Table 2:** Affymetrix Expression Console Quality Control Statistics (Exon Arrays)

**Quality Statistic**^1^	**Description**
*pm.mean*	mean of the raw intensity for all PM probes, prior to any normalizations.

*bgrd.mean*	mean of the raw intensity for all probes used to compute background intensity. (Note: may be higher than pm.mean because GC compositions of probes used to compute background and PM probes can be quite different.)

*pos.vs.neg.auc*	area under ROC curve discriminating between positive control probesets and negative control probesets.

*probeset.mean, probeset.stdev*	mean and standard deviation of probeset signals after normalization. ^2^

*probeset.mad.residual.mean, probeset.mad.residual.stdev*	mean and standard deviation of the absolute deviations of the RMA probe level model residuals from the median across chips. ^2^

*probeset.rle.mean, probeset.rle.stdev*	mean and standard deviation of the absolute values of the relative log expression (RLE) for all probesets. ^2^

^1. ^The "SCORE" function was used to normalize values for each statistic, *t*, for each chip, *i*, relative to the values observed in other chips from the same experiment: ; with *median*() and *mad*() computed across all chips in the experiment.

^2. ^Separate statistics are computed for a) all probesets, b) negative control probesets, and c) positive control probesets.

### Expert Annotation

A domain expert analyzed the 3' expression dataset (dataset 1) and assigned quality scores according to a procedure which is based on experience gained during almost three years of bioinformatics support within the Lausanne DNA Array Facility (DAFL). This quality control procedure is described in [15]. Briefly, the chip scan images and the distributions of the log scale raw PM intensities are visualized. Smaller discrepancies between chips are common and can often be removed by normalization. Remaining discrepancies usually indicate low quality data, possibly caused by problems in the amplification or labelling step. The general 5' to 3' probe intensity gradient averaged over all probe sets on a chip is also examined. The slope and shape of the resulting intensity curves depend on the RNA sample source, the amplification method, and the array type. In general, the specific shape of the curves is less important for the quality check than their agreement across the experiment. Pseudo-images representing the spatial distribution of residuals and weights derived from the probeset summarization model are very important diagnostics. Small artifacts are not critical when using robust analysis methods; however, extended anomalies are taken as an indication of low quality. In addition, box plot representations of the Normalized Un-scaled Standard Error (NUSE) from the probe level model fit and the Relative Log Expression (RLE) between each chip and a median chip are examined. These plots are used to identify problematic chips showing an overall deviation of gene expression levels from the majority of all measured chips. A chip may be judged as having poor quality if it is an apparent outlier in the experiment-wide comparison of several quality measures. Each array was given a quality score of 0, 1 or 2, with 0 being "acceptable quality" (519 chips), 1 being "suspicious quality" (56 chips) and 2 being "unacceptable quality" (28 chips). For the purposes of classification, chips with scores of 1 or 2 were combined into the composite "low quality" class.

### Supervised Naïve Bayes Classifier

Previous research has demonstrated that quality assessment of microarray data can be successfully automated with the use of a supervised classifier [15,20]. The goal of supervised classification is to utilize an annotated training dataset to learn a function that can be used to correctly classify unlabeled instances. In the case of microarray quality assessment, the training dataset consists of the quality control features computed for each chip, combined with the quality annotation for each chip.

By making the simplifying assumption that all features are conditionally independent, naïve Bayes classifiers attempt to directly model the probability that a particular data point belongs to each class. Given the class label, each feature is assumed to follow an independent, univariate distribution. These distributions are, of course, unknown, but the maximum likelihood parameter estimates can be determined from a labeled training set. Then, for each unlabeled instance, Bayes' rule can be applied to compute the conditional probability that the instance belongs to each of the possible classes. Because we had prior success performing classification on a similar data set using Naïve Bayes with Gaussian feature distributions [15], we again chose to model the features using independent normal distributions. However, the approach could easily be adapted to use alternative distributions, for example, Student's t-distribution or the skew-normal distribution.

Under this framework, the probability that an unlabeled instance belongs to the low quality class is estimated as follows:

(1)

where *c *∈ {0,1} signifies the class label, with 0 denoting "high quality" and 1 denoting "low quality,"  is a length *p *vector of features describing the unlabeled instance., and  is the Gaussian density for the *i*^th ^feature, among low quality chips.

The marginal probability of observing a low quality chip, Pr{*c *= 1}, can be estimated from the proportion of low quality chips in the training set. Furthermore, the marginal density for a particular combination of feature values, , independent of the class label, is equal to:

(2)

For the purposes of classification, this algorithm assigns class 1 to an unlabeled instance , if Pr(*c *= 1|} > *t*, where *t *is a threshold parameter, ordinarily set to 0.5 in order to approximate the Bayes optimal decision rule. By varying this parameter, it is also possible to construct ROC curves which display the tradeoff between sensitivity and specificity for various decision thresholds.

### Unsupervised Naïve Bayes Classifier

The standard (supervised) approach to constructing a naïve Bayes classifier employs maximum likelihood estimation to infer the distribution parameters of each classification feature from an expert-annotated training set. It is, however, also possible to construct an "unsupervised" naïve Bayes classifier by using an unannotated dataset as input. In this case, the EM algorithm is used to infer the feature distributions, assuming an appropriate Gaussian mixture model, as described in the following section.

### Gaussian Mixture Model and the EM Algorithm

The naïve Bayes classification model described above requires parameter estimates for the quality control metrics, conditional on each quality class. In the absence of annotated data, however, the quality classes of the unannotated training instances are additional unknowns that must be estimated along with the distributional parameters. We model the unannotated dataset using a Gaussian Mixture Model, under the assumption that microarray data can be reasonably classified into the dichotomy of "high quality" and "low quality" chips, and that the unlabeled training set contains examples of each.

Given a large set of microarray data files, the first step is to compute values for each of the various quality control features. Then, for each feature, we assume that the observed distribution of scores is generated by an underlying Gaussian mixture model with two components: 1) chips having high quality and 2) chips having low quality. Given the mixture component, *c *∈ {0,1}, each feature is assumed to follow a Normal  distribution. However, in the case of an unlabeled dataset, the true mixture component is unknown. We further assume that, marginally, the class label for each instance is a simple Bernoulli random variable with probability *φ *of indicating a low quality chip. Under this model, the (log) likelihood of the dataset is:

(3)

where:

• ***x ***is an *N *× *p *matrix containing the *p *feature values for the *N *items in the dataset, with  denoting the length *p *feature vector for the *i*^th ^data point.

• ***μ ***is a 2 × *p *parameter matrix containing, in each column, *μ*_0 _and *μ*_1 _for the *p*^th ^feature;  is the length *p *parameter vector for the *j*^th ^Gaussian mixture component (*j *∈ {0,1}).

• ***σ***^2 ^is a 2 × *p *parameter matrix containing, in each column,  and  for the *p*^th ^feature;  is the length *p *parameter vector for the *j*^th^Gaussian mixture component.

•  is a length *N *vector containing the (unknown) class labels for each of the *N *data points.

•  is a length 2 probability vector containing the probability that a randomly chosen data point belongs to each class.

The likelihood function in equation 3 can be maximized using the EM algorithm [21]. The EM algorithm is a well-known method for maximizing mixture model likelihood functions by iteratively performing two steps:

• **E Step**: Estimate the unknown class labels, based on the current estimates for the other parameters.

• **M Step**: Given current class labels, compute the maximum likelihood estimators for the parameters ***μ***, ***σ***^2^, and .

To implement the EM algorithm, we introduce an additional *N *× 2 matrix, ***w***, which contains, for each data point, *i*, the current guesses for p( = 0) and p( = 1). After initializing all parameters and the weight matrix, *w*, to random values, the EM algorithm proceeds as follows:

**M **step: For *j *∈ {0,1}, *k *∈ {1 ... *p*}

(4)

(5)

(6)

**E Step**: For *i *∈ {1 ... *N*}, *j *∈ {0,1}

(7)

where *normpdf*(*x*, *μ*, *σ*^2^) denotes the probability density of a normal distribution evaluated at *x*. Because the algorithm can possibly converge to local optima, it is prudent to run the algorithm several times after random restarts. Additionally, each  was constrained to be > = .001 to avoid convergence to a trivial solution. Further details concerning this implementation of the EM algorithm and the associated Gaussian mixture model can be found in [62]. Once estimates have been obtained for ***μ***, ***σ***^2 ^and , any unlabeled instance can be classified according to these mixture components using naïve Bayes, according to equation 1 (or equivalently, equation 7, in the case of the original unlabeled dataset). Since our assumption is that low quality chips are outliers with respect to these quality features, we use the mixture component corresponding to the smallest value from  to identify the low quality class.

### Feature Selection

In order to achieve optimal classification performance, it is important to select an appropriate subset of the classification features. Ideally, this subset should include independent features that are each individually predictive of the class label.

To measure the ability of each feature to predict the correct class label in a training set (where "correct" label is defined as either the expert annotation in the supervised case, or the estimated ***w ***matrix in the unsupervised case), we first constructed an *N *× *p *score matrix, *S*, where each cell *S*_*ij *_contains a distance measuring the discrepancy between the true and predicted class for data point , given the *j*^th ^feature and the parameter estimates for that feature:

(8)

Then for each feature, *j*, these scores were totaled across all *N *data points

(9)

Finally, the *p *scores were sorted in ascending order, to rank the features by their ability to predict the correct class label. Denote the rank of feature *j *according to the value of this score as *S*_[*j*]_.

To identify correlations among the quality control features, we next computed the *p *× *p *Pearson correlation matrix. Let *ρ*_*jk *_denote the correlation between features *j *and *k*, and *ρ*_[*j*]*k *_represent the *rank *of the correlation of feature *j *with feature *k *among all other features correlated with *k*, with features ranked in order of *descending *correlation. To select a subset of n features, we used the following forward selection algorithm:

• First, select the single feature that is most predictive of the class labels, i.e. the feature with *S*_[*j*] _= 1.

• Then, sequentially, for the remaining *n*-1 features, select the feature *j *to satisfy:

(10)

where *F *denotes the set of previously selected features. The constants *c*_1 _and *c*_2 _in this expression are weighting factors that can be modified to control the tradeoff between selection for independent features and features that are highly correlated with the class label. We used 0.5 for each.

## Results and discussion

### Parameter Estimates

#### 3' Expression Arrays

**Figure 1:**
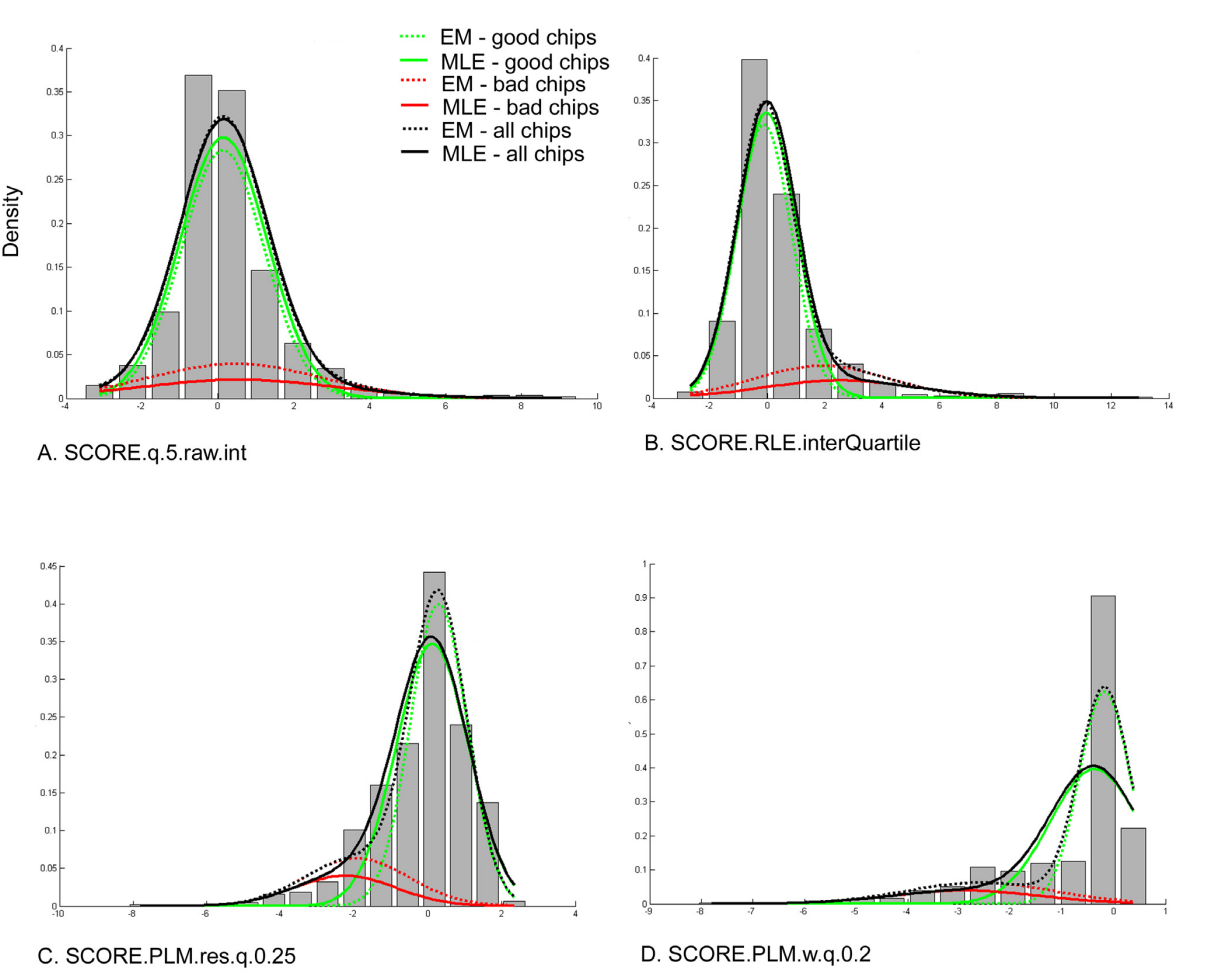
**Mixture Model Parameter Estimates**. Supervised (MLE) and Unsupervised (EM) estimates shown are for the following features from the 3' expression arrays: (A) 5th percentile of raw intensities, (B) inter-quartile range of the Relative Log Intensity (RLE), (C) 25th percentile of the probe-level model residuals, and (D) the 20th percentile of the probe-level model weights. All features were normalized relative to other chips in the same experiment, using the SCORE function (see Table 1).

We applied the unsupervised mixture model described above to the 3' expression array data (hiding the expert quality labels). For nearly all of the 29 quality control features considered, the unsupervised EM parameter estimates very closely approximate the corresponding supervised MLE estimates, a result which indicates that the unsupervised approach was able to discover patterns in the data that are in agreement with the expert annotations. Additional file 4 contains the mixture model parameter estimates for , *μ*_0_, *μ*_1_,  and  for each of the quality control features. These estimates were obtained by applying the EM algorithm to the entire unlabeled dataset. For comparison, the table also includes the maximum likelihood estimates obtained using the expert-annotated class labels. Figure 1 shows some representative examples. Plots of this nature reveal that, in most cases, the EM and (supervised) MLE estimates exhibit only minor differences, generally with magnitudes analogous to the discrepancies shown in Figures 1a–d.

The EM estimates appear to be reasonable in all cases, given the original intent of each quality metric. For example, given the normalized (log-scale) expression values, the RLE metric measures the distribution of the quantity  for each chip, where  is the log expression measurement for probeset *g*, on chip *i*, and *m*_*g *_is the median expression of probeset *g *across all arrays. In general, since it is ordinarily assumed that the majority of genes are not differentially expressed across chips, the quantity *M*_*gi *_is expected to be distributed with median 0. In addition, chips that more frequently have extreme expression values will have a large inter-quartile range for this statistic. Figure 1b indicates that, as expected, low quality chips were indeed more likely to have a large inter-quartile range for the RLE statistic.

Parameter estimates for the other metrics also agree with our expectations. For example, the estimates for metrics relating to probe-level model weights and residuals reflect the expectation that low quality chips should have larger residuals and more down-weighted probesets (Figure 1c, d). Similarly, the estimates indicate that low quality chips are more likely to have RNA degradation plots that are different from other chips in the same experiment. The low quality chips also tend to have both mean raw and mean normalized intensities that are either significantly higher or lower than other chips in the same experiment.

#### Exon Arrays

The Affymetrix exon array platform is different from the 3' expression array platform in several important ways [63]. For example, the 3' expression array targeting the human genome (Hgu133) has, on average, 1 probeset pair for each well-annotated gene; each probeset consists of 11 individual 25-mer probes, which primarily target the 3' region of the gene. In contrast, the Human Exon 1.0 ST array has 1 probeset for each exon for each gene in the target genome. Each probeset contains, in general, 4 (rather than 11) 25-mer probes. Unlike 3' expression arrays, exon arrays lack mismatch probes. Instead, the background expression level for each probe is estimated by averaging the intensities of approximately 1000 surrogate genomic and anti-genomic background probes having the same GC content as the target probe. Because most genes consist of several exons, the median number of probes per gene is increased on the exon array from 11 on the 3' array to between 30–40 [64]. However, genes with fewer exons are covered by fewer probes. In fact, there are a few thousand well-annotated single exon genes covered by only 4 probes [63]. Furthermore, the feature size on the exon arrays has been reduced from 11 × 11 microns on the HGU133 array to 5 × 5 microns on the Human Exon 1.0 ST array (about 1/5 the area). This change may increase the expression variance, at least at the probeset level [63]. Exon arrays also utilize a different hybridization protocol which uses sense-strand labeled targets, and results in DNA-DNA hybridizations rather than the DNA-RNA hybridizations used with traditional 3' arrays [65]. These differences suggest that the distributions of key quality control indicators may differ between the two platforms.

**Figure 2:**
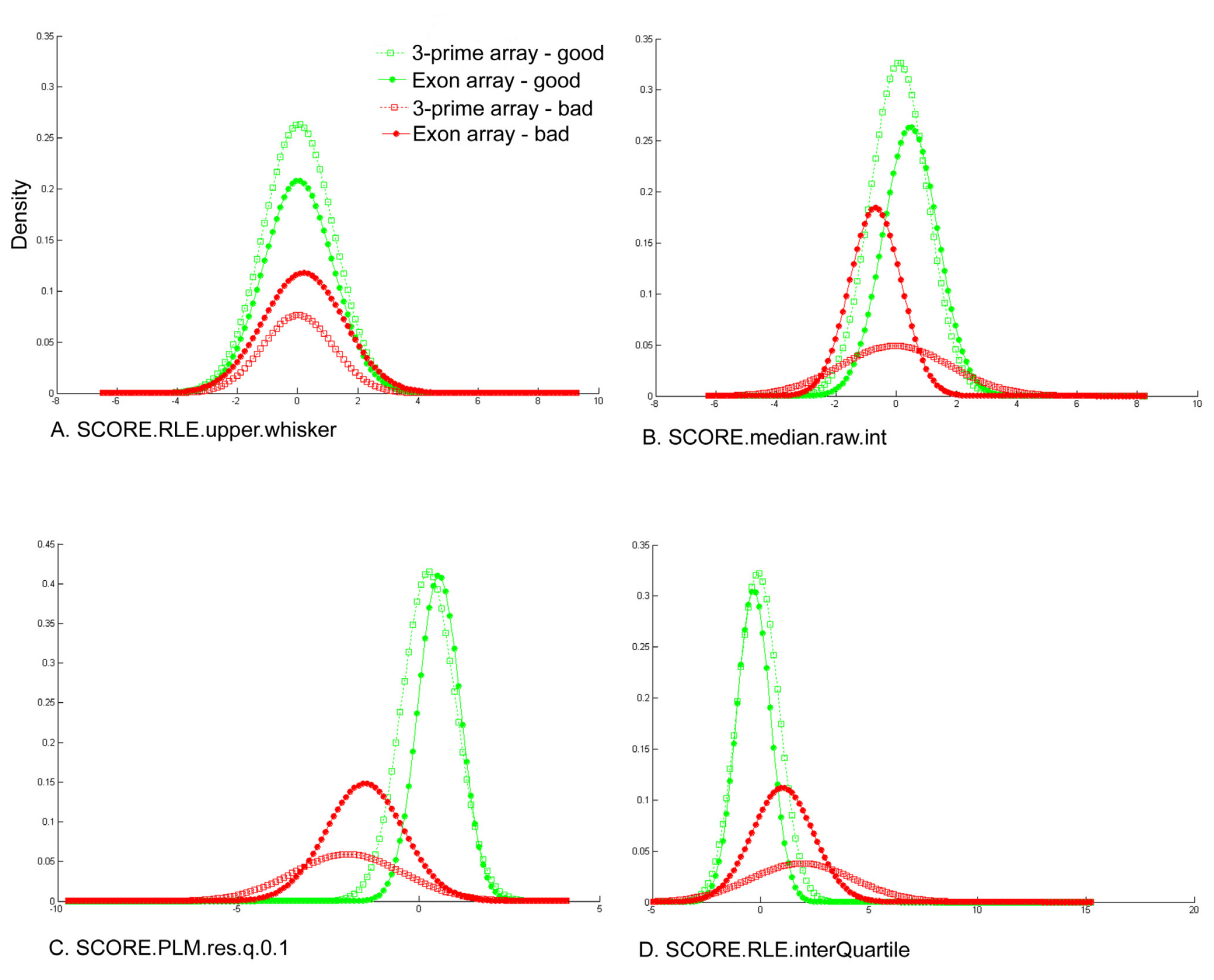
**Comparison of Parameter Estimates for 3' Expression Arrays and Exon Arrays**. Each diagram illustrates the unsupervised Gaussian parameter estimates for one of the quality control features, for each of the two chip types. Estimates shown are for the following features: (A) Upper tail of the Relative Log Intensity (RLE), computed using the affyPLM functionality, (B) median of the raw intensity distribution, (C) 10th percentile of the probe-level model residuals, and (D) inter-quartile range of the RLE.

For the exon arrays, the resulting probability estimate for low quality chips was .397 – nearly twice what was obtained for the 3' arrays. This is reflected in Figure 2 as the larger areas under the red curves for exon arrays compared to 3' arrays, and as the smaller areas under the green curves for exon arrays compared to 3' arrays. For the majority of the indicators, the estimated distributions were qualitatively similar to those estimated for the 3' arrays (Figure 2a, c, d). One interesting difference is that in the exon arrays, the low quality chips appear to be more likely to have median raw intensity values that are lower than other chips in the same experiment (Figure 2b), whereas for the 3' arrays, both abnormally high and low median raw intensities appear to be indicative of bad chips.

**Figure 3:**
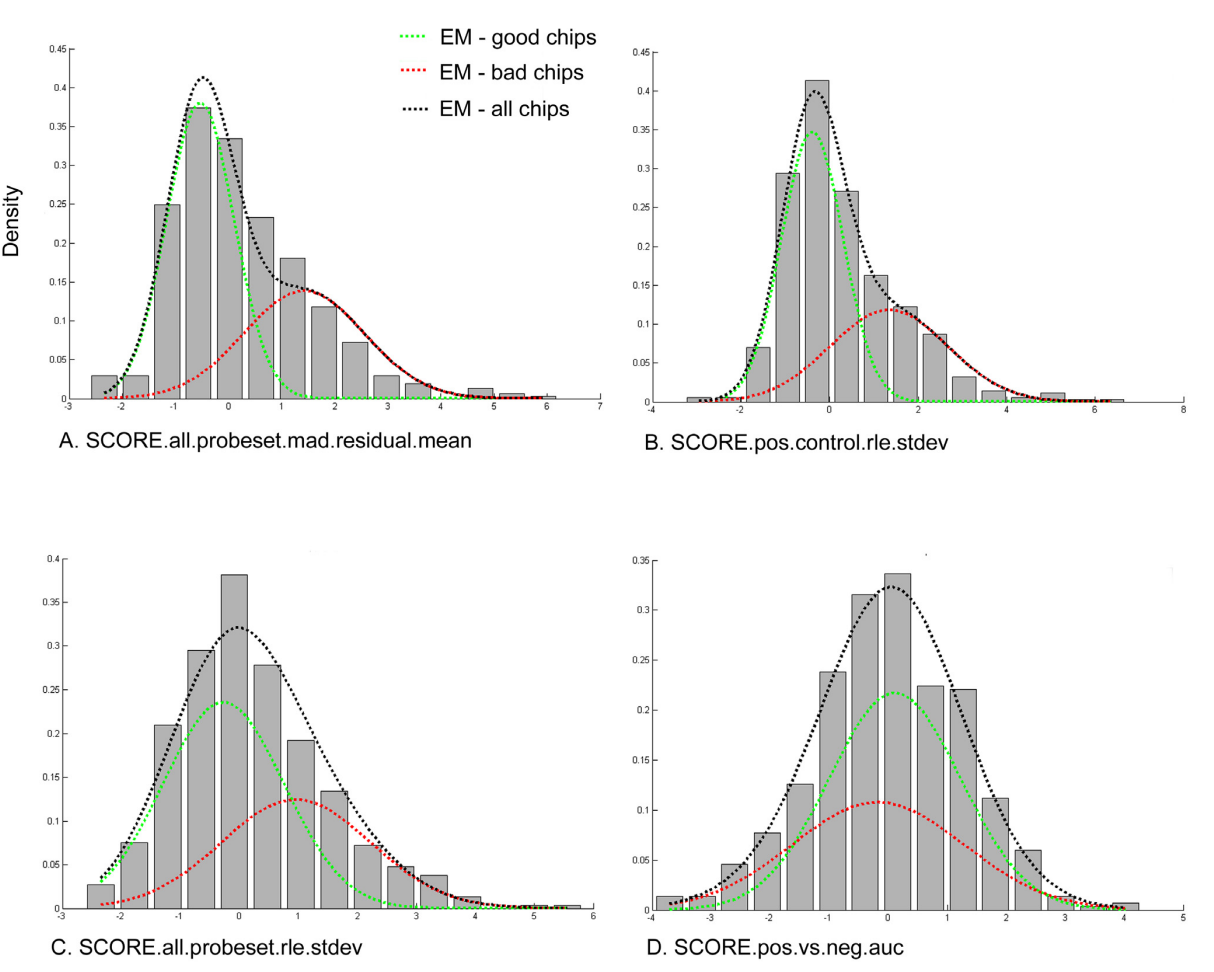
**Parameter Estimates for Exon Array Expression Console QC Features. **Shown are the parameter estimates obtained using the EM algorithm for various exon array quality control features available in the Affymetrix Expression Console software. Estimates shown are for the following features: (A) mean of the absolute deviation of the RMA probe level model residuals from the median across chips, (B) standard deviation of signal from positive control probesets after normalization, (C) standard deviation of signal from all probesets after normalization, and (D) area under ROC curve discriminating between positive control probesets and negative controls.

To check the robustness of our estimates, we also analyzed a separate set of quality control indicators (Table 2) computed using the Affymetrix Expression Console software. In agreement with the estimate obtained using the first set of quality metrics, the inferred probability for low quality chips was .394 using the Expression Console quality indicators. At a qualitative level, the estimates for the Expression Console quality indicators generally agreed with our expectations. For example, Figure 3a shows that, as expected, lower quality chips tend to have larger residuals when fitting the RMA probe-level summarization model. Similarly, Figures 3b and 3c show that low quality chips are more likely to have higher variability in the RLE metric. Interestingly, the SCORE.pos.vs.neg.auc metric, which measures the area under an ROC curve discriminating between positive and negative controls, did not indicate a major difference between high and low quality chips. This seems to be in conflict with the recommendation by Affymetrix that this is potentially one of the most useful quality control indicators for exon arrays [61]. This observation could reflect the fact that labs detecting unusual values for this metric may have been more likely to exclude the corresponding chips from further analysis.

### Classifier Performance Evaluation

#### 3' Expression Arrays

**Figure 4:**
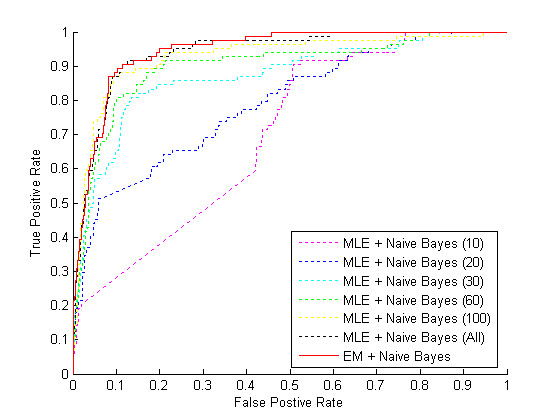
**Classifier Performance**. Unsupervised versus supervised classifier using labeled data sets of various sizes. When the full labeled training dataset (~540 labeled instances per fold) is available, the performance of the unsupervised classification method (EM+Naïve Bayes) and the supervised classification method (MLE+Naïve Bayes) are equivalent on the test dataset. When the amount of labeled data is limited, but unlabeled data is abundant, the unsupervised method outperforms the supervised method.

After obtaining parameter estimates for various quality control features for the 3' expression arrays, we next sought to compare the performance of the unsupervised and supervised classifiers. A 10-fold cross-validation procedure was used to compare the performance of naïve Bayes classifiers constructed using distribution parameters estimated using either the standard maximum likelihood method or, alternatively, the unsupervised mixture model approach. For each of 10 iterations, 9/10 of the 603 data instances were used as a training set, for both parameter estimation and also the selection of 5 classification features. For classifiers built using supervised MLE estimation ("MLE + Naïve Bayes"), the expert generated labels were used to distinguish between high and low quality chips in the training set. For the unsupervised classifier ("EM + Naïve Bayes"), the expert labels in the training set were ignored and the EM algorithm was used to estimate parameters of a Gaussian mixture model. The remaining unused 10^th ^of the data was used to assess the performance of the classifier, using the expert labels as the standard of truth. The performance of the two algorithms was nearly identical. The confusion matrices (additional file 1: Table S1) show the classification results for the two algorithms using a classification threshold of 0.5. The accuracy of the MLE + Naïve Bayes method was .907 with a false positive rate of .058, while the accuracy of the EM + Naïve Bayes method was .910 with a false positive rate of .079. An ROC curve, constructed by varying the classification threshold, is shown in Figure 4. The area under the ROC curve (AUC) was .9455 for the unsupervised method and .9402 for the supervised method. Although this performance is good, it is possible that these results could be improved even more by identifying and using alternative (other than normal) distributions to model one or more of the classification features.

In many real world scenarios the amount of unlabeled data available greatly exceeds the amount of expert-labeled data. To test the performance of the two classifiers under these conditions, we performed additional 10-fold cross-validation experiments similar to the previous test. However, in this case, the supervised MLE + Naïve Bayes classifier was trained using random subsets of instances from each labeled training fold, while the EM + Naïve Bayes classifier was constructed using the entire unlabeled training fold. Subsets containing 10, 20, 30, 60, 75, and 100 instances were used to train the supervised classifier. The ROC curves in Figure 4 indicate that the EM + Naïve Bayes classifier appears to have an advantage when the amount of unlabeled training data available greatly exceeds the amount of expert-labeled data. For example, the unsupervised method clearly outperforms the supervised method when 30 or fewer labeled instances were available. Table S2 (available in additional file 1) contains the resulting confusion matrix for the case in which 30 labeled training instances were used, with a classification threshold of 0.5.

#### 3' Exon Arrays

To demonstrate the general applicability of our method, we constructed unsupervised classifiers using the two sets of quality control variables and the entire unlabeled training set. These classifiers were then used to predict classification labels for each data point. Figure S6 (in additional file 1) shows a Venn diagram comparing the classification results for classifiers constructed using the BioConductor quality features and the Expression Console quality features. In most, but not all, cases, the classifiers agree on the characterization of each chip with regard to quality. In addition, both classifiers agree that approximately 39% of the data is low quality. Additional file 3 contains the classification labels obtained using unsupervised classifiers constructed using each set of quality variables.

### Simulation Results

The agreement between the quality control feature distribution parameters estimated using the supervised maximum likelihood method and the estimates obtained with the unsupervised Gaussian mixture model suggests that our domain expert has uncovered a plausible dichotomy of chips within our dataset. To further confirm that the chips classified as having low quality were indeed more likely to negatively impact tests for differential expression, we performed a simple simulation. The procedure involved adding an offset to the observed expression measurements for a subset of the probesets on a set of "treatment" arrays, and then comparing these arrays with a set of unmodified "control" arrays sampled from the same experiment (details not shown). Among those chips designated by the expert as low quality, the majority (approximately 70%) impaired the ability to detect simulated differential expression when included in an analysis, compared to only about 10% of the chips classified as having high quality.

## Conclusion

In this paper we have illustrated the efficacy of an unsupervised classification approach to assessing microarray data quality. Our method uses unlabeled training data to identify apparent distinctions between "good" and "bad" quality chips within the dataset. The method then integrates measurements obtained across a variety of quality dimensions into a single composite quality score which can be used to accurately identify low quality data.

Our method is flexible and can be easily adapted to accommodate alternate quality statistics and platforms. Because this technique requires only unannotated training data, it is easy to keep the resulting classifier up-to-date as technology evolves, and the adaptable nature of the system makes arbitrary, universal quality score thresholds unnecessary. Moreover, since a naïve Bayes classification approach involves the estimation of the underlying, univariate distributions for each of the classification parameters, this method allows for intuitive explanations that offer an advantage over other "black box" classification systems [66,67]. For example, under this framework, it is possible to infer which diagnostic plots and features are most relevant for the classification of a particular chip. These plots can then be presented to the user in order to explain the classification. A quality control method that incorporates an interpretation of standard diagnostic plots is an extension of a familiar process already used by many labs, and good diagnostic plots can provide powerful and convincing evidence of data quality artifacts.

An important caveat for this, and any quality control methodology, is that the decision about what to do with the detected low quality chip(s) is dependent on the experimental design, the number of low quality chips detected, and the magnitude of the defects encountered. In many cases, low quality chips still contain valuable information, and in some cases the most effective strategy may be to simply down-weight these chips rather than discarding them entirely [68].

Nevertheless, with the availability of a variety of rapidly growing public repositories for microarray data, the continual appearance of new microarray chip types, and the increasing usage of genomics data by research organizations worldwide, the development of robust and flexible methods for microarray quality assessment is now more important than ever. An advantage of the approach described in this paper is that, once a classifier has been constructed, the run-time required to automatically classify new instances is minimal. This makes the method ideal for use as a component of a batch processing system, such as a screening tool for use with public databases, or as a step in a meta-analysis pipeline.

## Availability and requirements

• ***Project name***: Unsupervised Assessment of Microarray Data Quality Using a Gaussian Mixture Model.

• ***Availability***: A Matlab implementation of these algorithms and the corresponding analyses is available in additional file 5.

• ***Operating system***: Implemented and tested under Windows XP.

• ***Programming language***: Matlab 7.0.1.15, service pack 1.

• ***Other requirements***: Matlab Statistics Toolbox version 6.1.

• ***License***: Brian E. Howard. Free for non-commercial use.

• ***Any restrictions to use by non-academics***: Contact corresponding author.

## Authors' contributions

BEH implemented the method and analyzed the data. BEH, BS, and SH conceived of the method and study design, and collaborated to prepare the manuscript. All authors approved the final manuscript.

## Supplementary Material

Additional file 1 **Additional File 1 – Supp Materials S1–S6**. Word document containing additional figures and tables. Click here for file

Additional file 2 **Additional File 2 – Training Data 3 Prime Arrays**. Excel workbook containing the .CEL file names and Feature Scores for "dataset 1" (3' expression arrays.). Click here for file

Additional file 3 **Additional File 3 – Training Data Exon Arrays**. Excel workbook containing the .CEL file names and Feature Scores for "dataset 2" (exon expression arrays.). Click here for file

Additional file 4 **Additional File 4 – Parameter Estimates**. Excel workbook containing parameter estimates obtained using the Gaussian Mixture Model (both datasets.). Click here for file

Additional file 5 **Additional File 5 – SourceCode**. Zipped archive contains Matlab source code used for the analyses described in this paper. See the file "READ_ME.txt" for instructions explaining how to run the code. Click here for file
