# Supplementary material for: Unsupervised assessment of microarray data quality using a Gaussian mixture model
Source: BMC Bioinformatics. 2009 Jun 22;10:191. doi: 10.1186/1471-2105-10-191 (PMC2717951; doi:10.1186/1471-2105-10-191)
Supplement: Additional file 1 — – Supp Materials S1–S6. Word document containing additional figures and tables. [file 1471-2105-10-191-S1.doc]

Supplementary Materials

**Table S1**. Confusion Matrices, Full Training Set

|  | **MLE + Naïve Bayes (Supervised)** | | |
| --- | --- | --- | --- |
|  | **0** | **1** | **◄ Expert Label** |
| **Classified As► 0** | 489 | 26 |  |
| **1** | 30 | 58 |  |
|  | **EM + Naïve Bayes (Unsupervised)** | | |
|  | **0** | **1** | **◄ Expert Label** |
| **Classified As► 0** | 478 | 13 |  |
| **1** | 41 | 71 |  |

**Table S2**. Confusion Matrices, Given 30 Labeled Instances for Supervised Method

|  | **MLE + Naïve Bayes (Supervised)** | | |
| --- | --- | --- | --- |
|  | **0** | **1** | **◄ Expert Label** |
| **Classified As► 0** | 492 | 44 |  |
| **1** | 27 | 40 |  |
|  | **EM + Naïve Bayes (Unsupervised)** | | |
|  | **0** | **1** | **◄ Expert Label** |
| **Classified As► 0** | 478 | 13 |  |
| **1** | 41 | 71 |  |


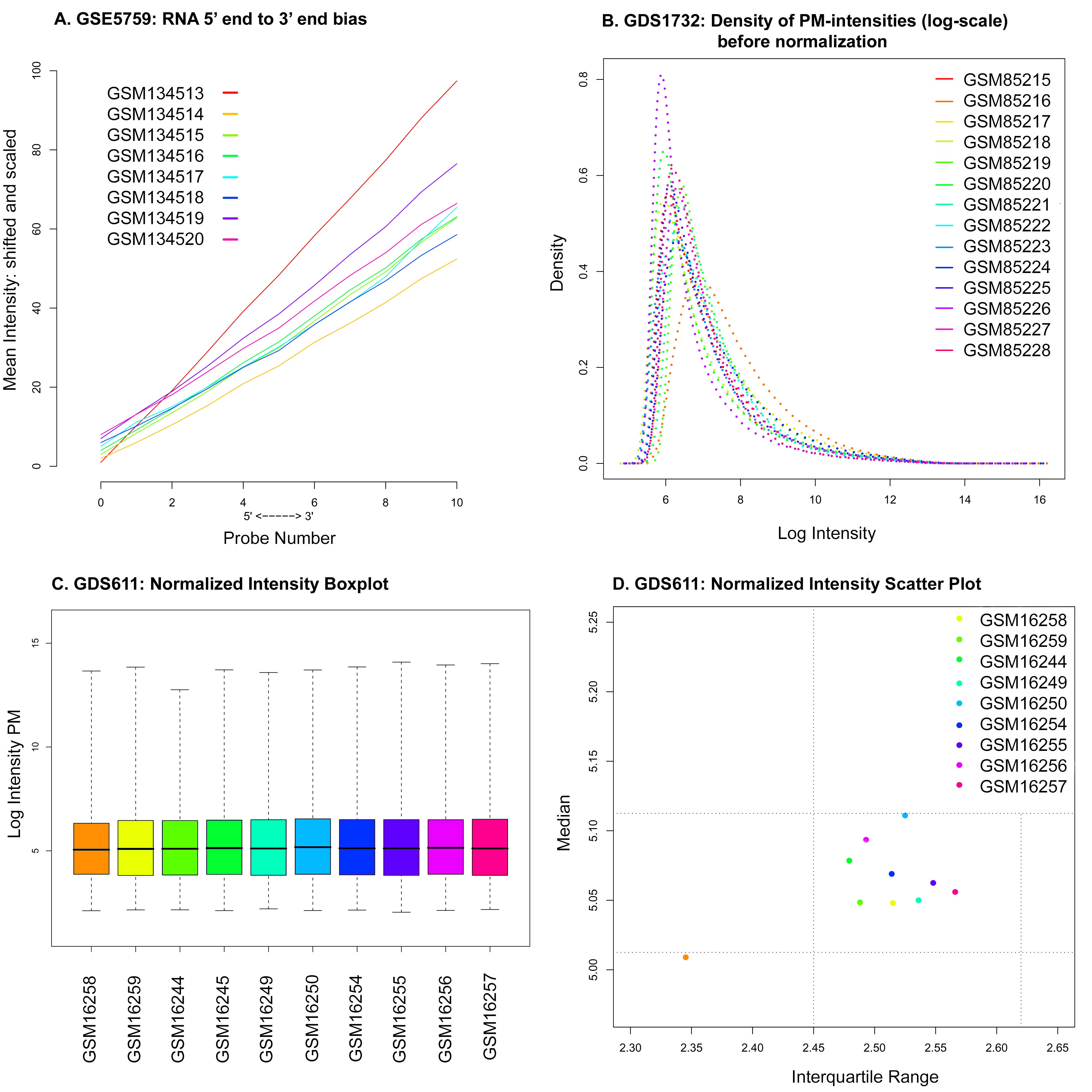


**Figure S3**. Examples of Microarray Quality Control Diagnostics

1. RNA degradation plot. The graph indicates that RNA degradation may be occurring at a different rate in sample GSM134513 compared to the other samples in the experiment. Inclusion of this sample in the analysis may adversely affect the resulting inferences.
2. Raw intensity distribution. A comparison of the un-normalized PM probe intensity distributions of the chips in an experiment can be used to identify outliers.
3. Normalized intensity box plot. After quantile normalization, the intensity distribution for all chips in an experiment is expected to be similar. Chips that deviate significantly from this expectation should be considered for exclusion.
4. Normalized intensity scatter plot. This plot is constructed from the same underlying data as (C). Differences among the chips suggest that sample GSM16258 may be an outlier.


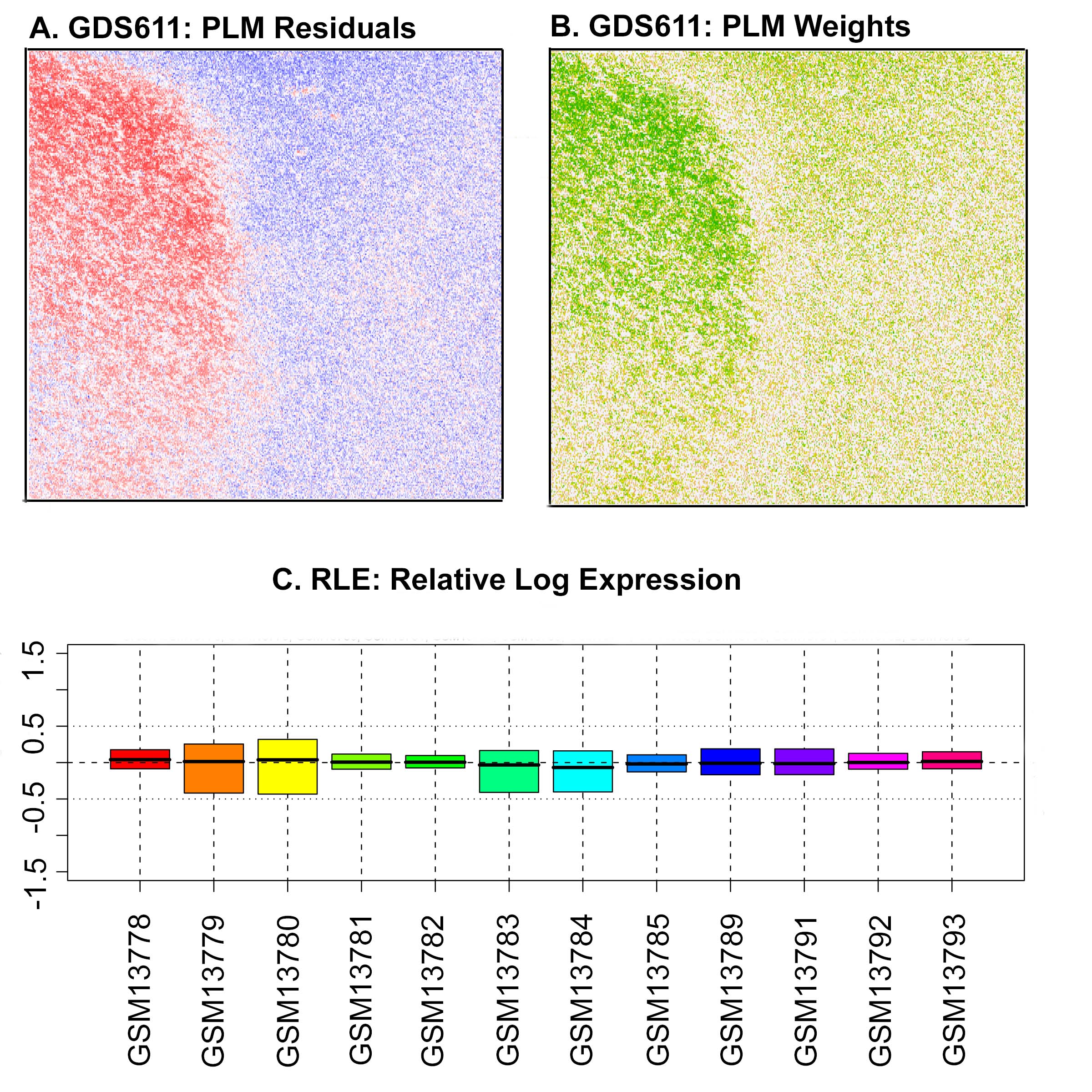


**Figure S4.** Additional Microarray Quality Control Diagnostics

1. Probe level model (PLM) residuals. The residuals in the probe level summarization model are expected to have a mean of zero. A large field of positive residuals (red) separated from a large field of negative residuals (blue) indicates a possible quality problem for sample GSM16258 from GEO dataset GDS611.
2. Probe level model weights. An excess of down-weighted probes (dark green area) in the probe level model for chip GSM16258 is an additional indicator of potential quality problems.
3. Relative Log Expression (RLE) box plot. Since most genes are not expected to be differentially expressed, a non-zero median and a large inter-quartile range in the RLE box plot can serve as indicators of low quality data.

**Figure S5.** Feature Selection Results

The histogram indicates the number of folds (out of 10) that each quality control classification feature was selected during testing of the EM+Naïve Bayes classification method. The feature most predictive of the class label in all 10 folds was SCORE.PLM.w.q.0.2 and hence this feature was always used as the first selection. A typical set of features selected is SCORE.PLM.w.q.0.2, SCORE.p.bias, SCORE.RLE.median, SCORE.PLM.res.q.0.1, and SCORE.PLM.res.q.0.25. Since each feature is chosen according to a trade-off between its usefulness in predicting the class label and its independence from previously chosen features, some of the features may not necessarily be optimal choices when used alone to predict the class label. It is possible to change the balance of the trade-off by modifying the weight constants in the feature selection expression (equation 10).

**Figure S6.** Exon arrays identified as high and low quality using two sets of QC indicators

The Venn diagram displays the number of exon arrays classified as low quality using the BioConductor QC variables and the Expression Console QC variables (left panel), as well as the number of exon arrays classified as high quality using the same two sets of QC indicators (right panel).
