# Supplementary figures and images for: Unsupervised assessment of microarray data quality using a Gaussian mixture model (part 2 of 2)
Source: BMC Bioinformatics. 2009 Jun 22;10:191. doi: 10.1186/1471-2105-10-191 (PMC2717951; doi:10.1186/1471-2105-10-191)

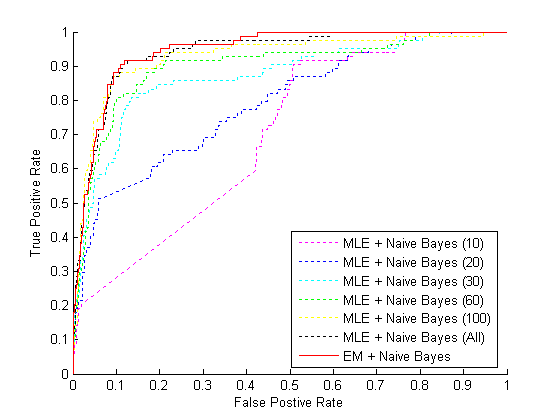

Supplement: Additional file 5 — – SourceCode. Zipped archive contains Matlab source code used for the analyses described in this paper. See the file "READ_ME.txt" for instructions explaining how to run the code. [file 1471-2105-10-191-S5.zip › Output/SmallDatasets.tif]
