# Supplementary figures and images for: Unsupervised assessment of microarray data quality using a Gaussian mixture model (part 1 of 2)
Source: BMC Bioinformatics. 2009 Jun 22;10:191. doi: 10.1186/1471-2105-10-191 (PMC2717951; doi:10.1186/1471-2105-10-191)

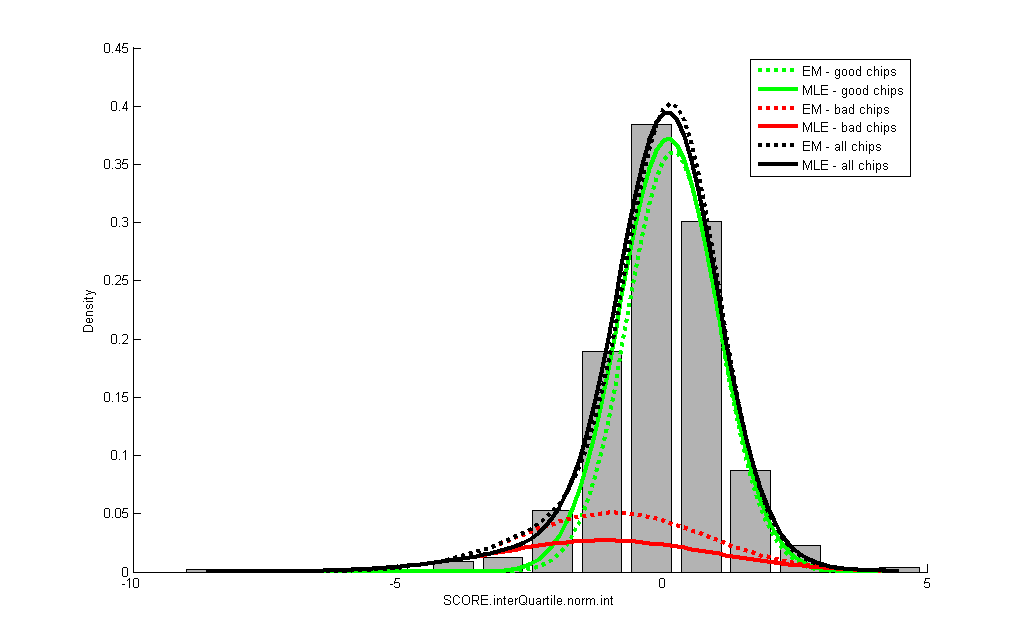

Supplement: Additional file 5 — – SourceCode. Zipped archive contains Matlab source code used for the analyses described in this paper. See the file "READ_ME.txt" for instructions explaining how to run the code. [file 1471-2105-10-191-S5.zip › Output/Distributions - 3-prime scores/SCORE.interQuartile.norm.int_Fig1.tif]

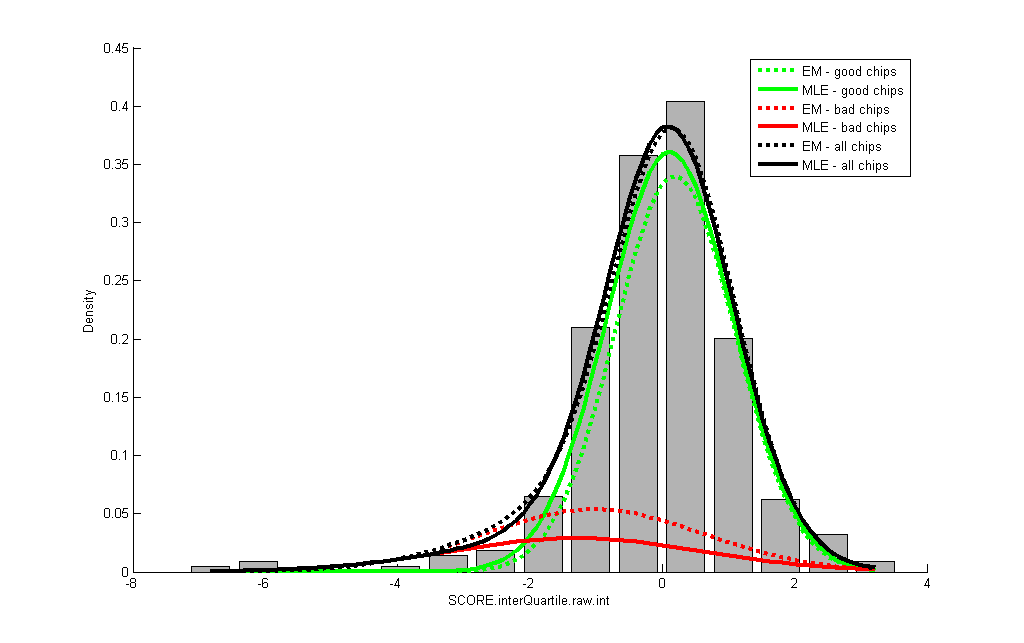

Supplement: Additional file 5 — – SourceCode. Zipped archive contains Matlab source code used for the analyses described in this paper. See the file "READ_ME.txt" for instructions explaining how to run the code. [file 1471-2105-10-191-S5.zip › Output/Distributions - 3-prime scores/SCORE.interQuartile.raw.int_Fig1.tif]

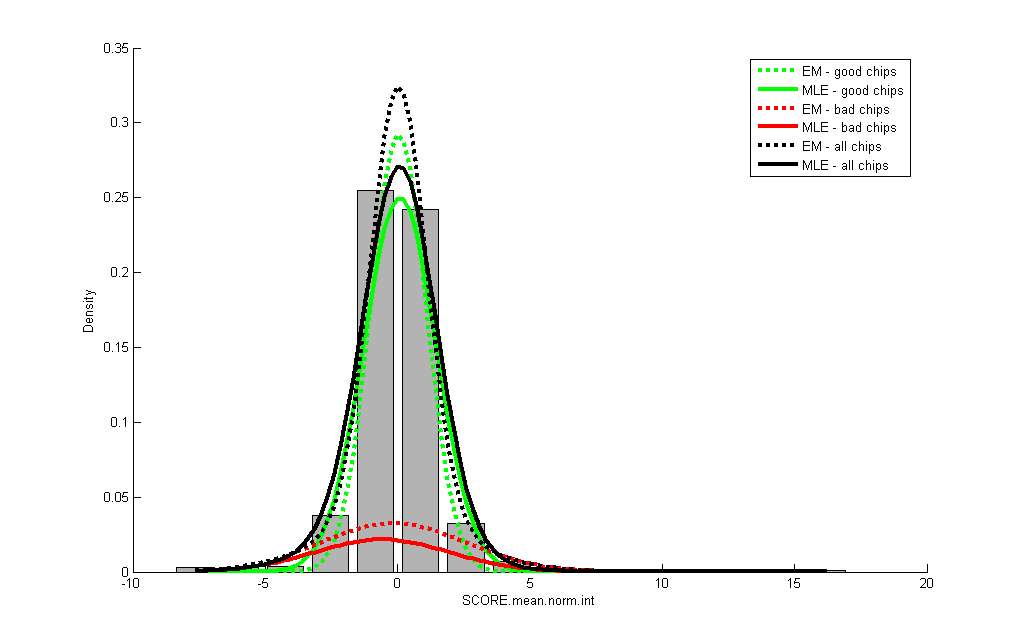

Supplement: Additional file 5 — – SourceCode. Zipped archive contains Matlab source code used for the analyses described in this paper. See the file "READ_ME.txt" for instructions explaining how to run the code. [file 1471-2105-10-191-S5.zip › Output/Distributions - 3-prime scores/SCORE.mean.norm.int_Fig1.tif]

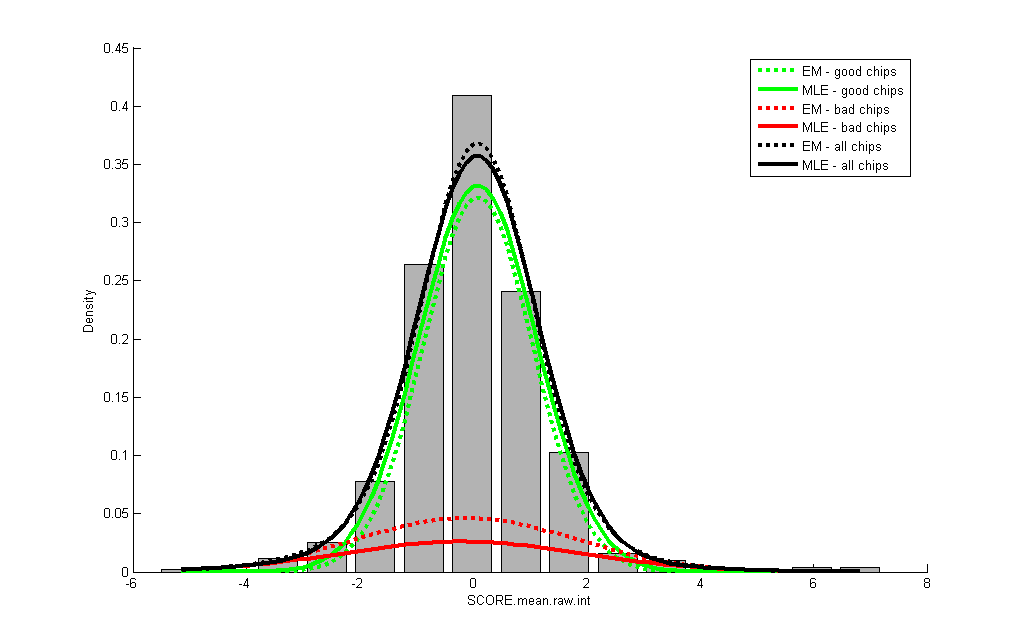

Supplement: Additional file 5 — – SourceCode. Zipped archive contains Matlab source code used for the analyses described in this paper. See the file "READ_ME.txt" for instructions explaining how to run the code. [file 1471-2105-10-191-S5.zip › Output/Distributions - 3-prime scores/SCORE.mean.raw.int_Fig1.tif]

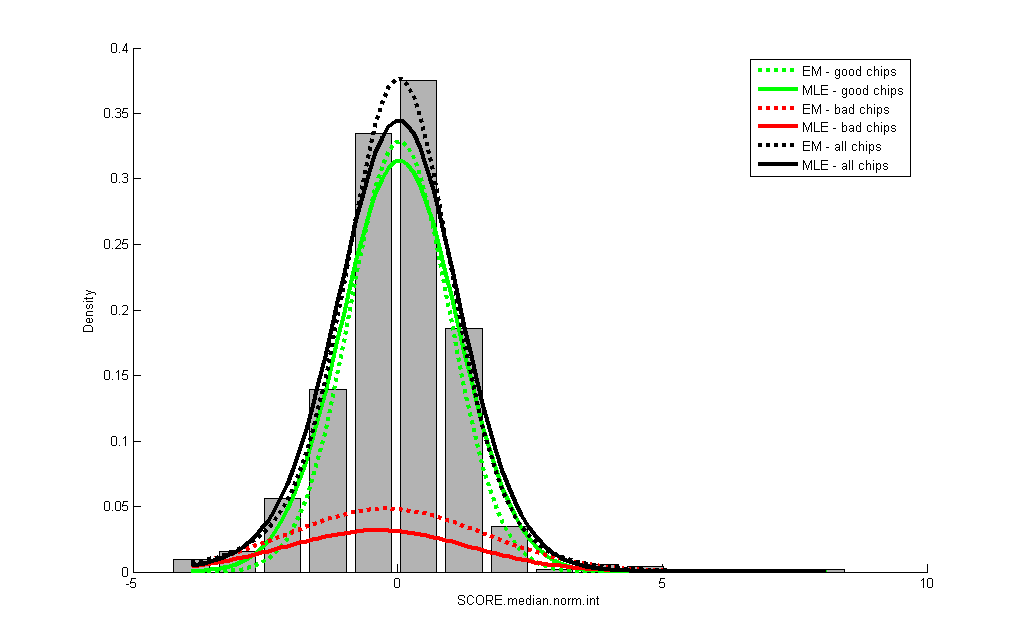

Supplement: Additional file 5 — – SourceCode. Zipped archive contains Matlab source code used for the analyses described in this paper. See the file "READ_ME.txt" for instructions explaining how to run the code. [file 1471-2105-10-191-S5.zip › Output/Distributions - 3-prime scores/SCORE.median.norm.int_Fig1.tif]

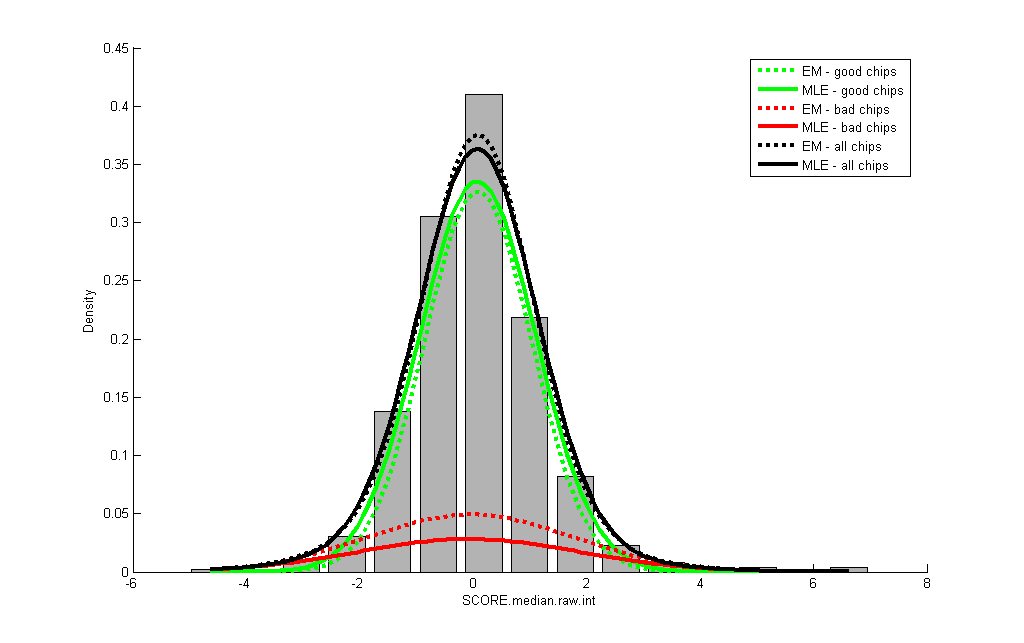

Supplement: Additional file 5 — – SourceCode. Zipped archive contains Matlab source code used for the analyses described in this paper. See the file "READ_ME.txt" for instructions explaining how to run the code. [file 1471-2105-10-191-S5.zip › Output/Distributions - 3-prime scores/SCORE.median.raw.int_Fig1.tif]

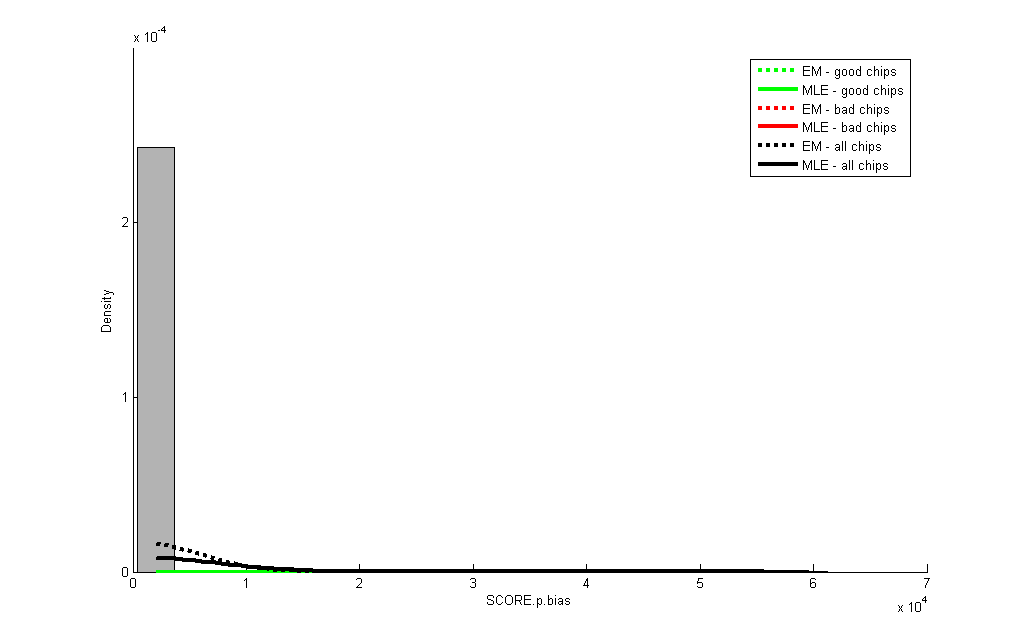

Supplement: Additional file 5 — – SourceCode. Zipped archive contains Matlab source code used for the analyses described in this paper. See the file "READ_ME.txt" for instructions explaining how to run the code. [file 1471-2105-10-191-S5.zip › Output/Distributions - 3-prime scores/SCORE.p.bias_Fig1.tif]

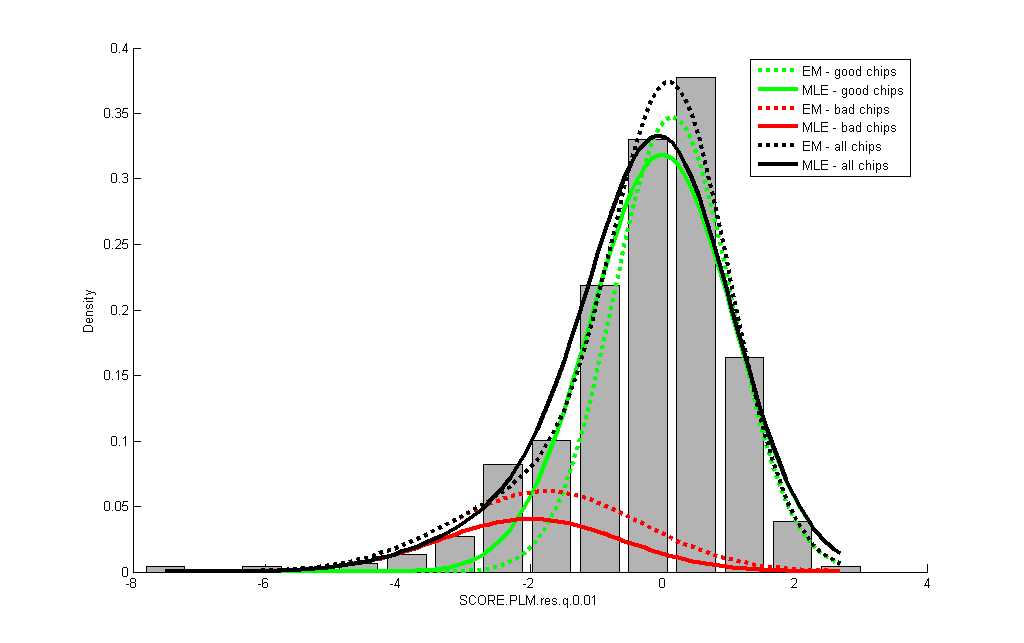

Supplement: Additional file 5 — – SourceCode. Zipped archive contains Matlab source code used for the analyses described in this paper. See the file "READ_ME.txt" for instructions explaining how to run the code. [file 1471-2105-10-191-S5.zip › Output/Distributions - 3-prime scores/SCORE.PLM.res.q.0.01_Fig1.tif]

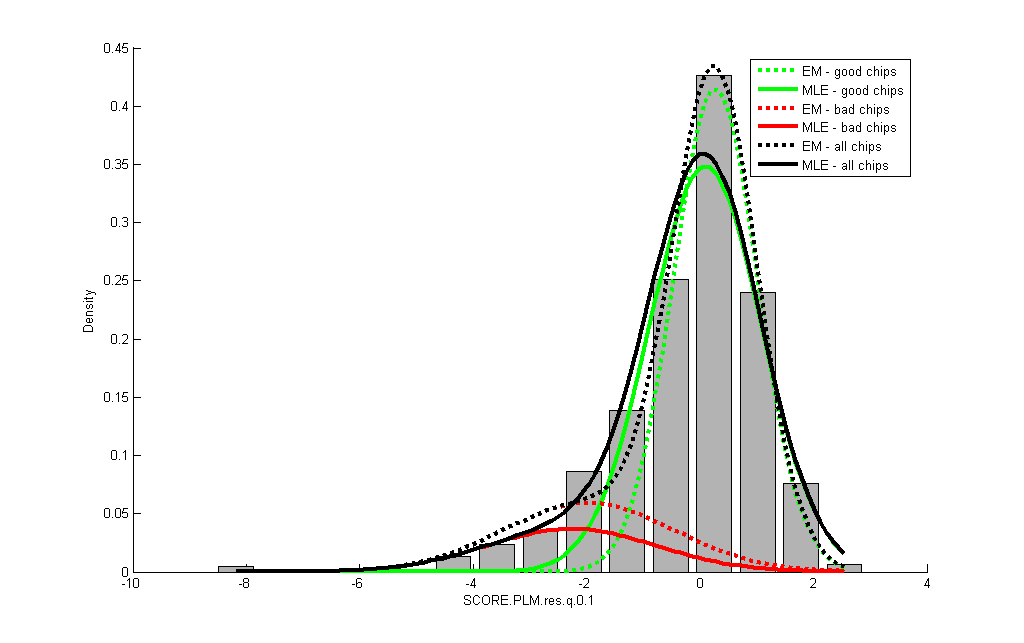

Supplement: Additional file 5 — – SourceCode. Zipped archive contains Matlab source code used for the analyses described in this paper. See the file "READ_ME.txt" for instructions explaining how to run the code. [file 1471-2105-10-191-S5.zip › Output/Distributions - 3-prime scores/SCORE.PLM.res.q.0.1_Fig1.tif]

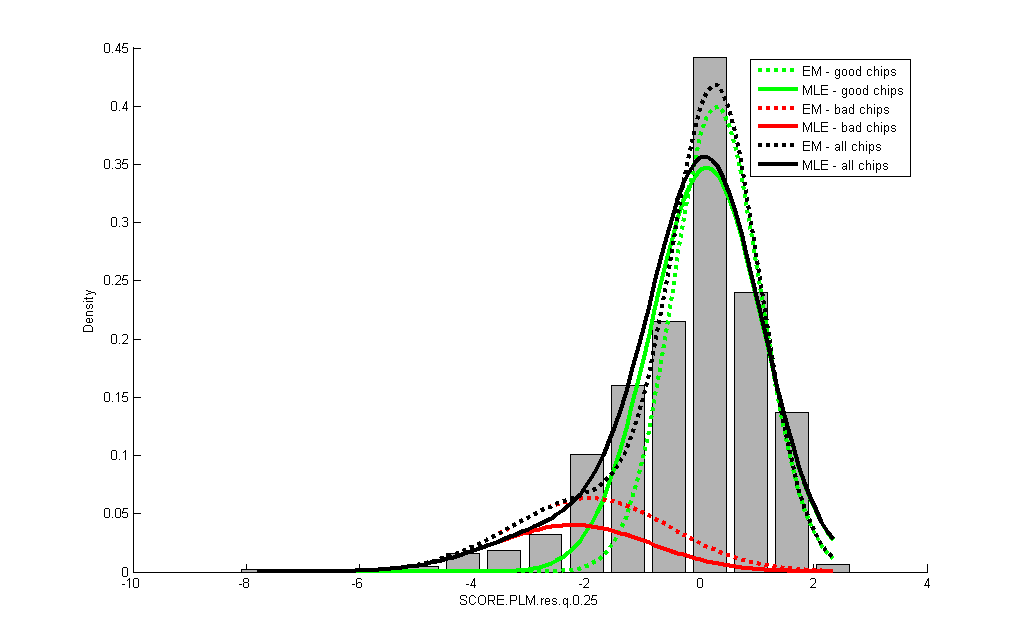

Supplement: Additional file 5 — – SourceCode. Zipped archive contains Matlab source code used for the analyses described in this paper. See the file "READ_ME.txt" for instructions explaining how to run the code. [file 1471-2105-10-191-S5.zip › Output/Distributions - 3-prime scores/SCORE.PLM.res.q.0.25_Fig1.tif]

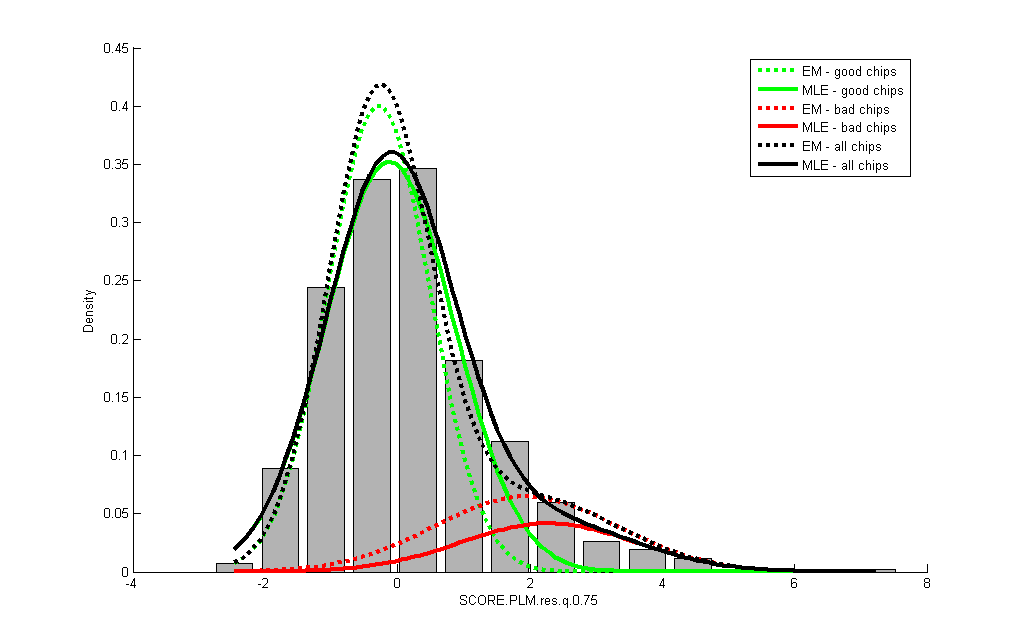

Supplement: Additional file 5 — – SourceCode. Zipped archive contains Matlab source code used for the analyses described in this paper. See the file "READ_ME.txt" for instructions explaining how to run the code. [file 1471-2105-10-191-S5.zip › Output/Distributions - 3-prime scores/SCORE.PLM.res.q.0.75_Fig1.tif]

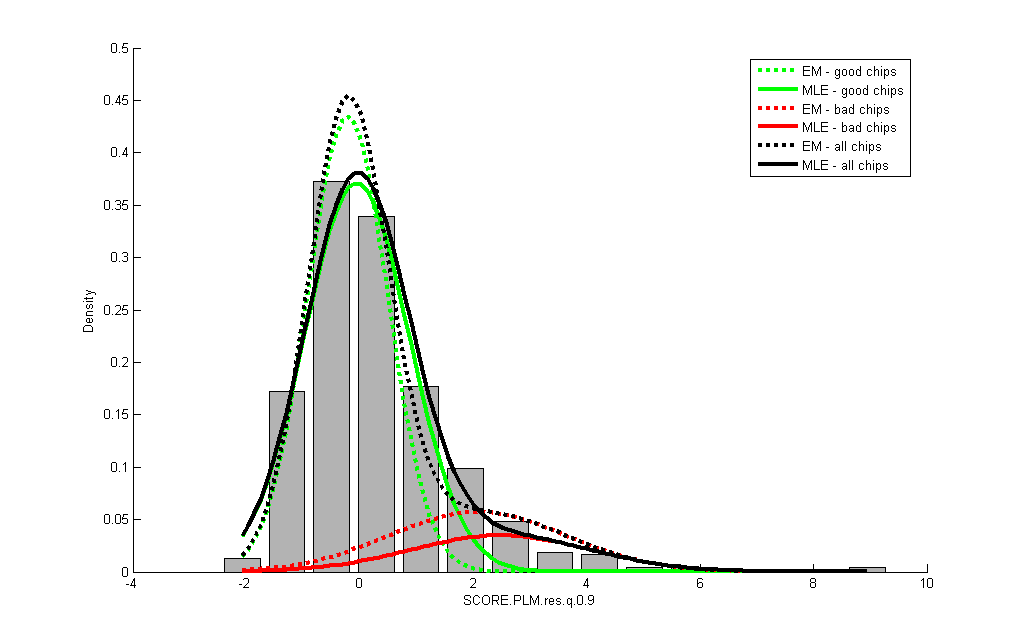

Supplement: Additional file 5 — – SourceCode. Zipped archive contains Matlab source code used for the analyses described in this paper. See the file "READ_ME.txt" for instructions explaining how to run the code. [file 1471-2105-10-191-S5.zip › Output/Distributions - 3-prime scores/SCORE.PLM.res.q.0.9_Fig1.tif]

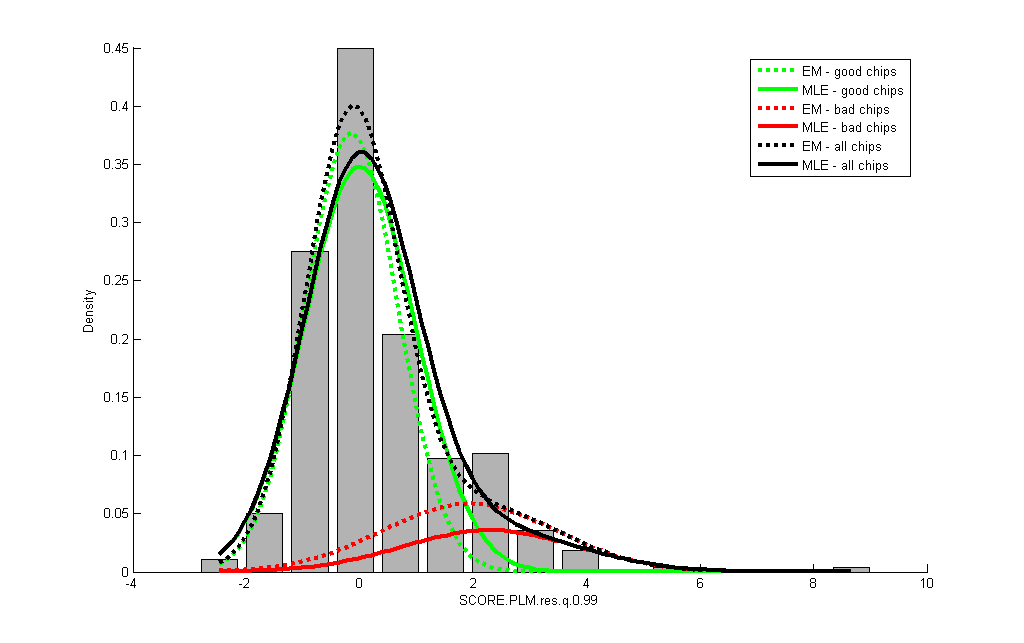

Supplement: Additional file 5 — – SourceCode. Zipped archive contains Matlab source code used for the analyses described in this paper. See the file "READ_ME.txt" for instructions explaining how to run the code. [file 1471-2105-10-191-S5.zip › Output/Distributions - 3-prime scores/SCORE.PLM.res.q.0.99_Fig1.tif]

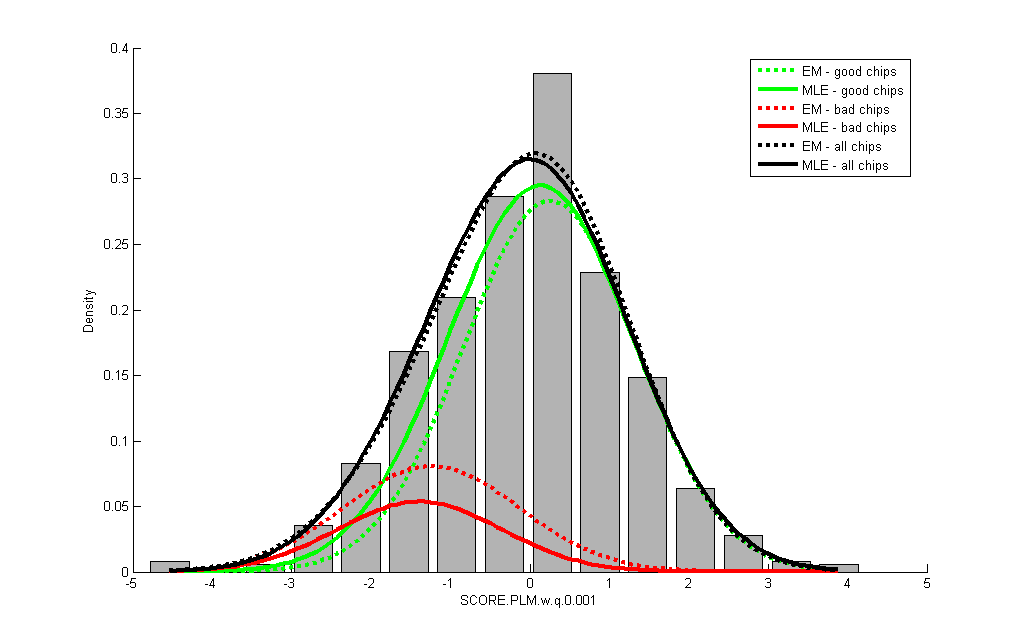

Supplement: Additional file 5 — – SourceCode. Zipped archive contains Matlab source code used for the analyses described in this paper. See the file "READ_ME.txt" for instructions explaining how to run the code. [file 1471-2105-10-191-S5.zip › Output/Distributions - 3-prime scores/SCORE.PLM.w.q.0.001_Fig1.tif]

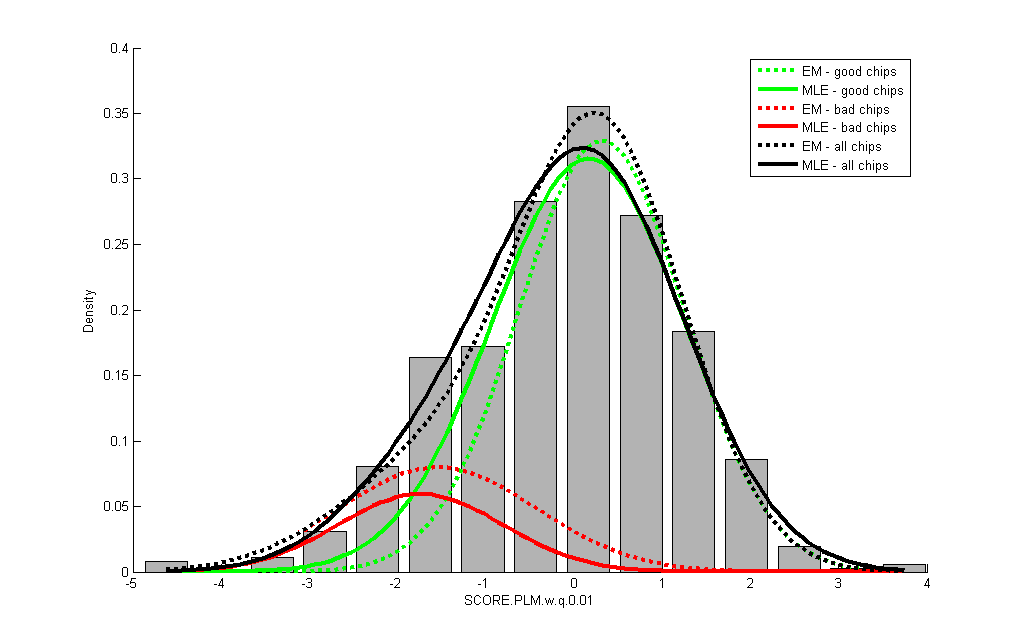

Supplement: Additional file 5 — – SourceCode. Zipped archive contains Matlab source code used for the analyses described in this paper. See the file "READ_ME.txt" for instructions explaining how to run the code. [file 1471-2105-10-191-S5.zip › Output/Distributions - 3-prime scores/SCORE.PLM.w.q.0.01_Fig1.tif]

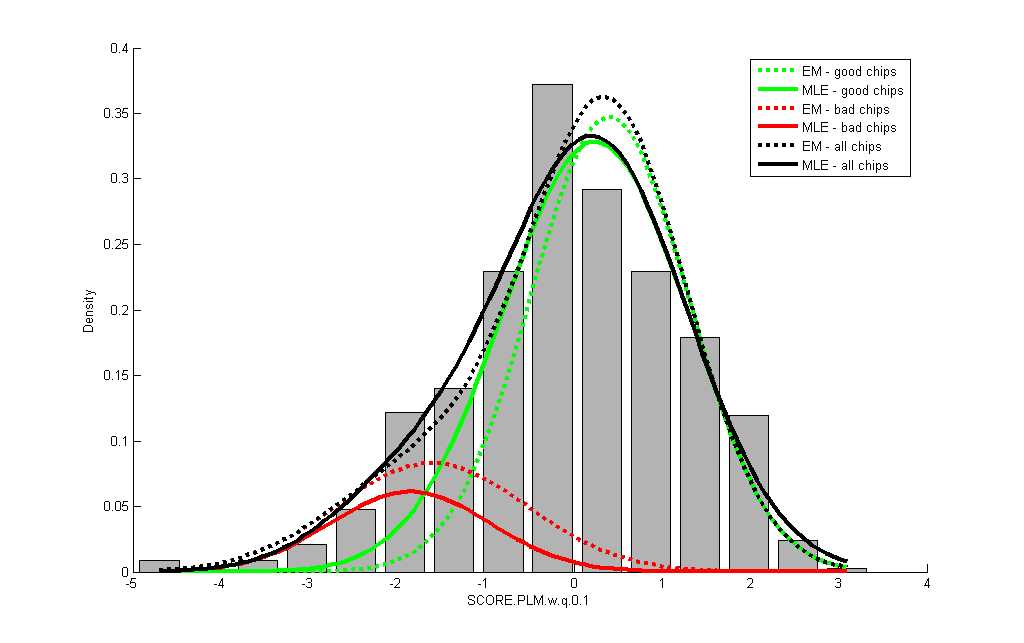

Supplement: Additional file 5 — – SourceCode. Zipped archive contains Matlab source code used for the analyses described in this paper. See the file "READ_ME.txt" for instructions explaining how to run the code. [file 1471-2105-10-191-S5.zip › Output/Distributions - 3-prime scores/SCORE.PLM.w.q.0.1_Fig1.tif]

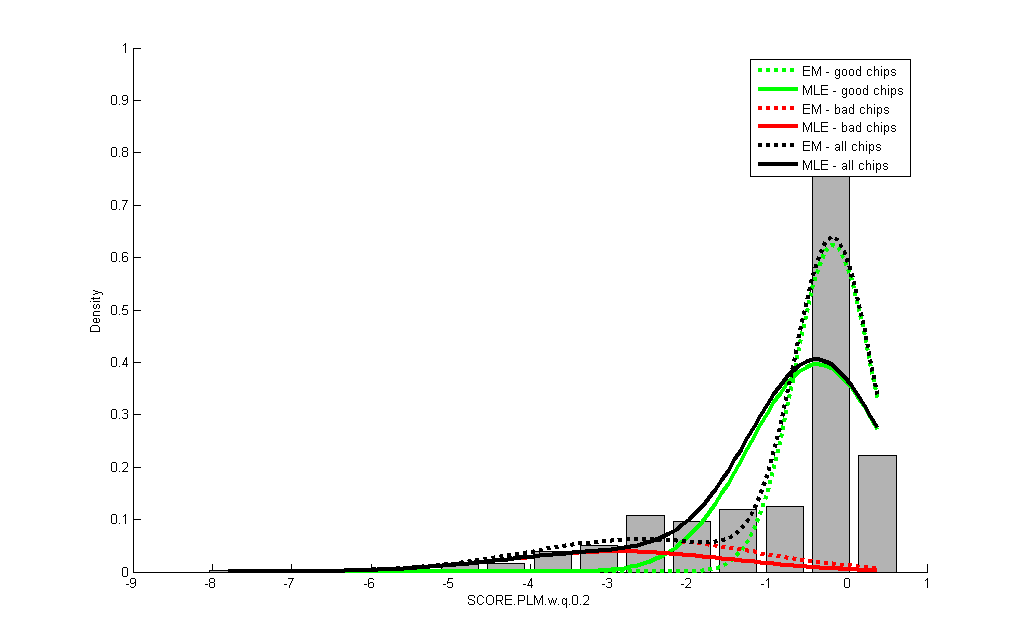

Supplement: Additional file 5 — – SourceCode. Zipped archive contains Matlab source code used for the analyses described in this paper. See the file "READ_ME.txt" for instructions explaining how to run the code. [file 1471-2105-10-191-S5.zip › Output/Distributions - 3-prime scores/SCORE.PLM.w.q.0.2_Fig1.tif]

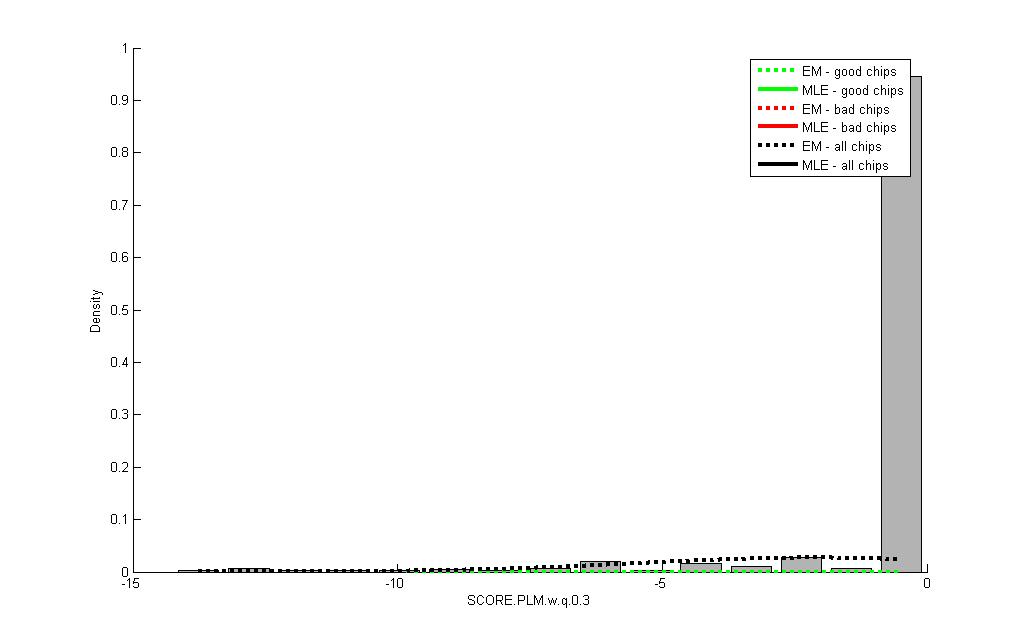

Supplement: Additional file 5 — – SourceCode. Zipped archive contains Matlab source code used for the analyses described in this paper. See the file "READ_ME.txt" for instructions explaining how to run the code. [file 1471-2105-10-191-S5.zip › Output/Distributions - 3-prime scores/SCORE.PLM.w.q.0.3_Fig1.tif]

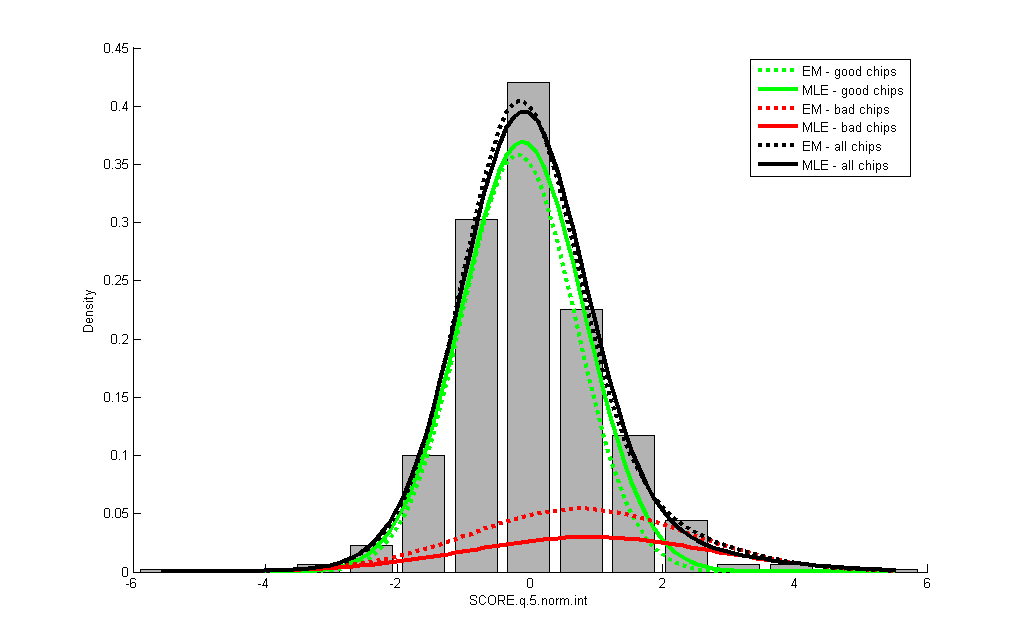

Supplement: Additional file 5 — – SourceCode. Zipped archive contains Matlab source code used for the analyses described in this paper. See the file "READ_ME.txt" for instructions explaining how to run the code. [file 1471-2105-10-191-S5.zip › Output/Distributions - 3-prime scores/SCORE.q.5.norm.int_Fig1.tif]

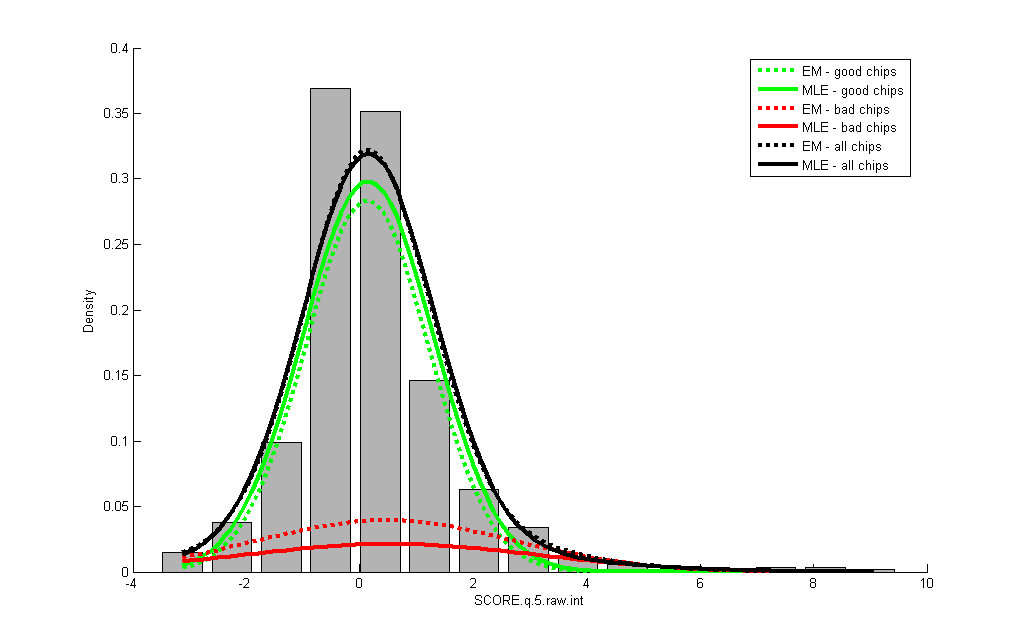

Supplement: Additional file 5 — – SourceCode. Zipped archive contains Matlab source code used for the analyses described in this paper. See the file "READ_ME.txt" for instructions explaining how to run the code. [file 1471-2105-10-191-S5.zip › Output/Distributions - 3-prime scores/SCORE.q.5.raw.int_Fig1.tif]

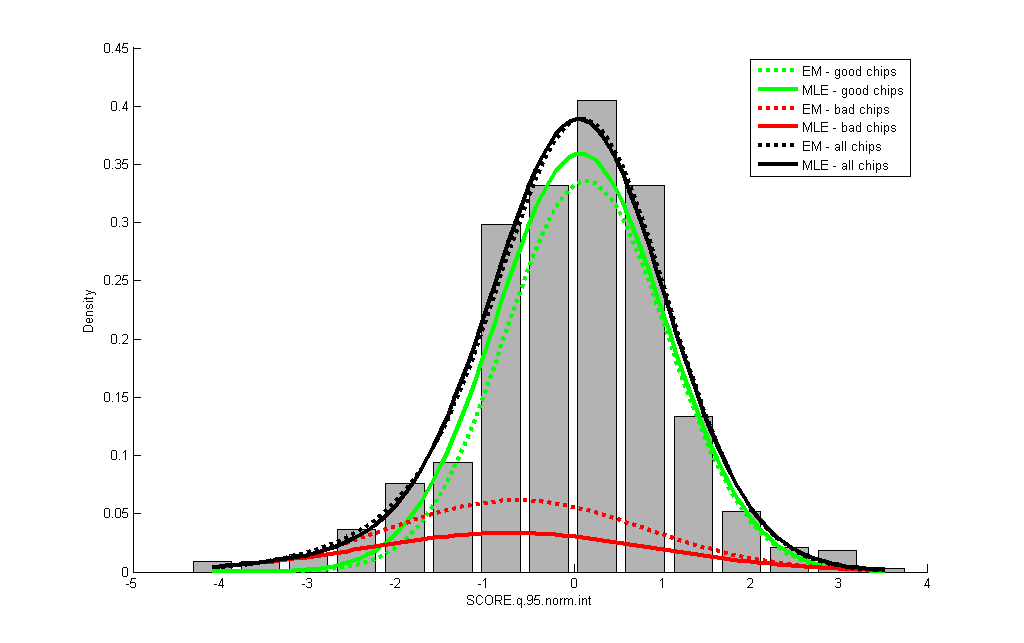

Supplement: Additional file 5 — – SourceCode. Zipped archive contains Matlab source code used for the analyses described in this paper. See the file "READ_ME.txt" for instructions explaining how to run the code. [file 1471-2105-10-191-S5.zip › Output/Distributions - 3-prime scores/SCORE.q.95.norm.int_Fig1.tif]

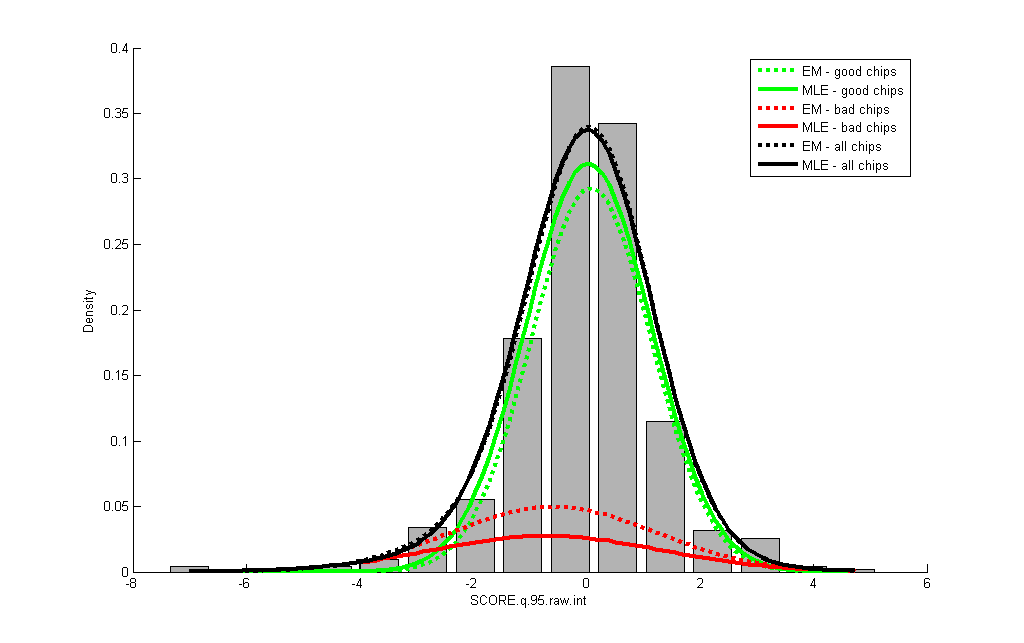

Supplement: Additional file 5 — – SourceCode. Zipped archive contains Matlab source code used for the analyses described in this paper. See the file "READ_ME.txt" for instructions explaining how to run the code. [file 1471-2105-10-191-S5.zip › Output/Distributions - 3-prime scores/SCORE.q.95.raw.int_Fig1.tif]

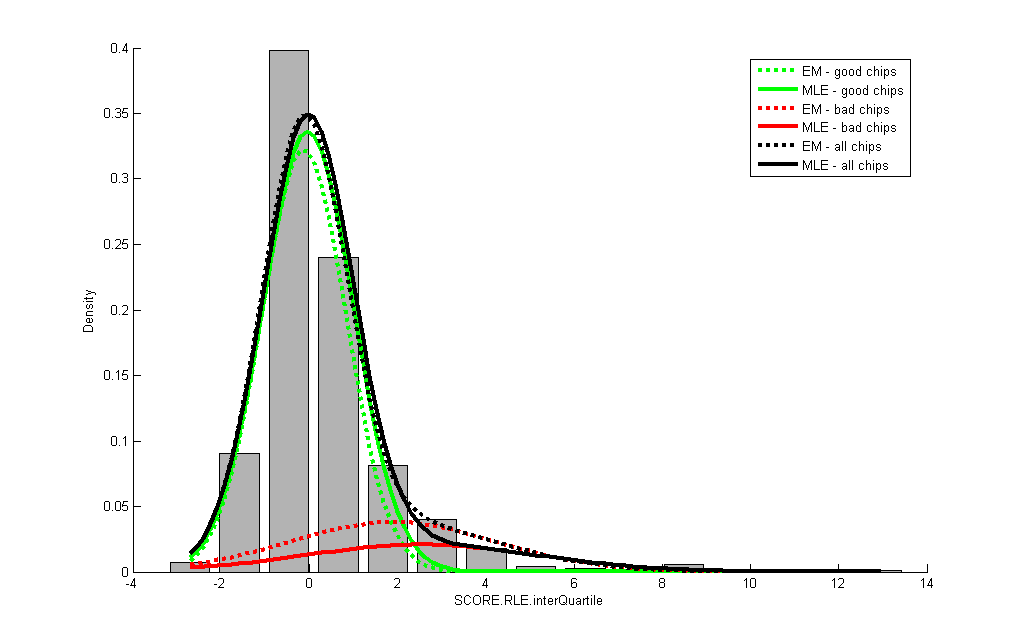

Supplement: Additional file 5 — – SourceCode. Zipped archive contains Matlab source code used for the analyses described in this paper. See the file "READ_ME.txt" for instructions explaining how to run the code. [file 1471-2105-10-191-S5.zip › Output/Distributions - 3-prime scores/SCORE.RLE.interQuartile_Fig1.tif]

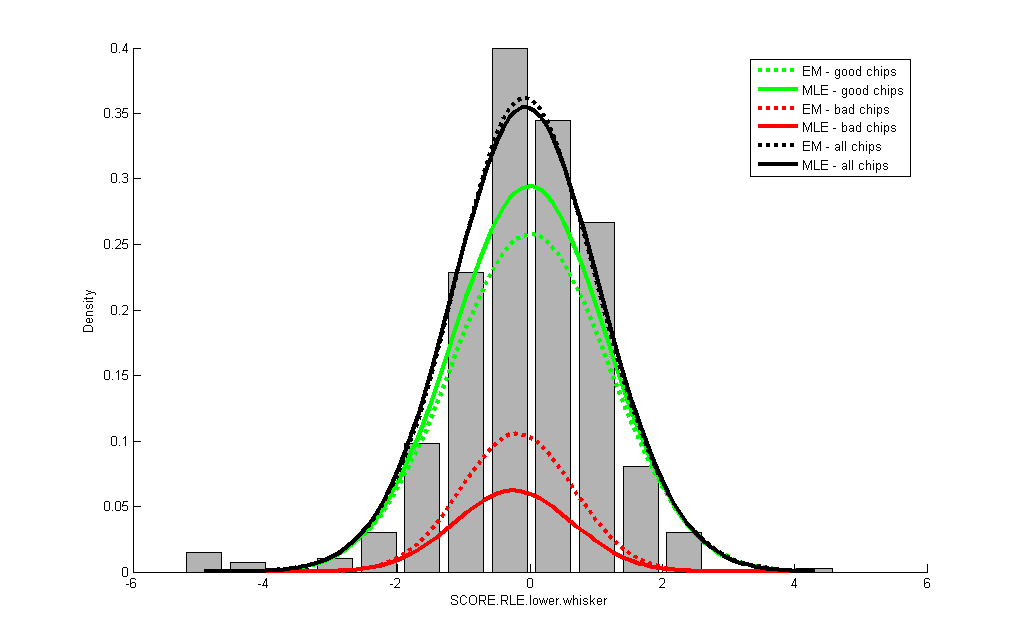

Supplement: Additional file 5 — – SourceCode. Zipped archive contains Matlab source code used for the analyses described in this paper. See the file "READ_ME.txt" for instructions explaining how to run the code. [file 1471-2105-10-191-S5.zip › Output/Distributions - 3-prime scores/SCORE.RLE.lower.whisker_Fig1.tif]

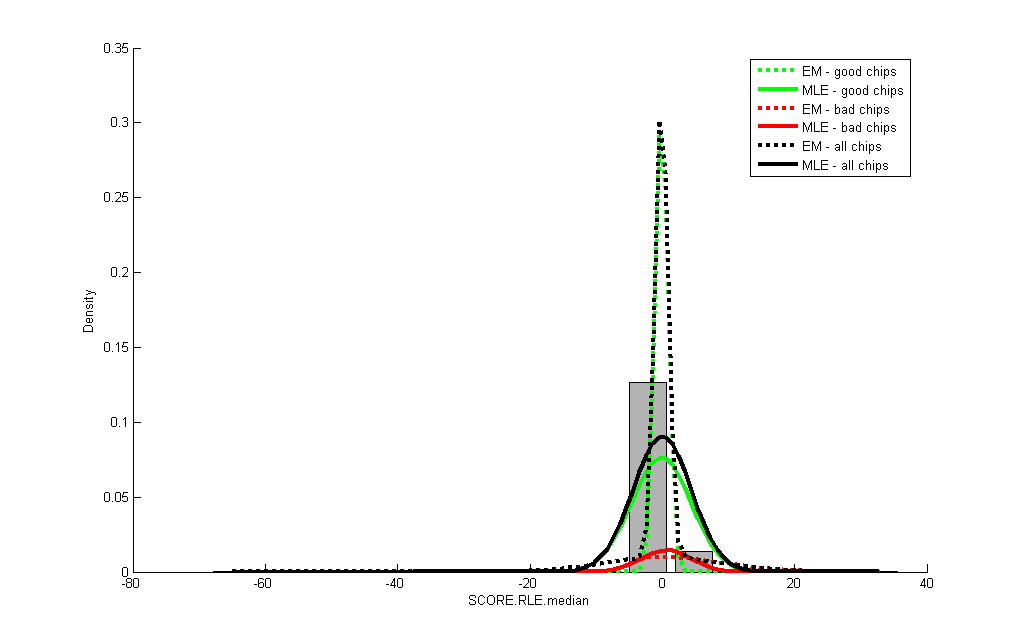

Supplement: Additional file 5 — – SourceCode. Zipped archive contains Matlab source code used for the analyses described in this paper. See the file "READ_ME.txt" for instructions explaining how to run the code. [file 1471-2105-10-191-S5.zip › Output/Distributions - 3-prime scores/SCORE.RLE.median_Fig1.tif]

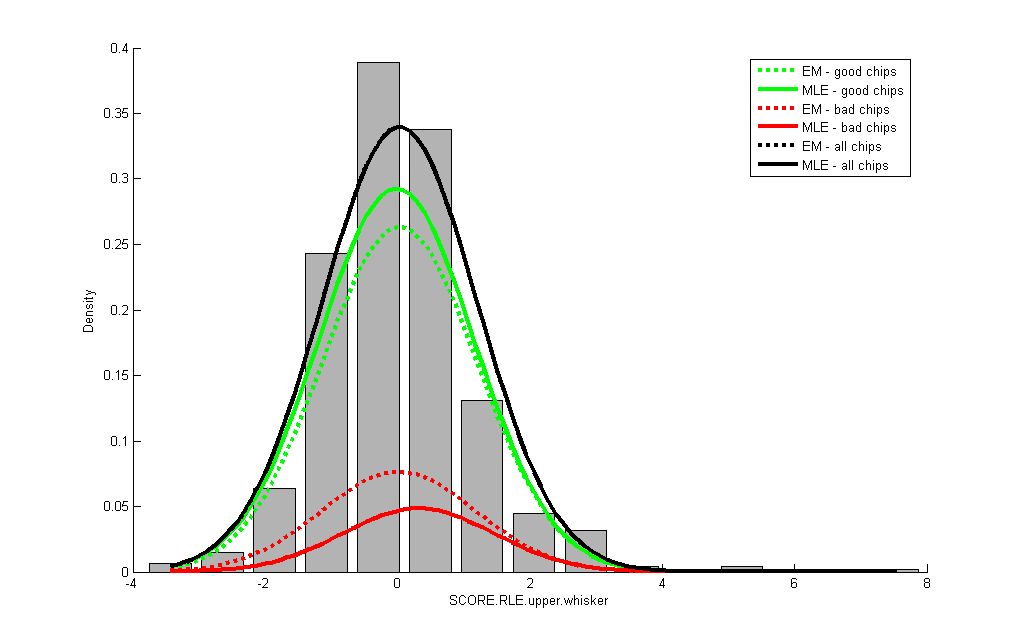

Supplement: Additional file 5 — – SourceCode. Zipped archive contains Matlab source code used for the analyses described in this paper. See the file "READ_ME.txt" for instructions explaining how to run the code. [file 1471-2105-10-191-S5.zip › Output/Distributions - 3-prime scores/SCORE.RLE.upper.whisker_Fig1.tif]

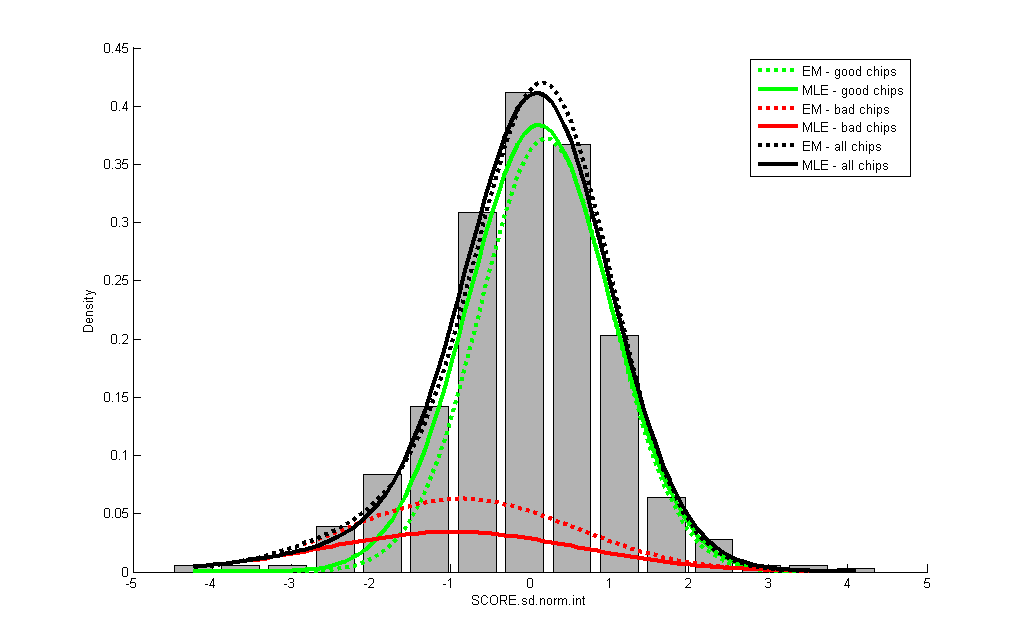

Supplement: Additional file 5 — – SourceCode. Zipped archive contains Matlab source code used for the analyses described in this paper. See the file "READ_ME.txt" for instructions explaining how to run the code. [file 1471-2105-10-191-S5.zip › Output/Distributions - 3-prime scores/SCORE.sd.norm.int_Fig1.tif]

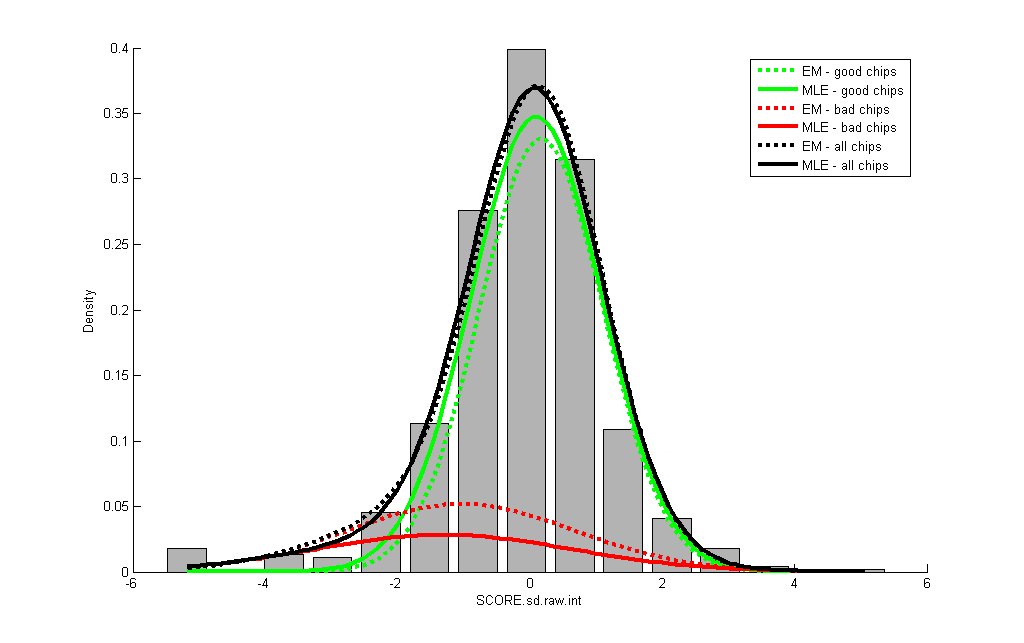

Supplement: Additional file 5 — – SourceCode. Zipped archive contains Matlab source code used for the analyses described in this paper. See the file "READ_ME.txt" for instructions explaining how to run the code. [file 1471-2105-10-191-S5.zip › Output/Distributions - 3-prime scores/SCORE.sd.raw.int_Fig1.tif]

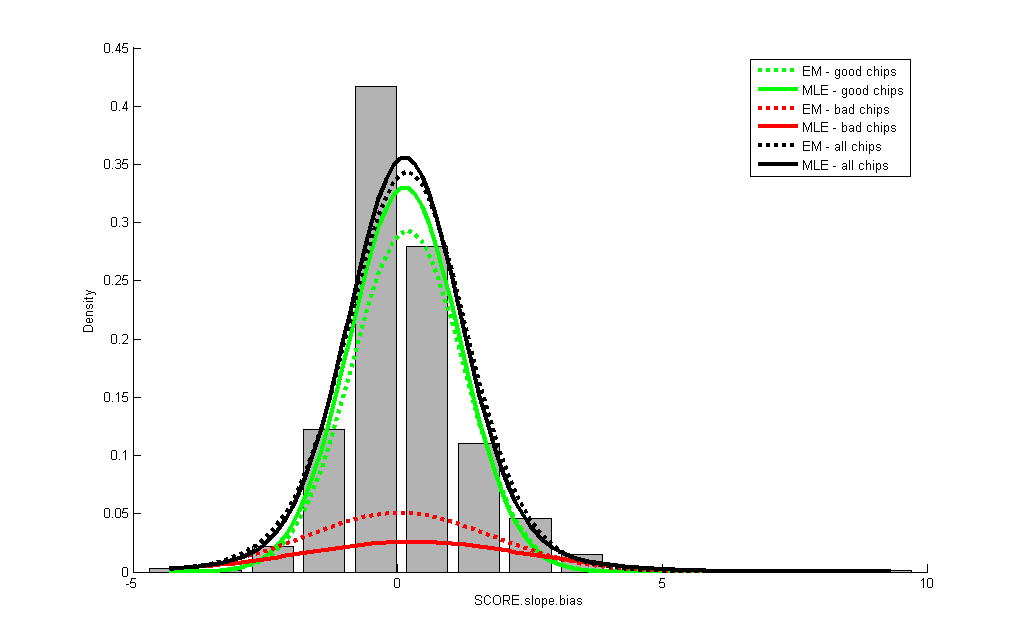

Supplement: Additional file 5 — – SourceCode. Zipped archive contains Matlab source code used for the analyses described in this paper. See the file "READ_ME.txt" for instructions explaining how to run the code. [file 1471-2105-10-191-S5.zip › Output/Distributions - 3-prime scores/SCORE.slope.bias_Fig1.tif]

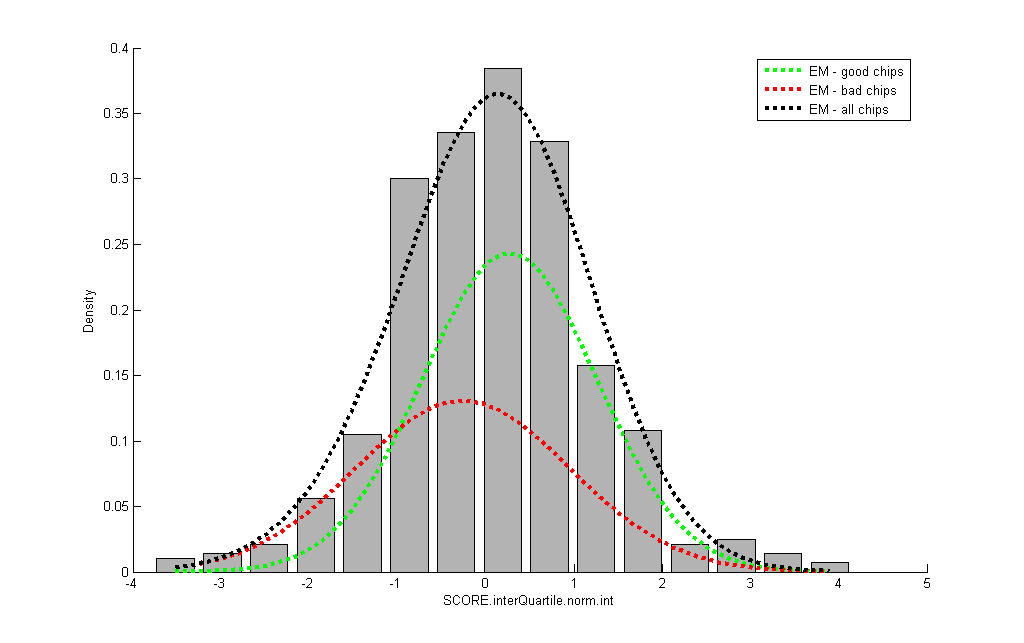

Supplement: Additional file 5 — – SourceCode. Zipped archive contains Matlab source code used for the analyses described in this paper. See the file "READ_ME.txt" for instructions explaining how to run the code. [file 1471-2105-10-191-S5.zip › Output/Distributions - exon BioC/SCORE.interQuartile.norm.int_Fig1.tif]

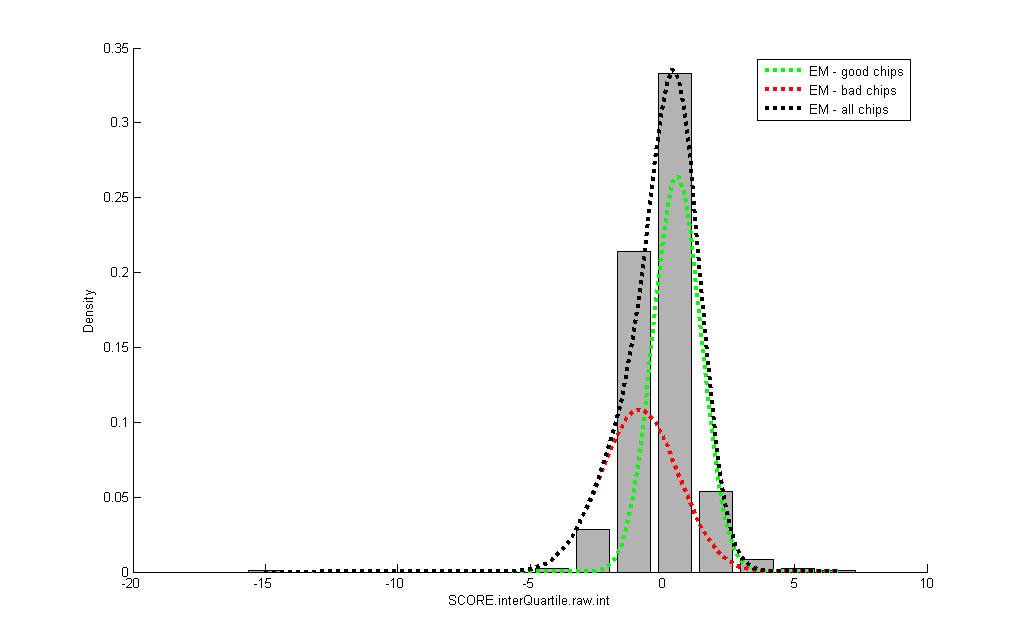

Supplement: Additional file 5 — – SourceCode. Zipped archive contains Matlab source code used for the analyses described in this paper. See the file "READ_ME.txt" for instructions explaining how to run the code. [file 1471-2105-10-191-S5.zip › Output/Distributions - exon BioC/SCORE.interQuartile.raw.int_Fig1.tif]

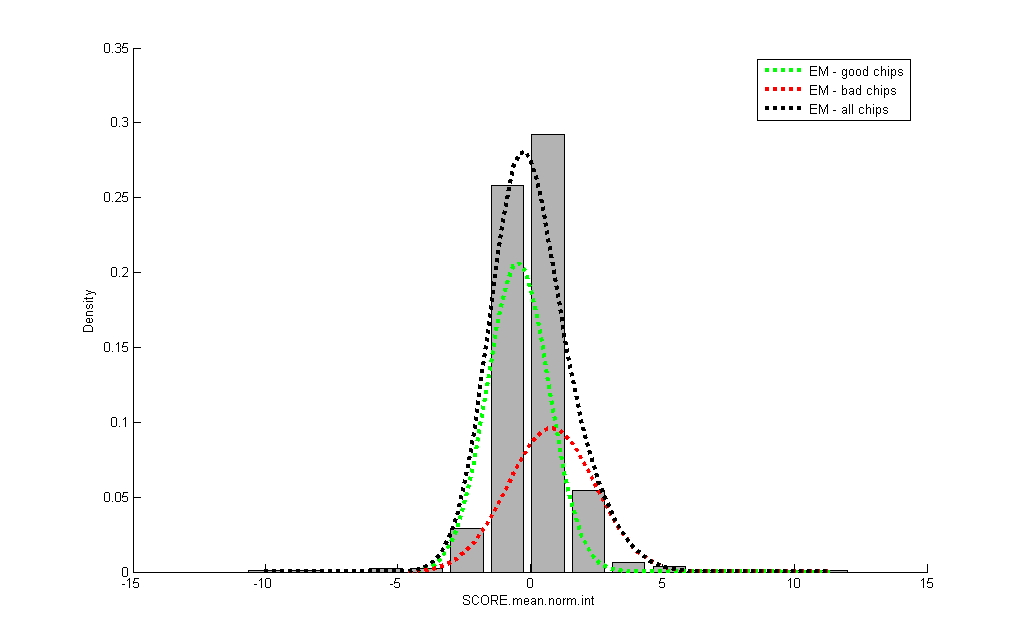

Supplement: Additional file 5 — – SourceCode. Zipped archive contains Matlab source code used for the analyses described in this paper. See the file "READ_ME.txt" for instructions explaining how to run the code. [file 1471-2105-10-191-S5.zip › Output/Distributions - exon BioC/SCORE.mean.norm.int_Fig1.tif]

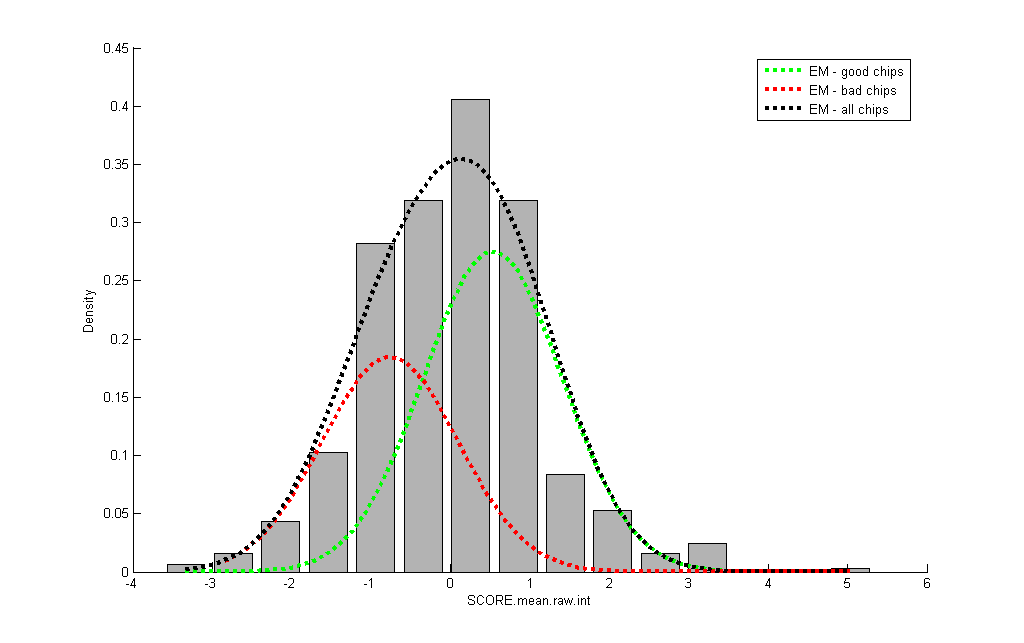

Supplement: Additional file 5 — – SourceCode. Zipped archive contains Matlab source code used for the analyses described in this paper. See the file "READ_ME.txt" for instructions explaining how to run the code. [file 1471-2105-10-191-S5.zip › Output/Distributions - exon BioC/SCORE.mean.raw.int_Fig1.tif]

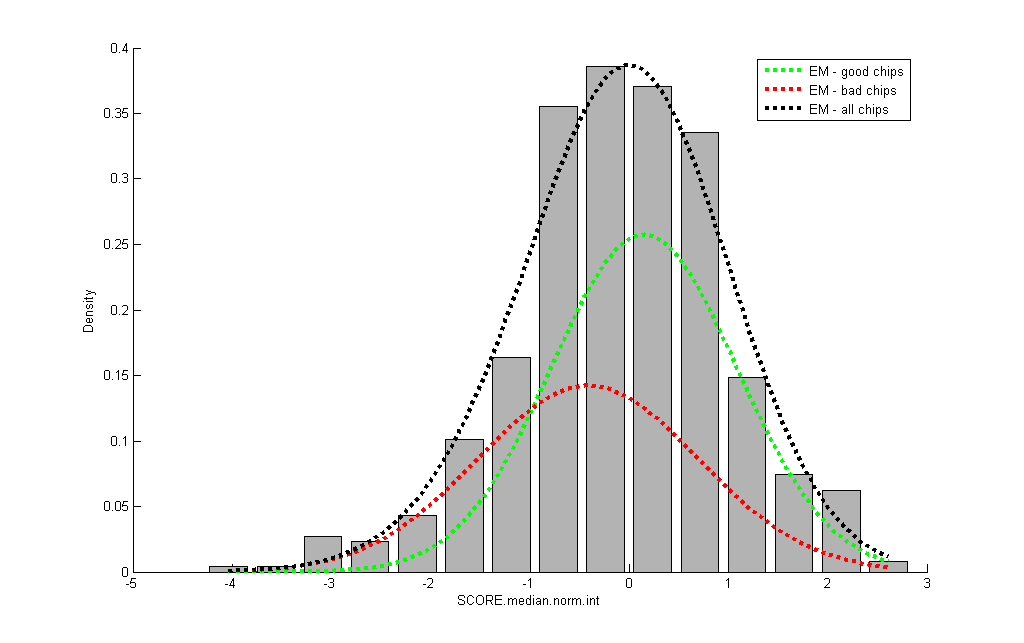

Supplement: Additional file 5 — – SourceCode. Zipped archive contains Matlab source code used for the analyses described in this paper. See the file "READ_ME.txt" for instructions explaining how to run the code. [file 1471-2105-10-191-S5.zip › Output/Distributions - exon BioC/SCORE.median.norm.int_Fig1.tif]

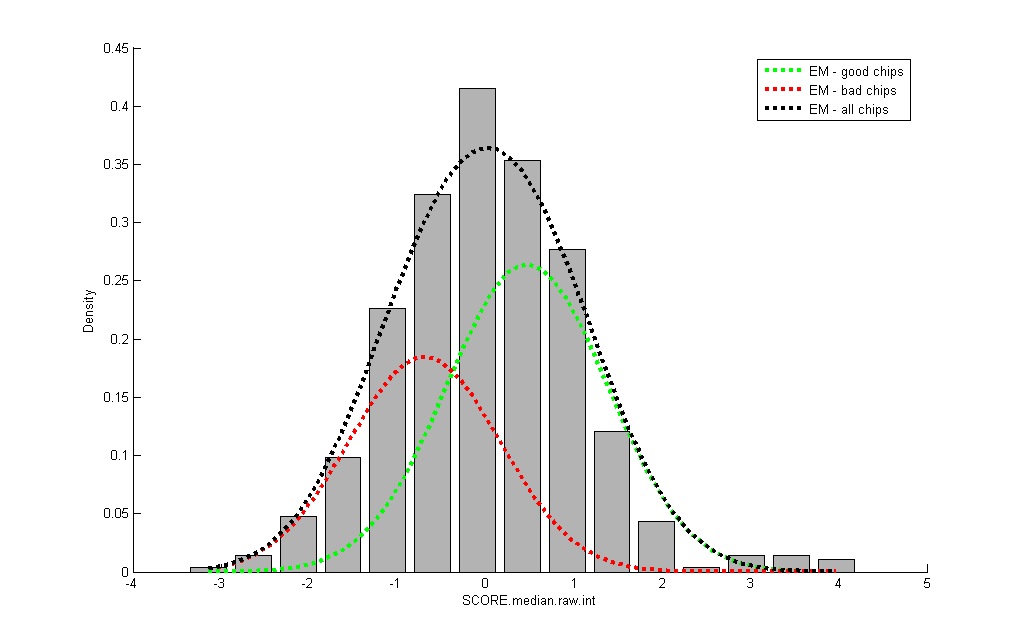

Supplement: Additional file 5 — – SourceCode. Zipped archive contains Matlab source code used for the analyses described in this paper. See the file "READ_ME.txt" for instructions explaining how to run the code. [file 1471-2105-10-191-S5.zip › Output/Distributions - exon BioC/SCORE.median.raw.int_Fig1.tif]

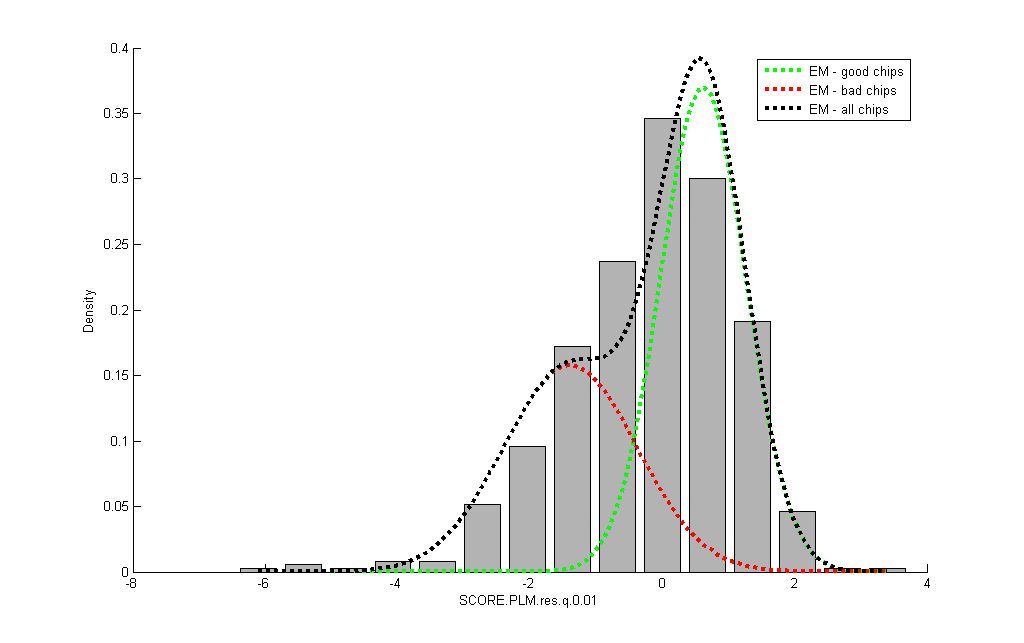

Supplement: Additional file 5 — – SourceCode. Zipped archive contains Matlab source code used for the analyses described in this paper. See the file "READ_ME.txt" for instructions explaining how to run the code. [file 1471-2105-10-191-S5.zip › Output/Distributions - exon BioC/SCORE.PLM.res.q.0.01_Fig1.tif]

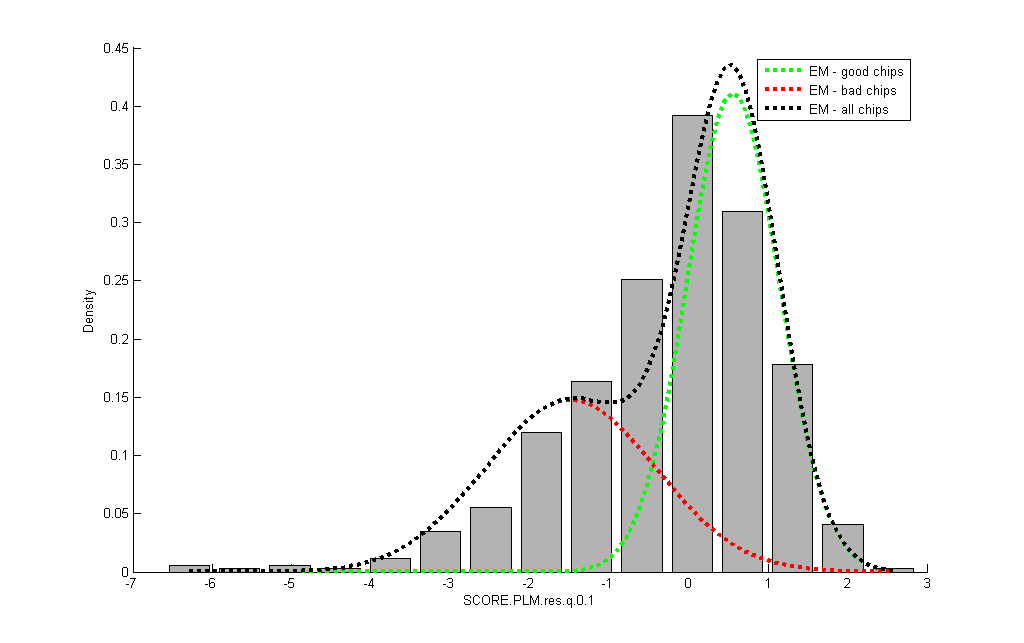

Supplement: Additional file 5 — – SourceCode. Zipped archive contains Matlab source code used for the analyses described in this paper. See the file "READ_ME.txt" for instructions explaining how to run the code. [file 1471-2105-10-191-S5.zip › Output/Distributions - exon BioC/SCORE.PLM.res.q.0.1_Fig1.tif]

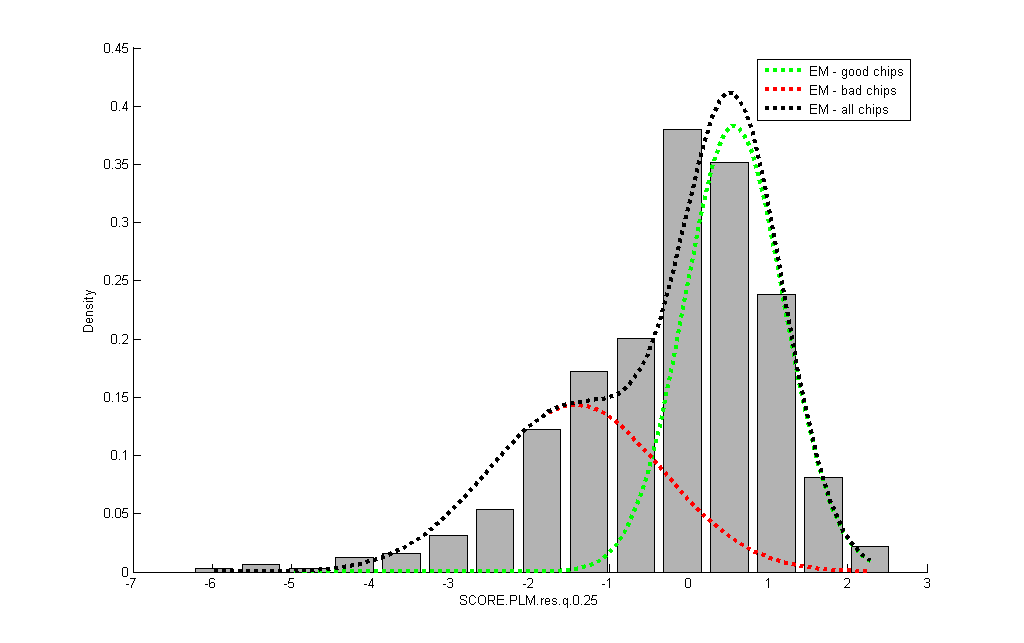

Supplement: Additional file 5 — – SourceCode. Zipped archive contains Matlab source code used for the analyses described in this paper. See the file "READ_ME.txt" for instructions explaining how to run the code. [file 1471-2105-10-191-S5.zip › Output/Distributions - exon BioC/SCORE.PLM.res.q.0.25_Fig1.tif]

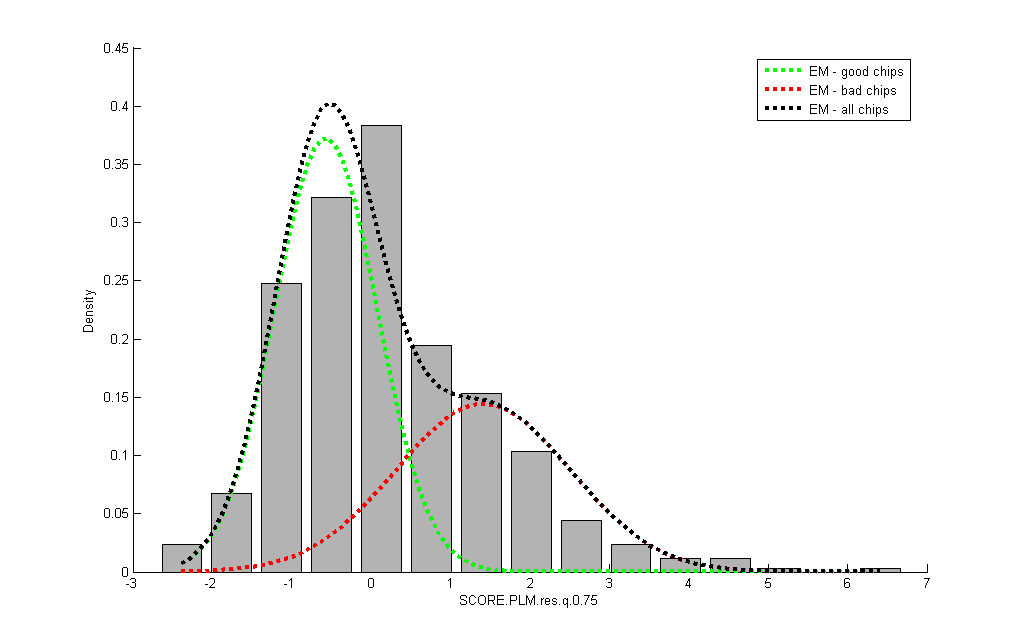

Supplement: Additional file 5 — – SourceCode. Zipped archive contains Matlab source code used for the analyses described in this paper. See the file "READ_ME.txt" for instructions explaining how to run the code. [file 1471-2105-10-191-S5.zip › Output/Distributions - exon BioC/SCORE.PLM.res.q.0.75_Fig1.tif]

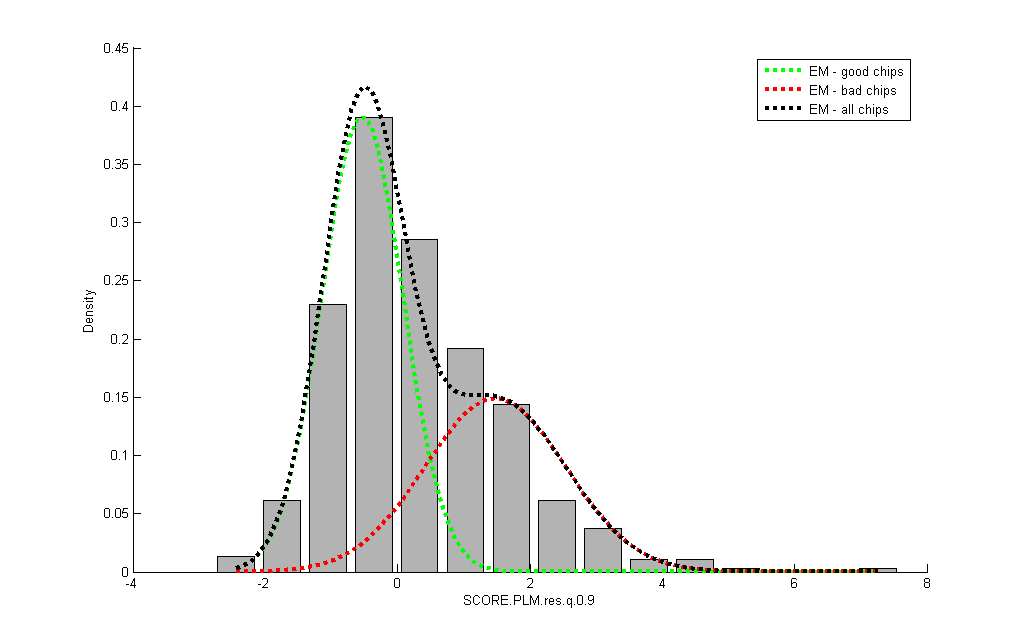

Supplement: Additional file 5 — – SourceCode. Zipped archive contains Matlab source code used for the analyses described in this paper. See the file "READ_ME.txt" for instructions explaining how to run the code. [file 1471-2105-10-191-S5.zip › Output/Distributions - exon BioC/SCORE.PLM.res.q.0.9_Fig1.tif]

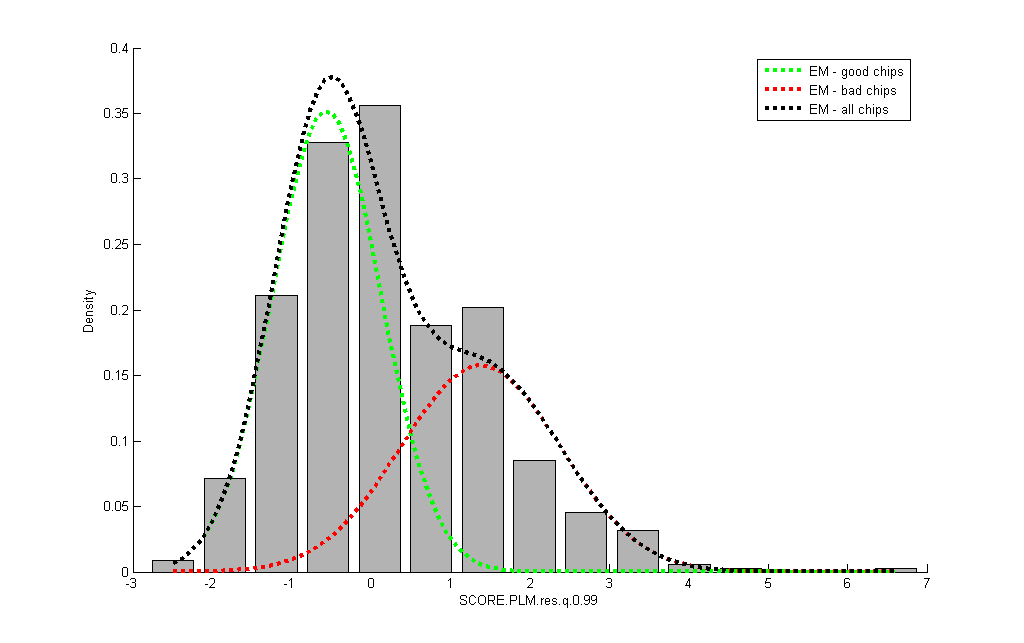

Supplement: Additional file 5 — – SourceCode. Zipped archive contains Matlab source code used for the analyses described in this paper. See the file "READ_ME.txt" for instructions explaining how to run the code. [file 1471-2105-10-191-S5.zip › Output/Distributions - exon BioC/SCORE.PLM.res.q.0.99_Fig1.tif]

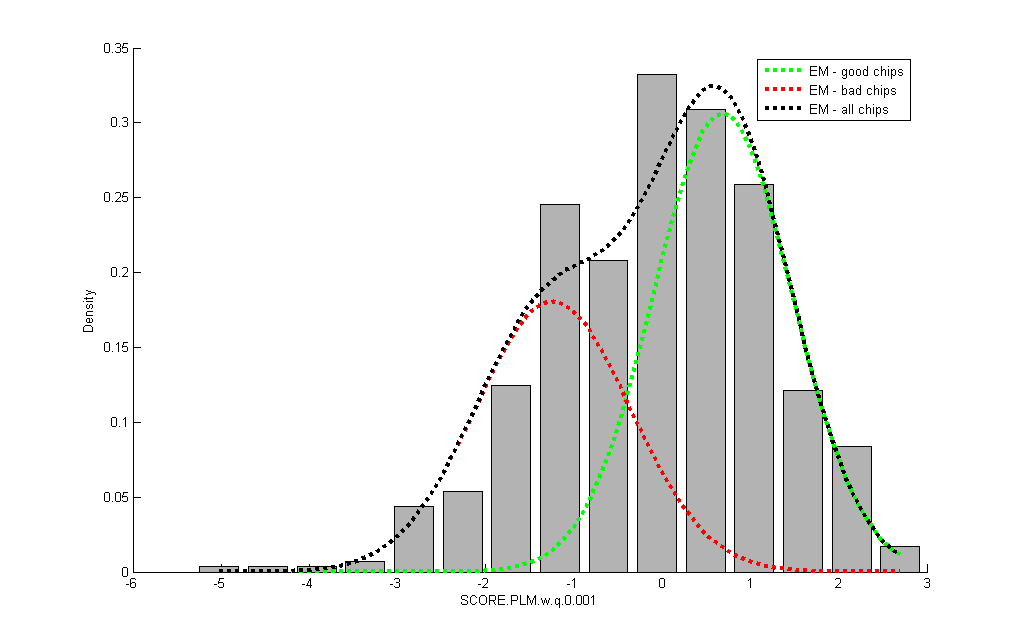

Supplement: Additional file 5 — – SourceCode. Zipped archive contains Matlab source code used for the analyses described in this paper. See the file "READ_ME.txt" for instructions explaining how to run the code. [file 1471-2105-10-191-S5.zip › Output/Distributions - exon BioC/SCORE.PLM.w.q.0.001_Fig1.tif]

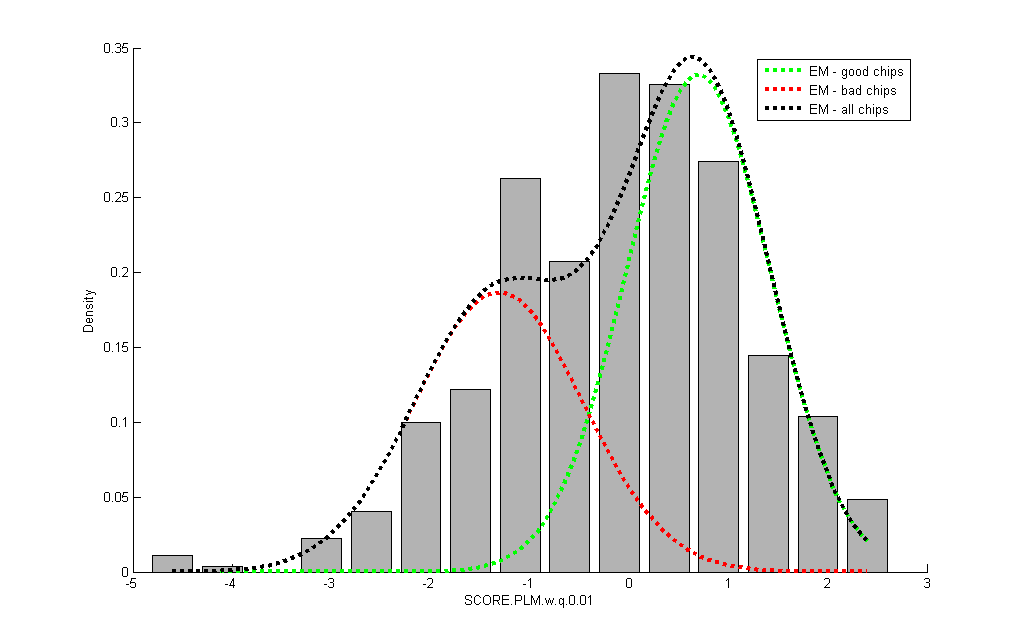

Supplement: Additional file 5 — – SourceCode. Zipped archive contains Matlab source code used for the analyses described in this paper. See the file "READ_ME.txt" for instructions explaining how to run the code. [file 1471-2105-10-191-S5.zip › Output/Distributions - exon BioC/SCORE.PLM.w.q.0.01_Fig1.tif]

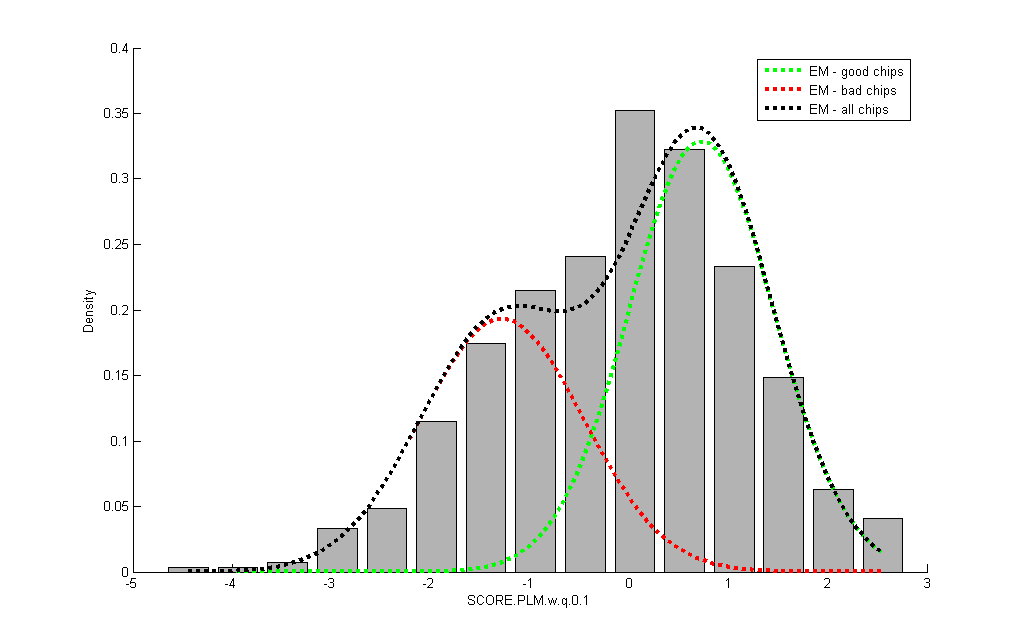

Supplement: Additional file 5 — – SourceCode. Zipped archive contains Matlab source code used for the analyses described in this paper. See the file "READ_ME.txt" for instructions explaining how to run the code. [file 1471-2105-10-191-S5.zip › Output/Distributions - exon BioC/SCORE.PLM.w.q.0.1_Fig1.tif]

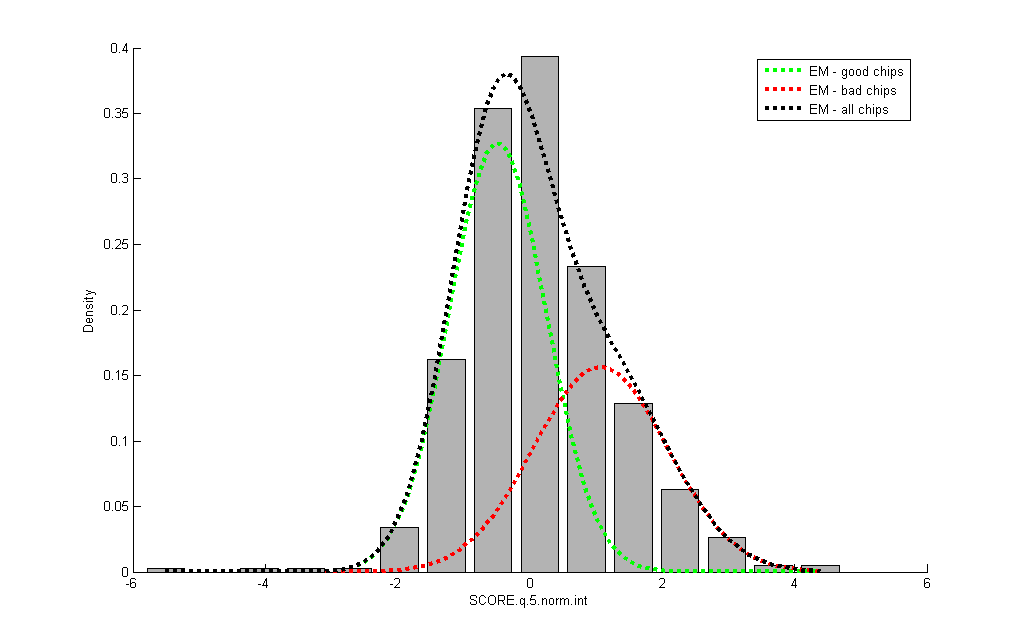

Supplement: Additional file 5 — – SourceCode. Zipped archive contains Matlab source code used for the analyses described in this paper. See the file "READ_ME.txt" for instructions explaining how to run the code. [file 1471-2105-10-191-S5.zip › Output/Distributions - exon BioC/SCORE.q.5.norm.int_Fig1.tif]

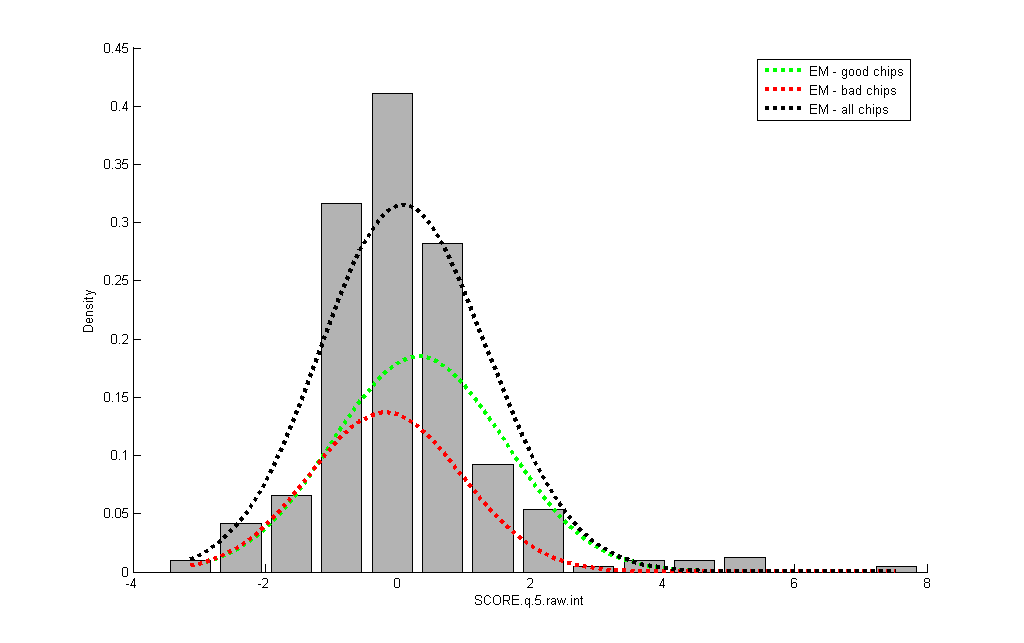

Supplement: Additional file 5 — – SourceCode. Zipped archive contains Matlab source code used for the analyses described in this paper. See the file "READ_ME.txt" for instructions explaining how to run the code. [file 1471-2105-10-191-S5.zip › Output/Distributions - exon BioC/SCORE.q.5.raw.int_Fig1.tif]

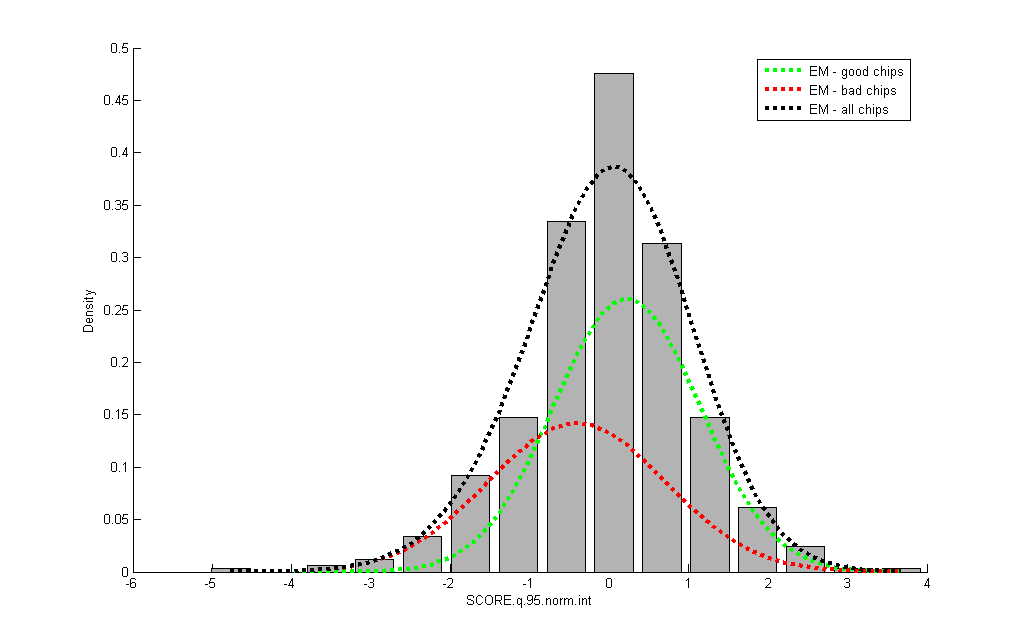

Supplement: Additional file 5 — – SourceCode. Zipped archive contains Matlab source code used for the analyses described in this paper. See the file "READ_ME.txt" for instructions explaining how to run the code. [file 1471-2105-10-191-S5.zip › Output/Distributions - exon BioC/SCORE.q.95.norm.int_Fig1.tif]

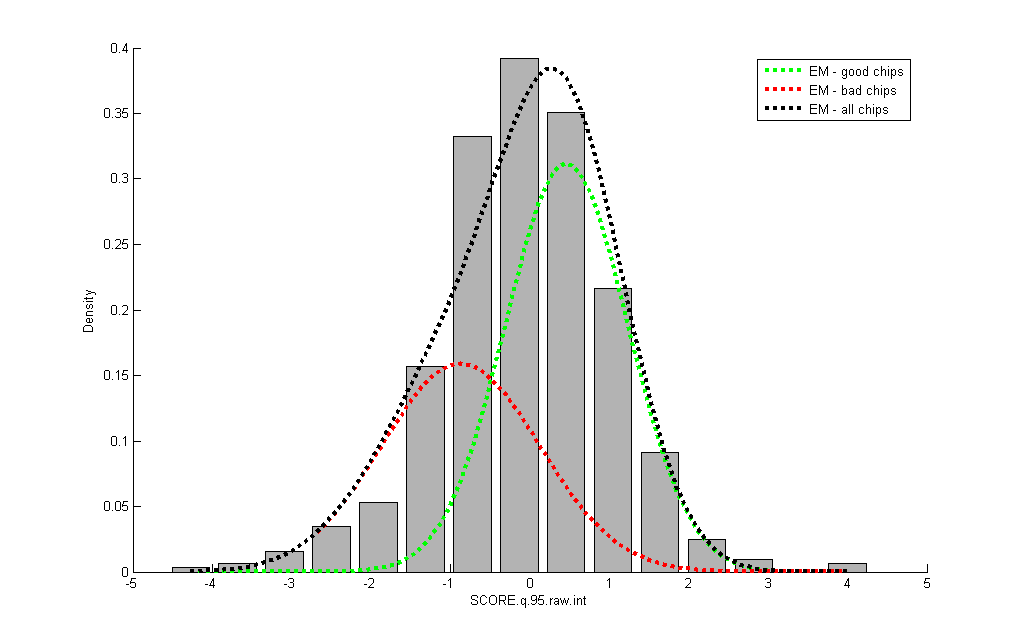

Supplement: Additional file 5 — – SourceCode. Zipped archive contains Matlab source code used for the analyses described in this paper. See the file "READ_ME.txt" for instructions explaining how to run the code. [file 1471-2105-10-191-S5.zip › Output/Distributions - exon BioC/SCORE.q.95.raw.int_Fig1.tif]

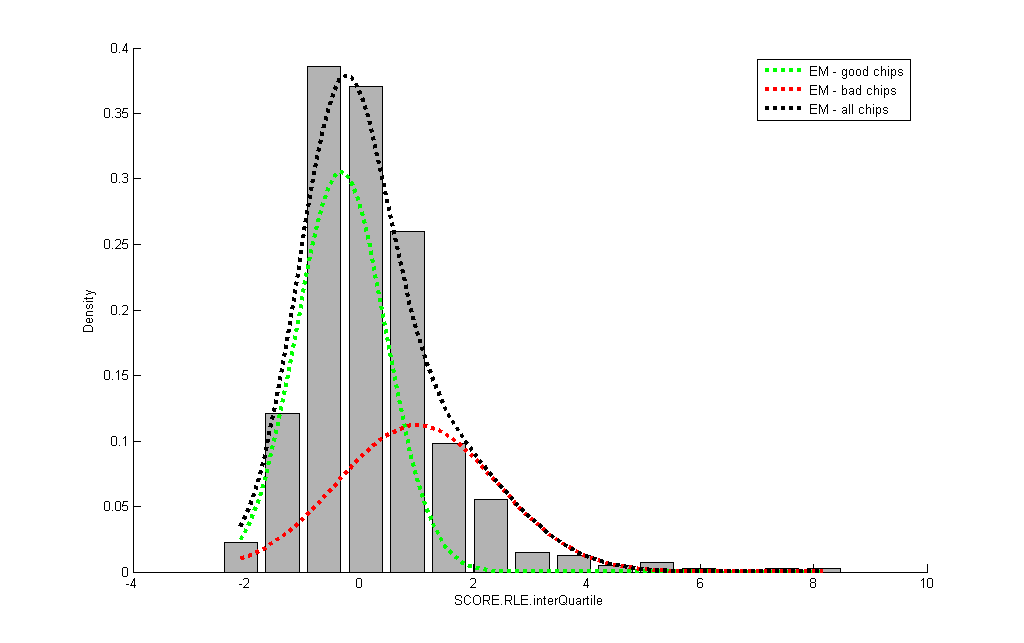

Supplement: Additional file 5 — – SourceCode. Zipped archive contains Matlab source code used for the analyses described in this paper. See the file "READ_ME.txt" for instructions explaining how to run the code. [file 1471-2105-10-191-S5.zip › Output/Distributions - exon BioC/SCORE.RLE.interQuartile_Fig1.tif]

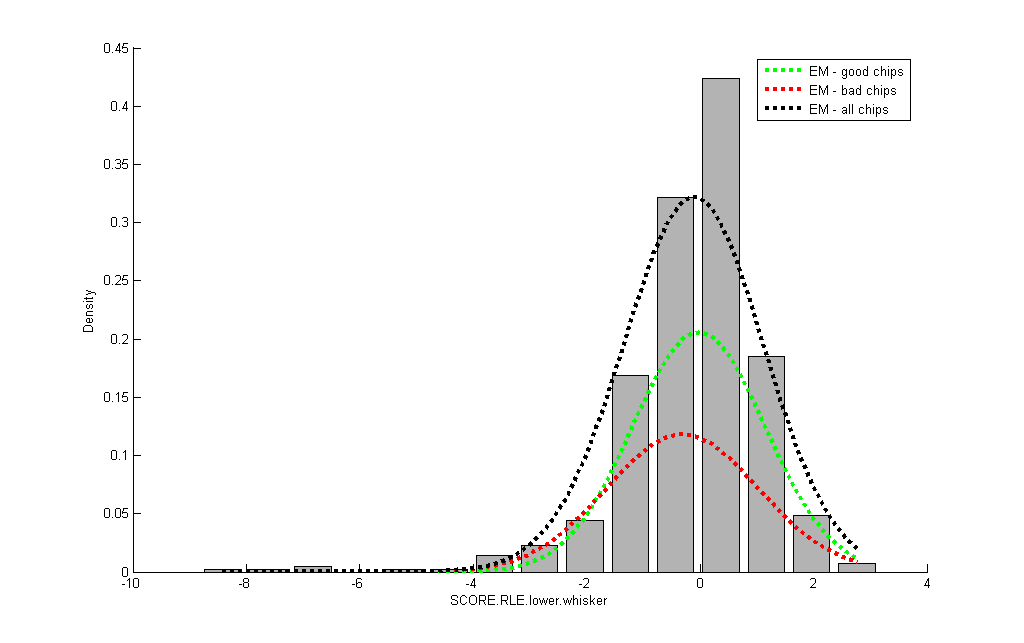

Supplement: Additional file 5 — – SourceCode. Zipped archive contains Matlab source code used for the analyses described in this paper. See the file "READ_ME.txt" for instructions explaining how to run the code. [file 1471-2105-10-191-S5.zip › Output/Distributions - exon BioC/SCORE.RLE.lower.whisker_Fig1.tif]

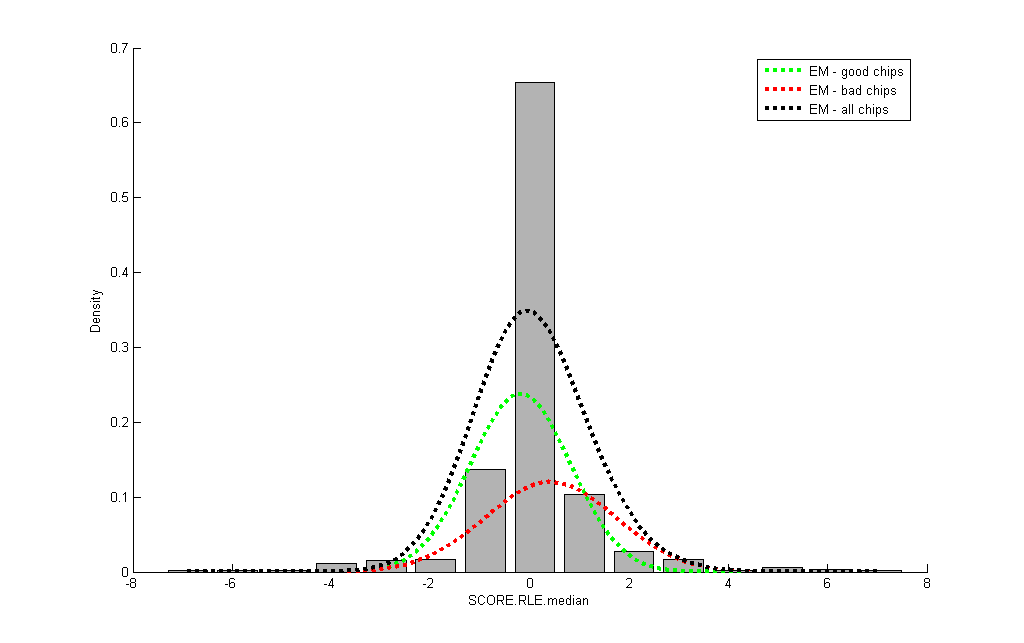

Supplement: Additional file 5 — – SourceCode. Zipped archive contains Matlab source code used for the analyses described in this paper. See the file "READ_ME.txt" for instructions explaining how to run the code. [file 1471-2105-10-191-S5.zip › Output/Distributions - exon BioC/SCORE.RLE.median_Fig1.tif]

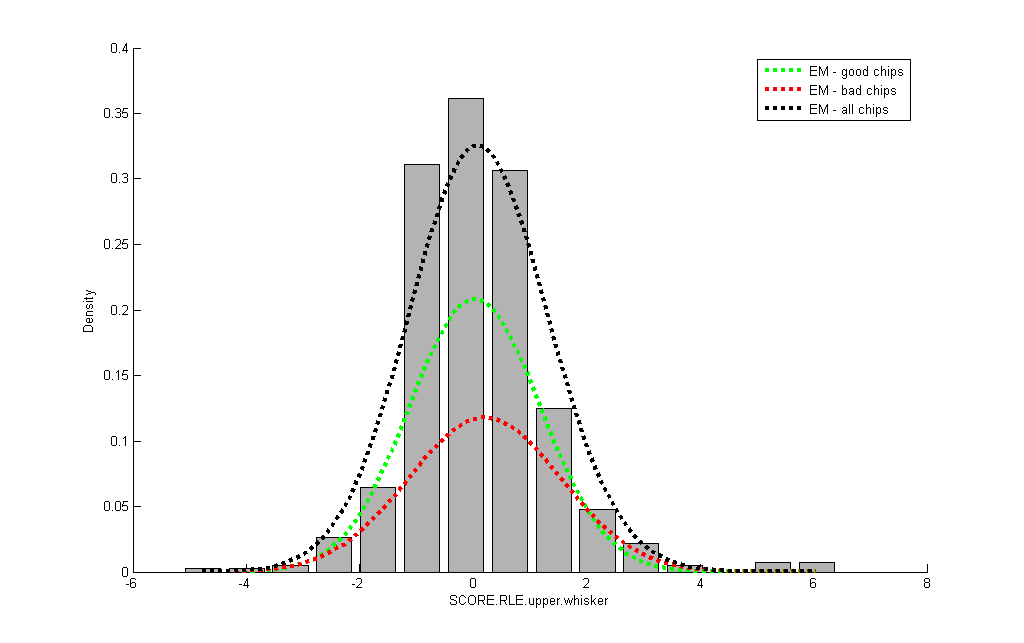

Supplement: Additional file 5 — – SourceCode. Zipped archive contains Matlab source code used for the analyses described in this paper. See the file "READ_ME.txt" for instructions explaining how to run the code. [file 1471-2105-10-191-S5.zip › Output/Distributions - exon BioC/SCORE.RLE.upper.whisker_Fig1.tif]

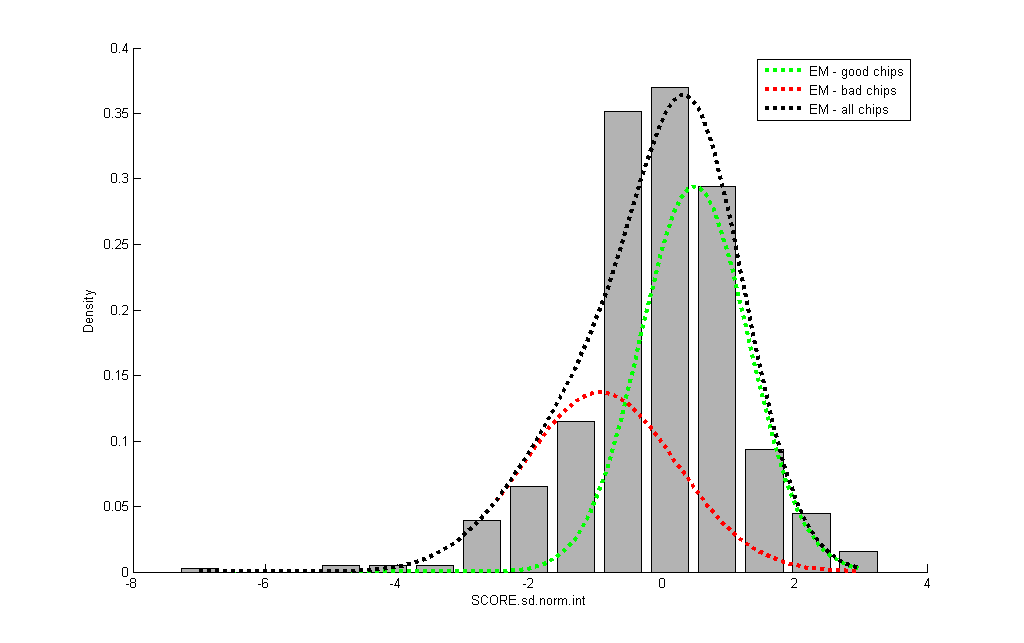

Supplement: Additional file 5 — – SourceCode. Zipped archive contains Matlab source code used for the analyses described in this paper. See the file "READ_ME.txt" for instructions explaining how to run the code. [file 1471-2105-10-191-S5.zip › Output/Distributions - exon BioC/SCORE.sd.norm.int_Fig1.tif]

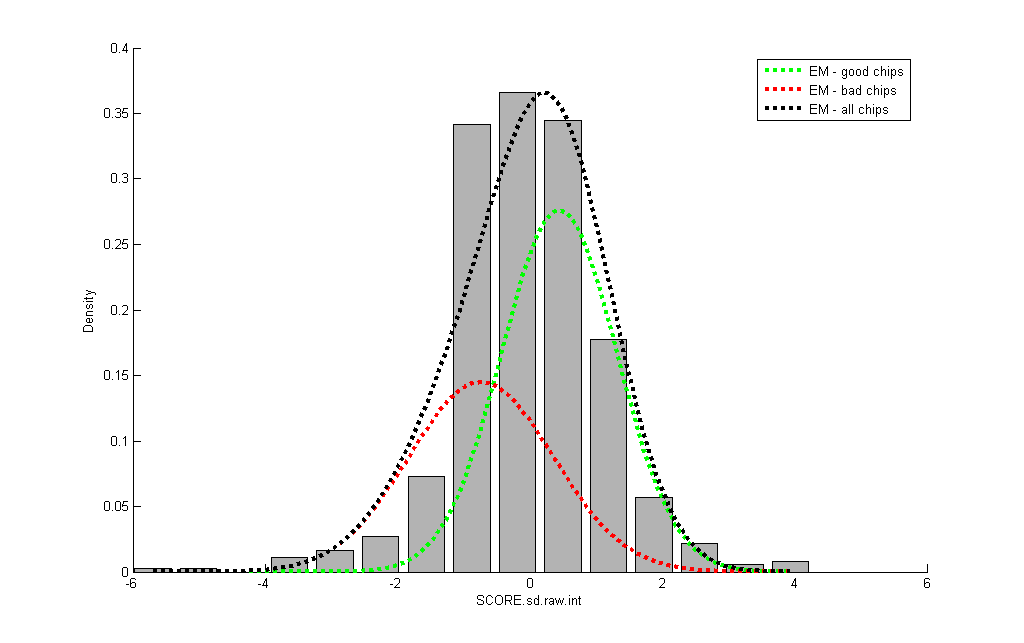

Supplement: Additional file 5 — – SourceCode. Zipped archive contains Matlab source code used for the analyses described in this paper. See the file "READ_ME.txt" for instructions explaining how to run the code. [file 1471-2105-10-191-S5.zip › Output/Distributions - exon BioC/SCORE.sd.raw.int_Fig1.tif]

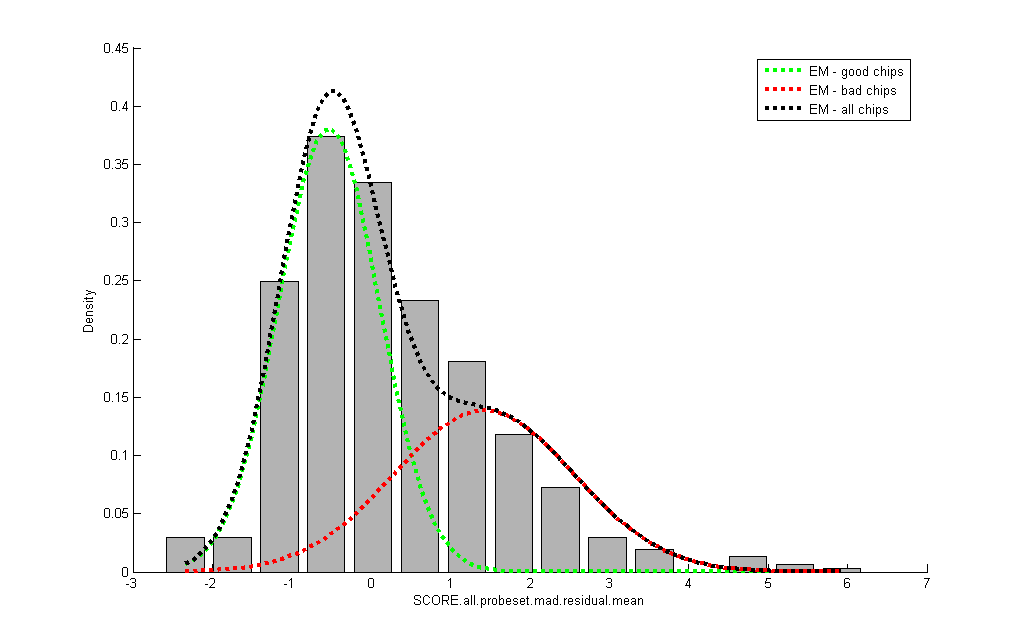

Supplement: Additional file 5 — – SourceCode. Zipped archive contains Matlab source code used for the analyses described in this paper. See the file "READ_ME.txt" for instructions explaining how to run the code. [file 1471-2105-10-191-S5.zip › Output/Distributions - exon EC/SCORE.all.probeset.mad.residual.mean_Fig1.tif]

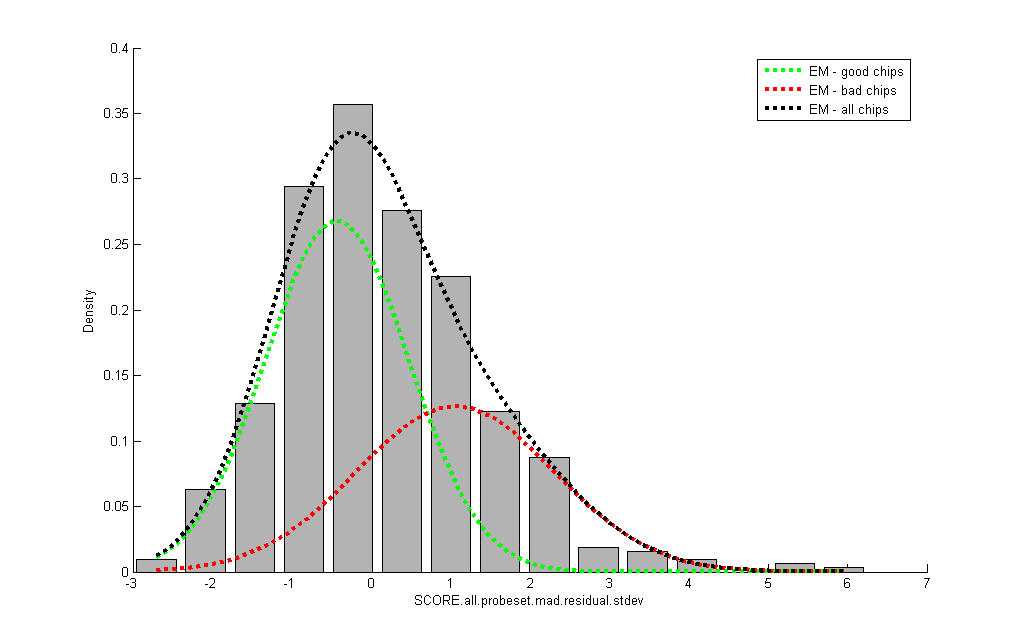

Supplement: Additional file 5 — – SourceCode. Zipped archive contains Matlab source code used for the analyses described in this paper. See the file "READ_ME.txt" for instructions explaining how to run the code. [file 1471-2105-10-191-S5.zip › Output/Distributions - exon EC/SCORE.all.probeset.mad.residual.stdev_Fig1.tif]

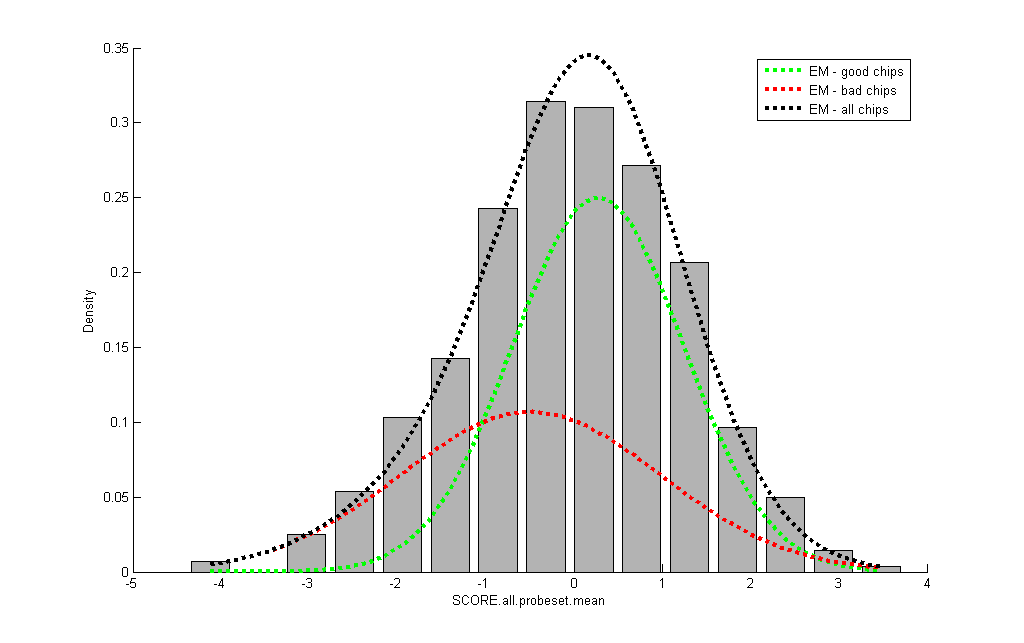

Supplement: Additional file 5 — – SourceCode. Zipped archive contains Matlab source code used for the analyses described in this paper. See the file "READ_ME.txt" for instructions explaining how to run the code. [file 1471-2105-10-191-S5.zip › Output/Distributions - exon EC/SCORE.all.probeset.mean_Fig1.tif]

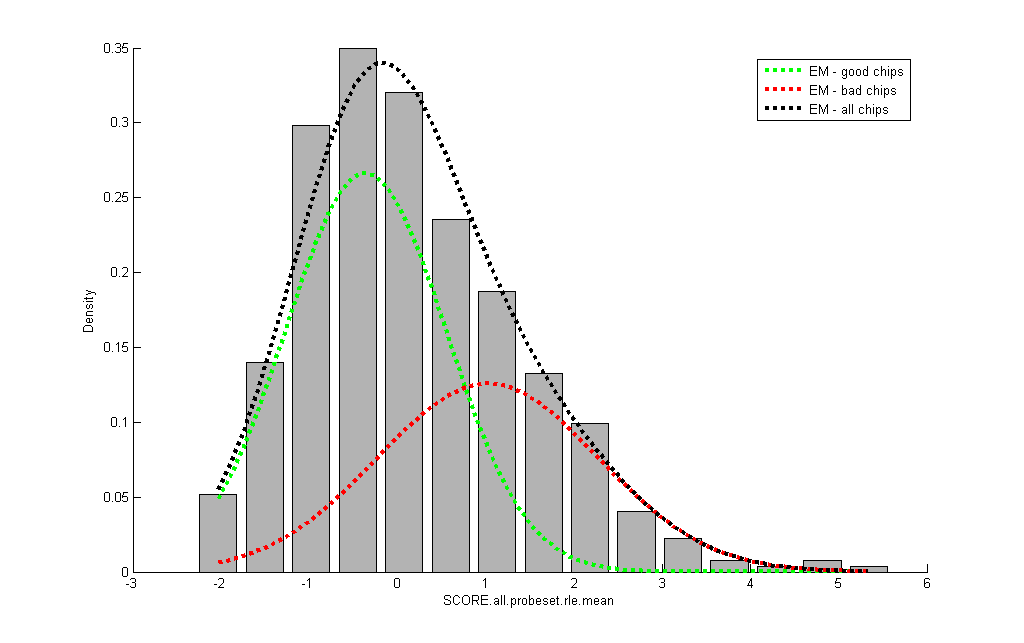

Supplement: Additional file 5 — – SourceCode. Zipped archive contains Matlab source code used for the analyses described in this paper. See the file "READ_ME.txt" for instructions explaining how to run the code. [file 1471-2105-10-191-S5.zip › Output/Distributions - exon EC/SCORE.all.probeset.rle.mean_Fig1.tif]

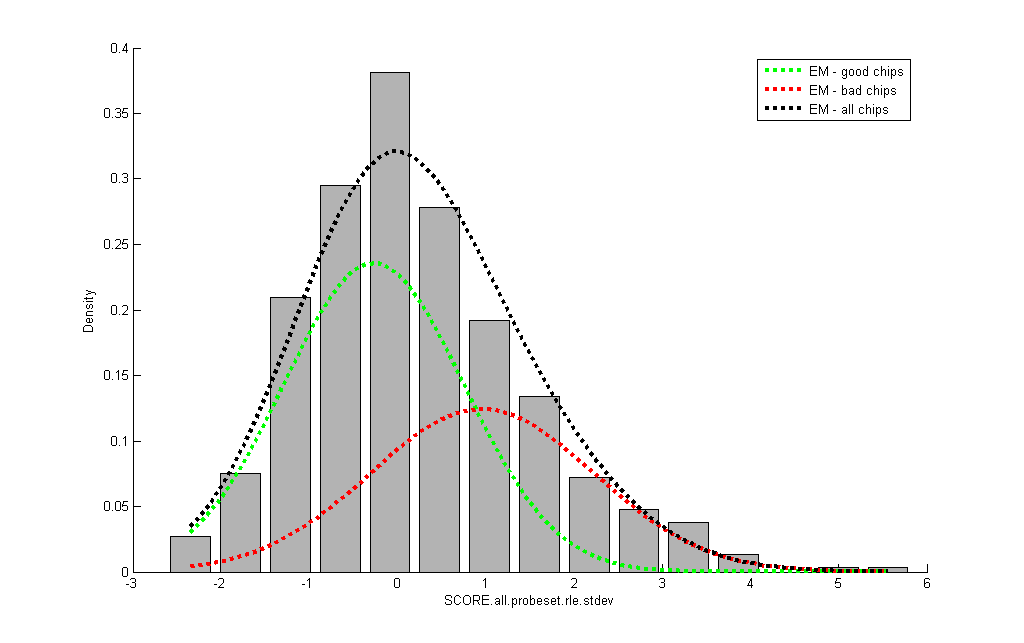

Supplement: Additional file 5 — – SourceCode. Zipped archive contains Matlab source code used for the analyses described in this paper. See the file "READ_ME.txt" for instructions explaining how to run the code. [file 1471-2105-10-191-S5.zip › Output/Distributions - exon EC/SCORE.all.probeset.rle.stdev_Fig1.tif]

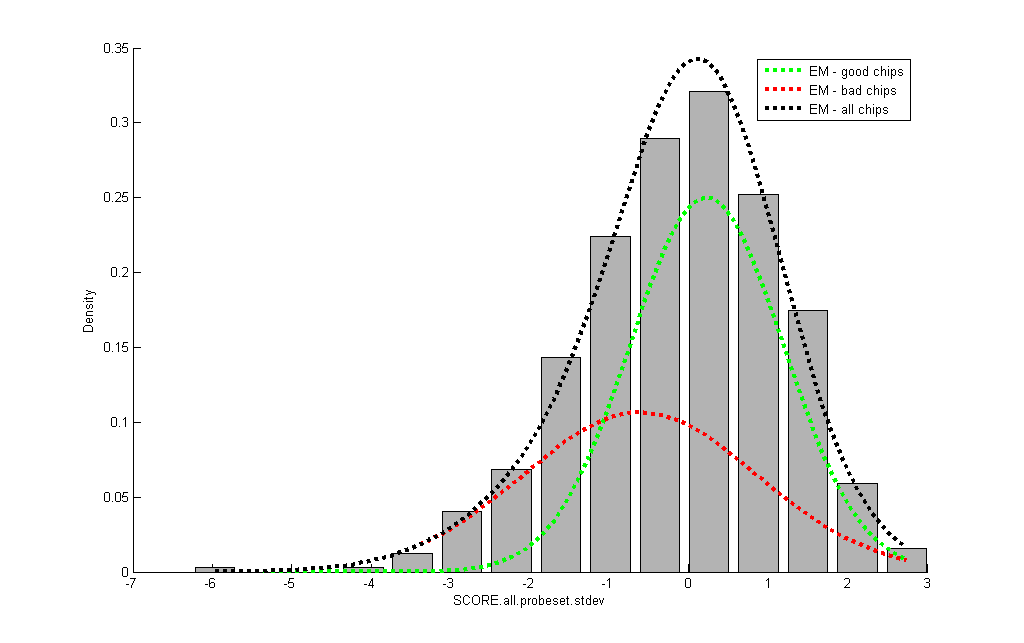

Supplement: Additional file 5 — – SourceCode. Zipped archive contains Matlab source code used for the analyses described in this paper. See the file "READ_ME.txt" for instructions explaining how to run the code. [file 1471-2105-10-191-S5.zip › Output/Distributions - exon EC/SCORE.all.probeset.stdev_Fig1.tif]

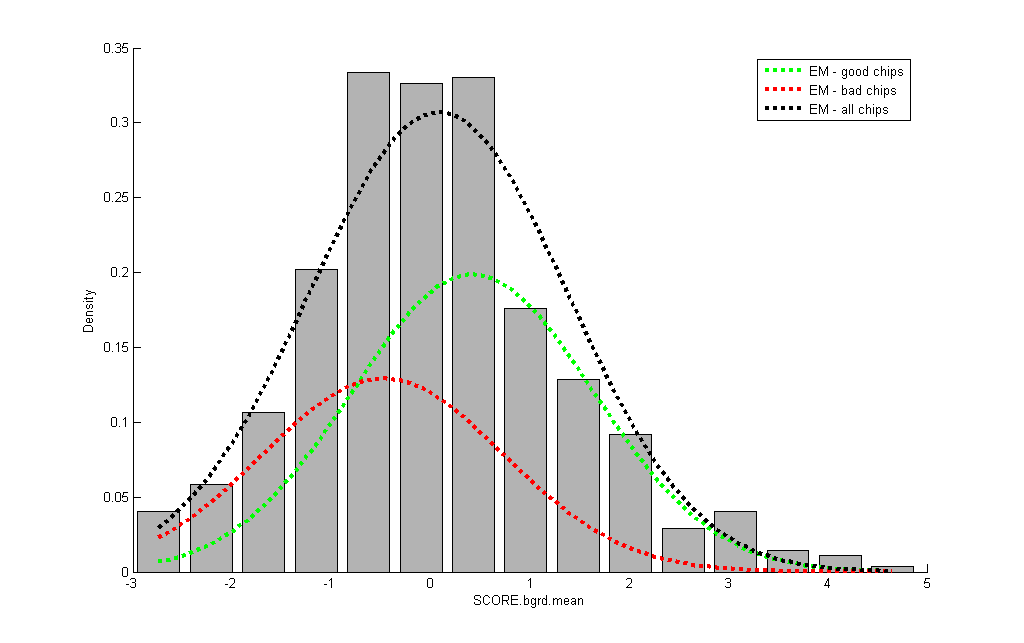

Supplement: Additional file 5 — – SourceCode. Zipped archive contains Matlab source code used for the analyses described in this paper. See the file "READ_ME.txt" for instructions explaining how to run the code. [file 1471-2105-10-191-S5.zip › Output/Distributions - exon EC/SCORE.bgrd.mean_Fig1.tif]

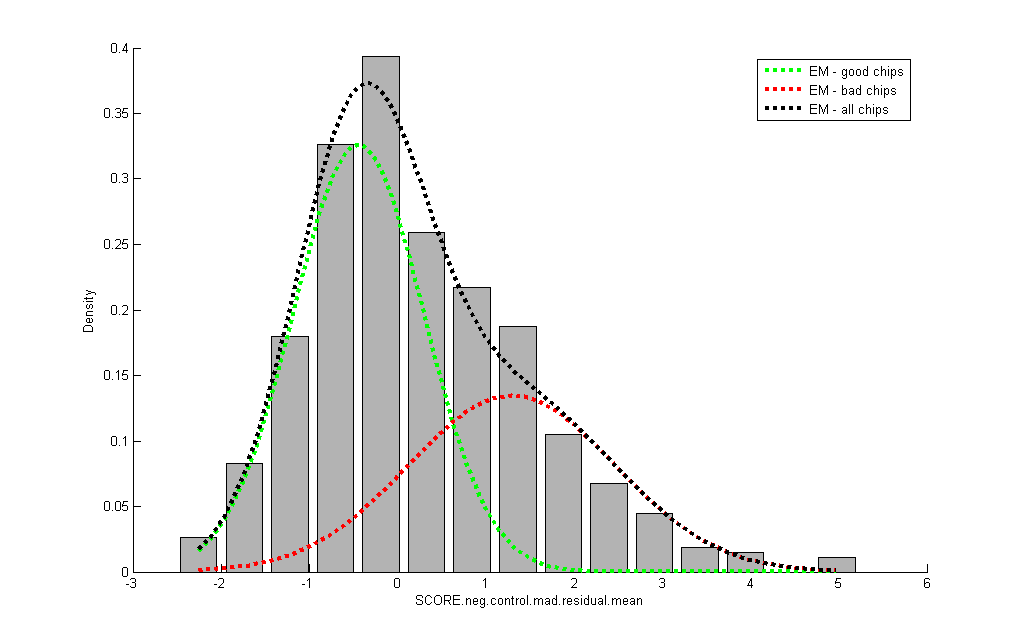

Supplement: Additional file 5 — – SourceCode. Zipped archive contains Matlab source code used for the analyses described in this paper. See the file "READ_ME.txt" for instructions explaining how to run the code. [file 1471-2105-10-191-S5.zip › Output/Distributions - exon EC/SCORE.neg.control.mad.residual.mean_Fig1.tif]

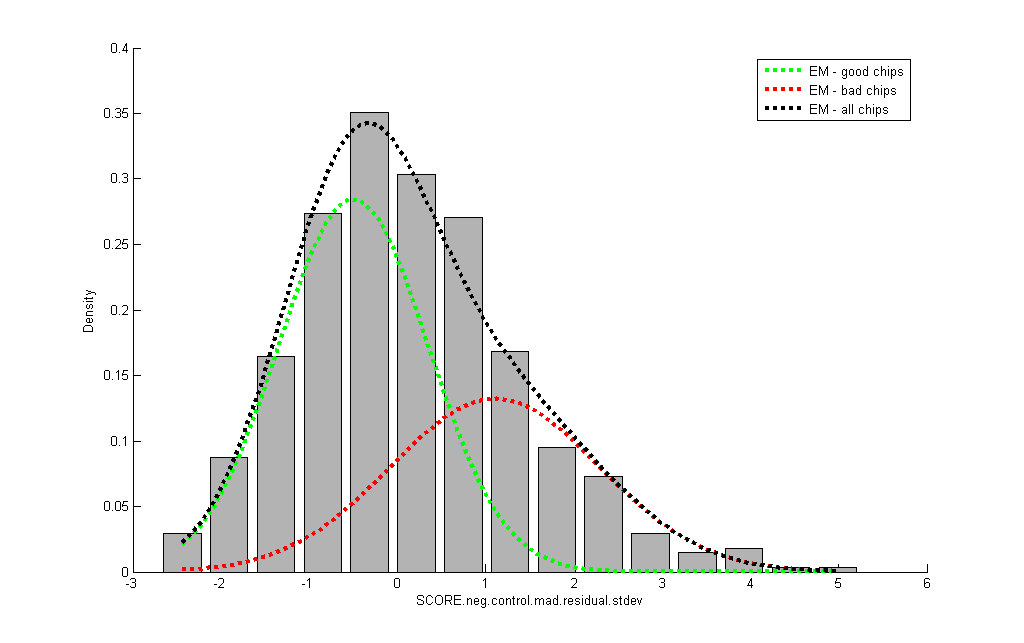

Supplement: Additional file 5 — – SourceCode. Zipped archive contains Matlab source code used for the analyses described in this paper. See the file "READ_ME.txt" for instructions explaining how to run the code. [file 1471-2105-10-191-S5.zip › Output/Distributions - exon EC/SCORE.neg.control.mad.residual.stdev_Fig1.tif]

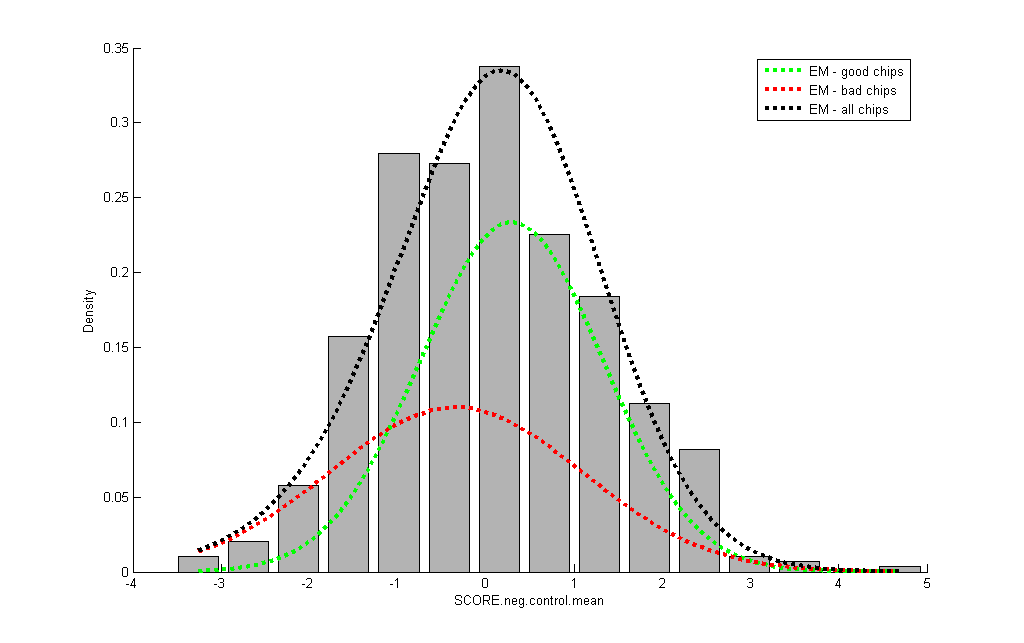

Supplement: Additional file 5 — – SourceCode. Zipped archive contains Matlab source code used for the analyses described in this paper. See the file "READ_ME.txt" for instructions explaining how to run the code. [file 1471-2105-10-191-S5.zip › Output/Distributions - exon EC/SCORE.neg.control.mean_Fig1.tif]

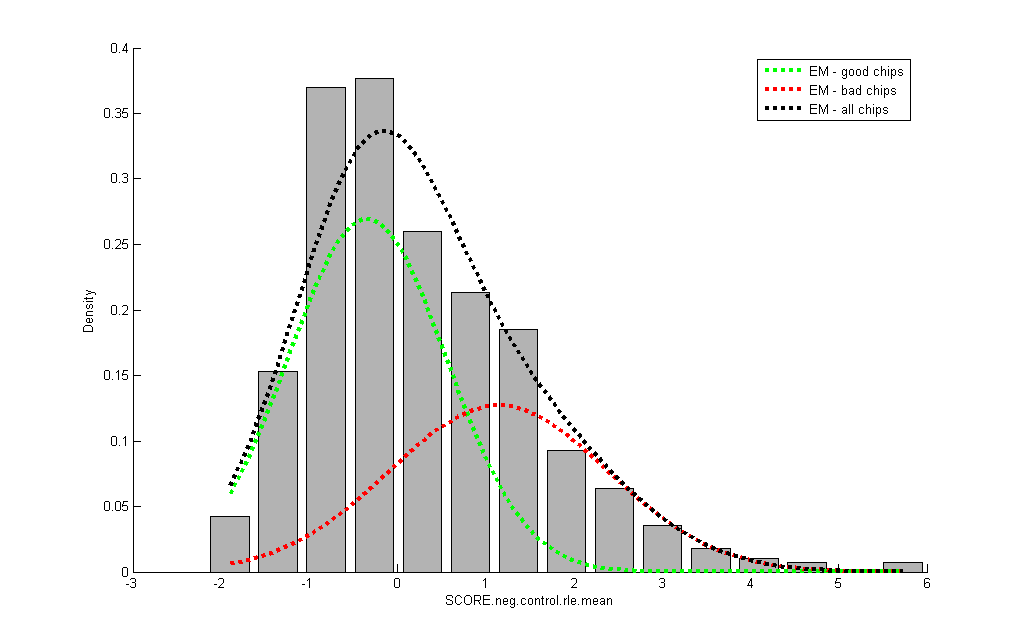

Supplement: Additional file 5 — – SourceCode. Zipped archive contains Matlab source code used for the analyses described in this paper. See the file "READ_ME.txt" for instructions explaining how to run the code. [file 1471-2105-10-191-S5.zip › Output/Distributions - exon EC/SCORE.neg.control.rle.mean_Fig1.tif]

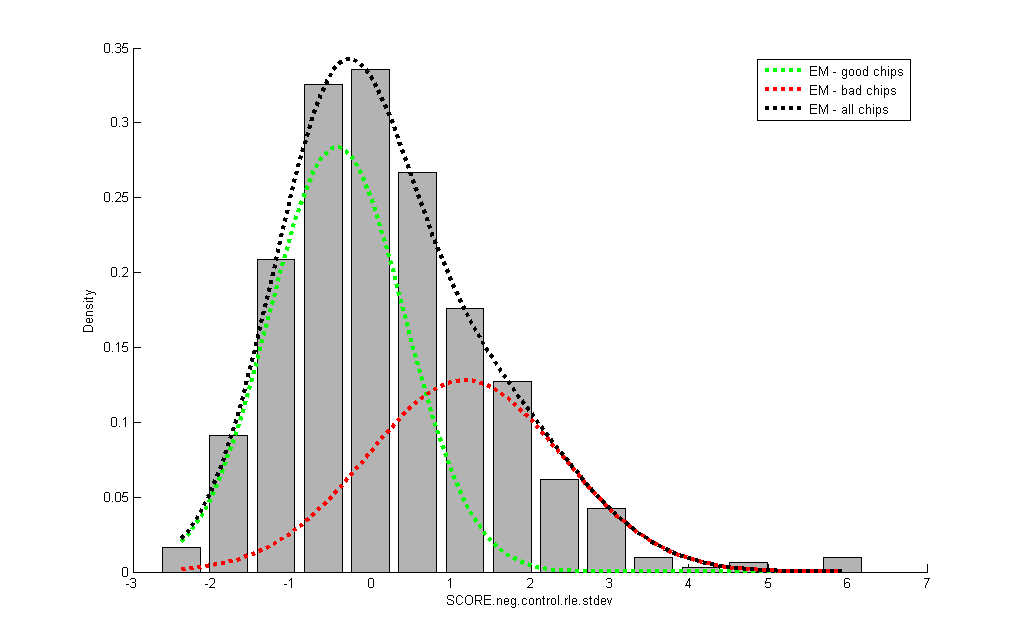

Supplement: Additional file 5 — – SourceCode. Zipped archive contains Matlab source code used for the analyses described in this paper. See the file "READ_ME.txt" for instructions explaining how to run the code. [file 1471-2105-10-191-S5.zip › Output/Distributions - exon EC/SCORE.neg.control.rle.stdev_Fig1.tif]

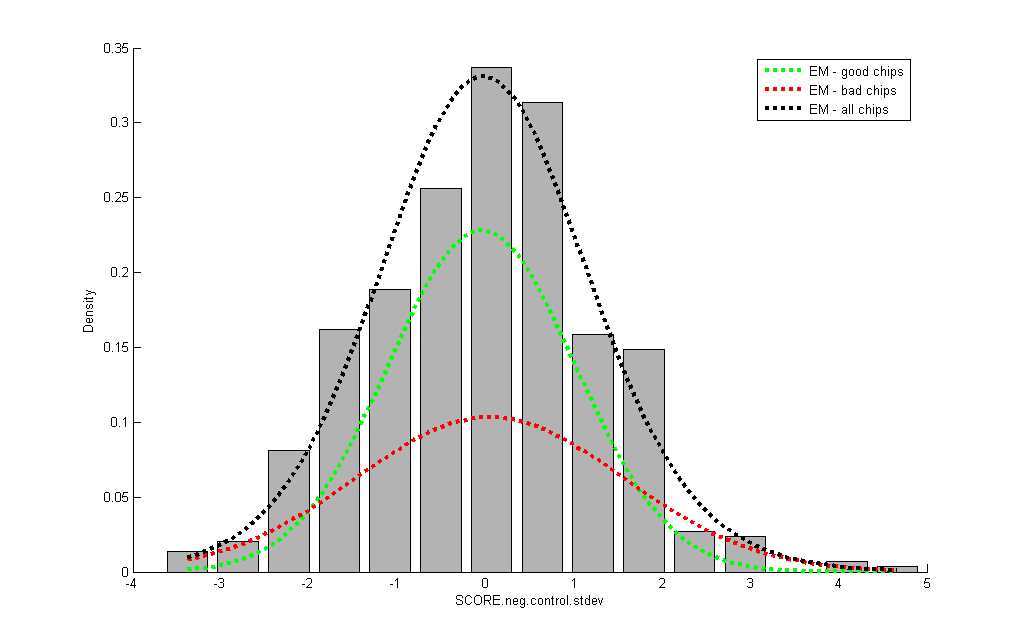

Supplement: Additional file 5 — – SourceCode. Zipped archive contains Matlab source code used for the analyses described in this paper. See the file "READ_ME.txt" for instructions explaining how to run the code. [file 1471-2105-10-191-S5.zip › Output/Distributions - exon EC/SCORE.neg.control.stdev_Fig1.tif]

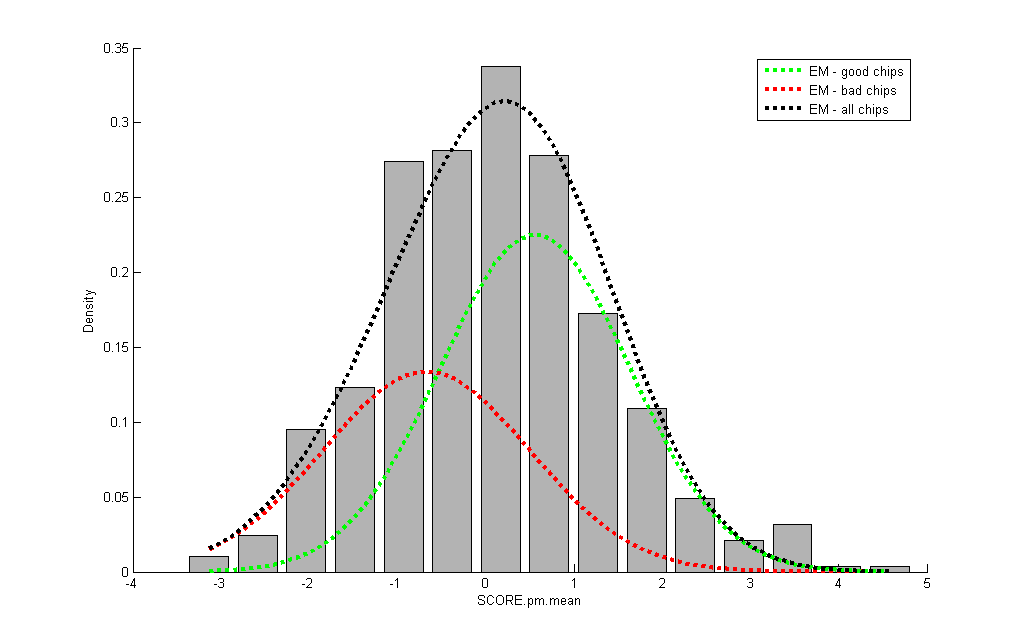

Supplement: Additional file 5 — – SourceCode. Zipped archive contains Matlab source code used for the analyses described in this paper. See the file "READ_ME.txt" for instructions explaining how to run the code. [file 1471-2105-10-191-S5.zip › Output/Distributions - exon EC/SCORE.pm.mean_Fig1.tif]

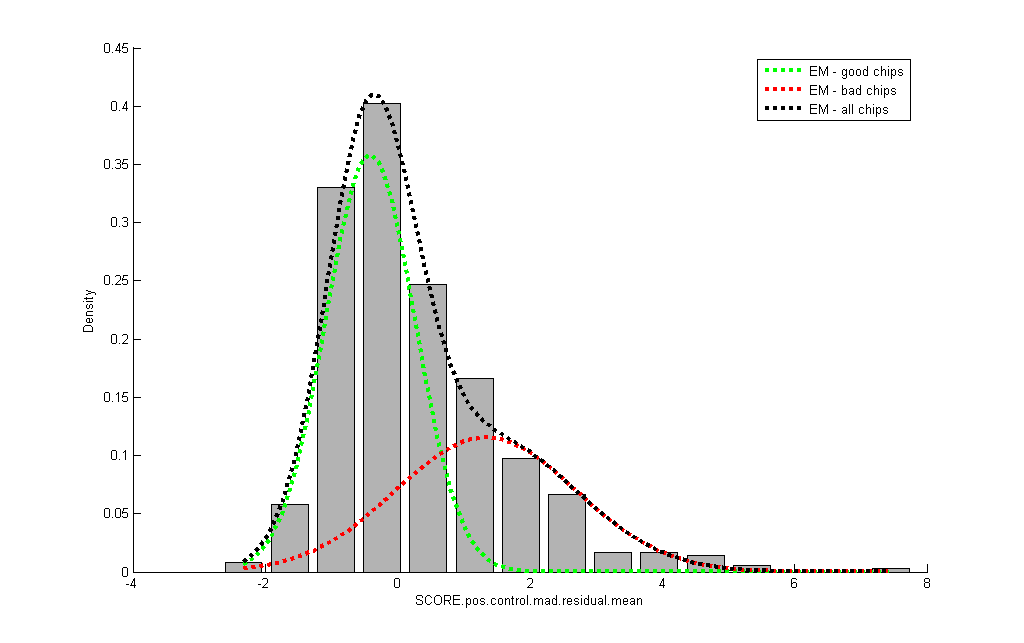

Supplement: Additional file 5 — – SourceCode. Zipped archive contains Matlab source code used for the analyses described in this paper. See the file "READ_ME.txt" for instructions explaining how to run the code. [file 1471-2105-10-191-S5.zip › Output/Distributions - exon EC/SCORE.pos.control.mad.residual.mean_Fig1.tif]

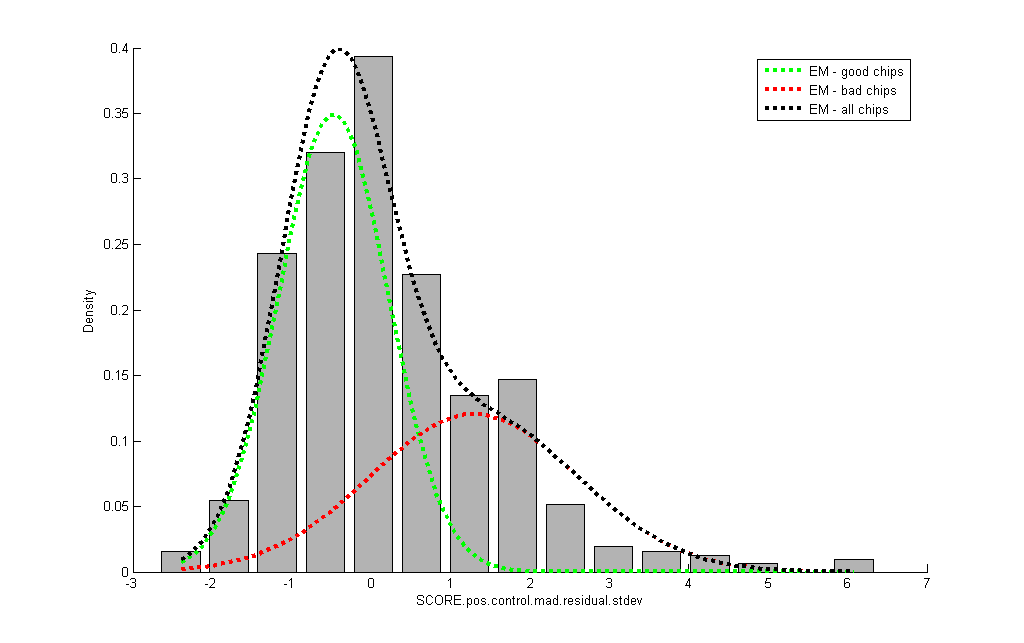

Supplement: Additional file 5 — – SourceCode. Zipped archive contains Matlab source code used for the analyses described in this paper. See the file "READ_ME.txt" for instructions explaining how to run the code. [file 1471-2105-10-191-S5.zip › Output/Distributions - exon EC/SCORE.pos.control.mad.residual.stdev_Fig1.tif]

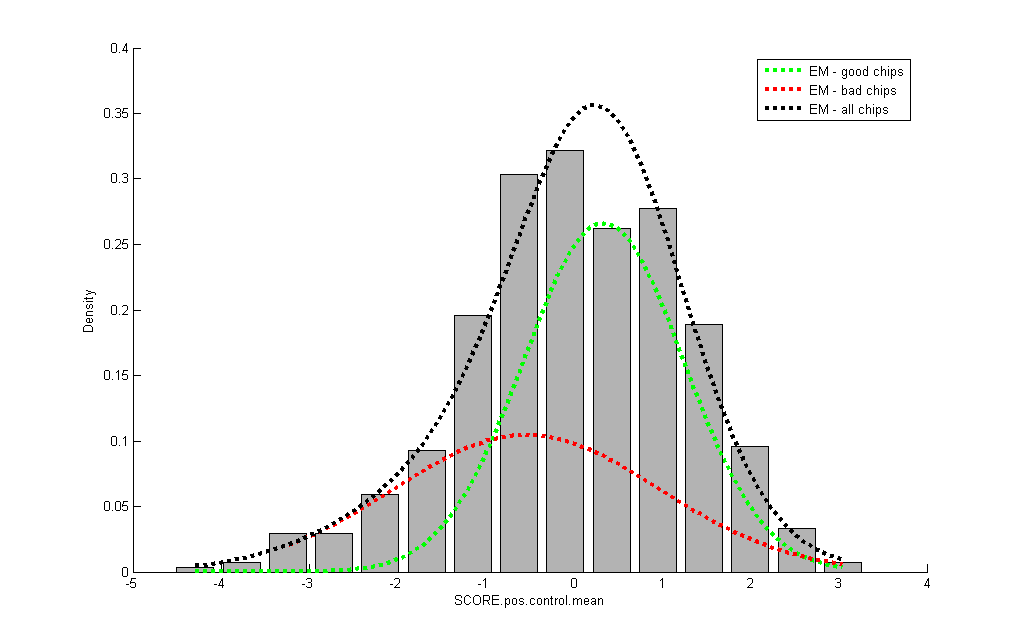

Supplement: Additional file 5 — – SourceCode. Zipped archive contains Matlab source code used for the analyses described in this paper. See the file "READ_ME.txt" for instructions explaining how to run the code. [file 1471-2105-10-191-S5.zip › Output/Distributions - exon EC/SCORE.pos.control.mean_Fig1.tif]

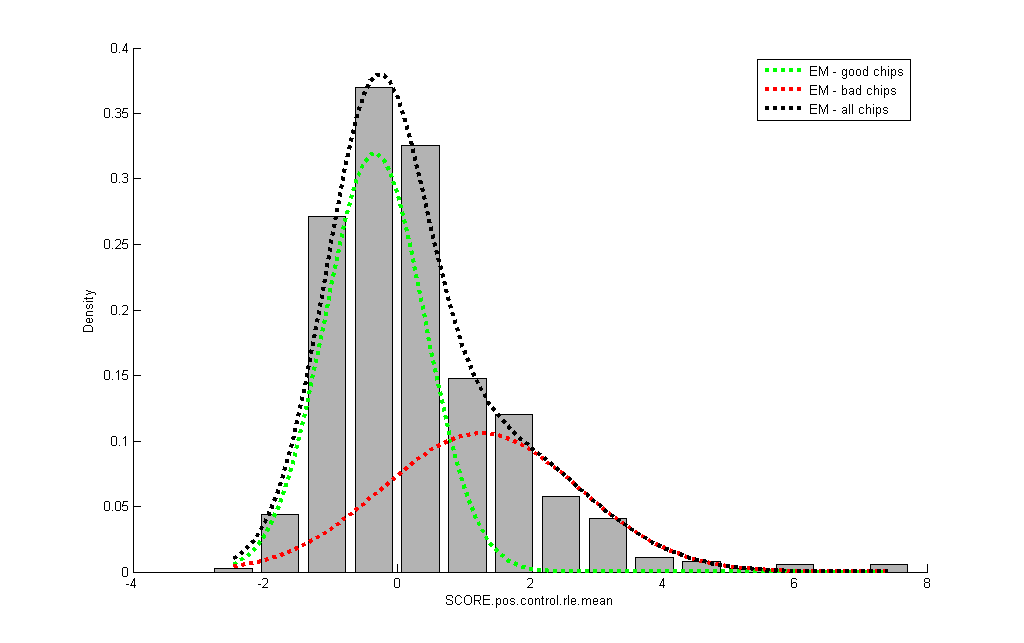

Supplement: Additional file 5 — – SourceCode. Zipped archive contains Matlab source code used for the analyses described in this paper. See the file "READ_ME.txt" for instructions explaining how to run the code. [file 1471-2105-10-191-S5.zip › Output/Distributions - exon EC/SCORE.pos.control.rle.mean_Fig1.tif]

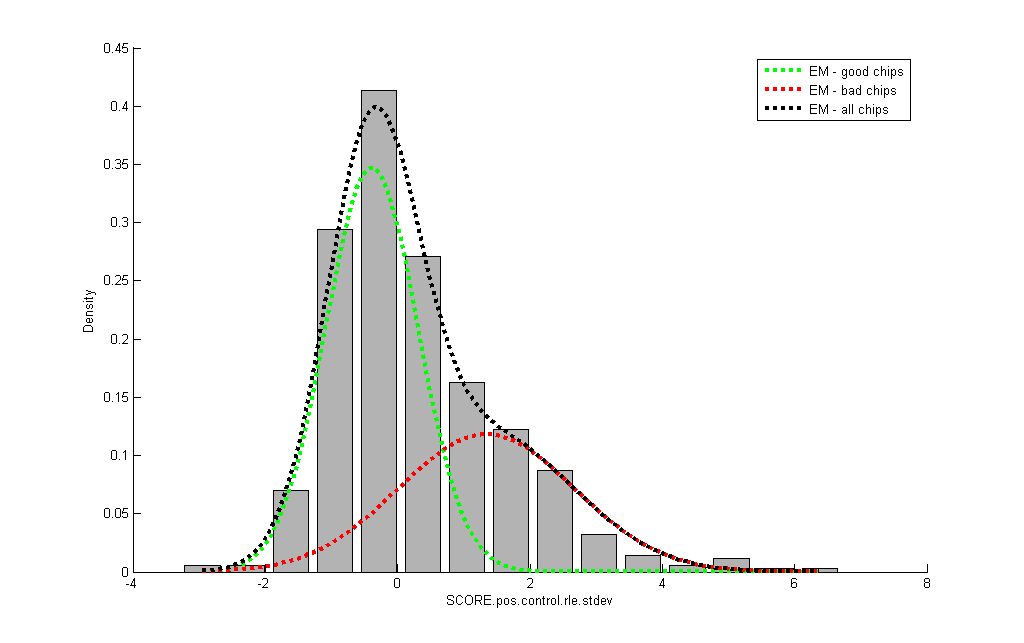

Supplement: Additional file 5 — – SourceCode. Zipped archive contains Matlab source code used for the analyses described in this paper. See the file "READ_ME.txt" for instructions explaining how to run the code. [file 1471-2105-10-191-S5.zip › Output/Distributions - exon EC/SCORE.pos.control.rle.stdev_Fig1.tif]

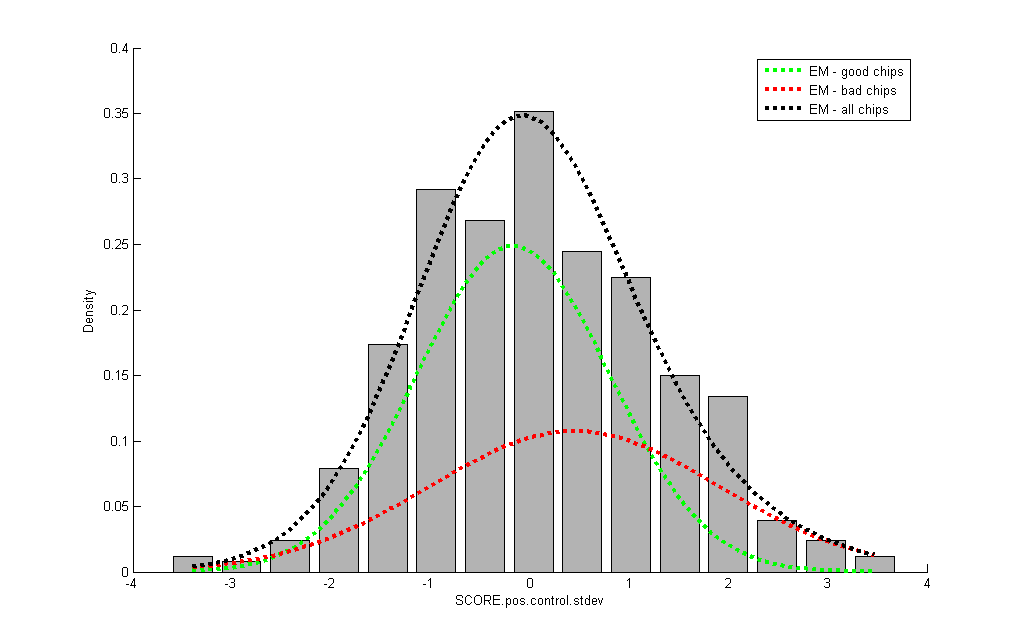

Supplement: Additional file 5 — – SourceCode. Zipped archive contains Matlab source code used for the analyses described in this paper. See the file "READ_ME.txt" for instructions explaining how to run the code. [file 1471-2105-10-191-S5.zip › Output/Distributions - exon EC/SCORE.pos.control.stdev_Fig1.tif]

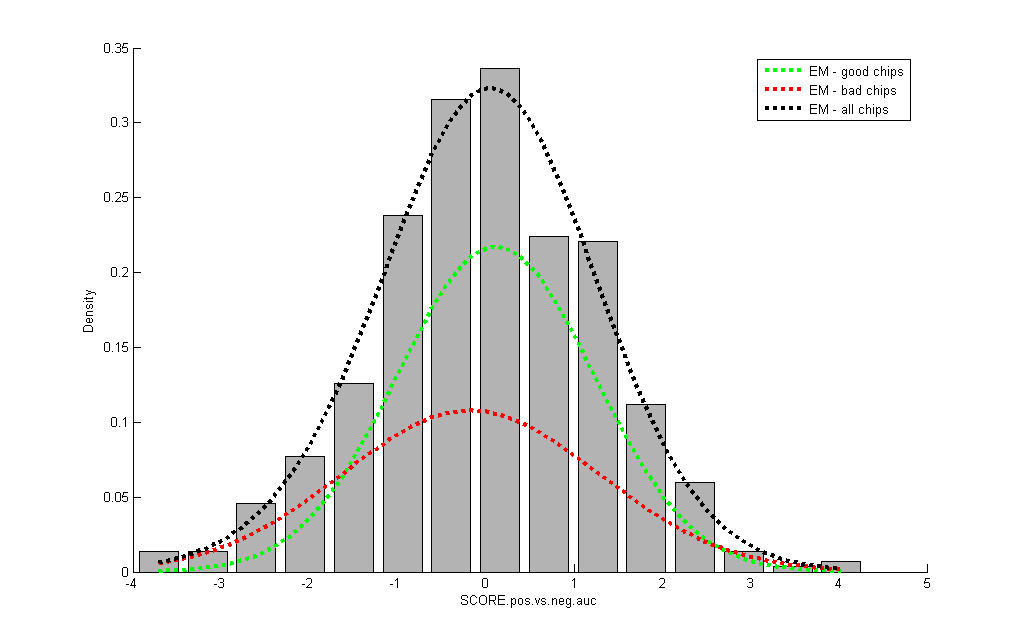

Supplement: Additional file 5 — – SourceCode. Zipped archive contains Matlab source code used for the analyses described in this paper. See the file "READ_ME.txt" for instructions explaining how to run the code. [file 1471-2105-10-191-S5.zip › Output/Distributions - exon EC/SCORE.pos.vs.neg.auc_Fig1.tif]

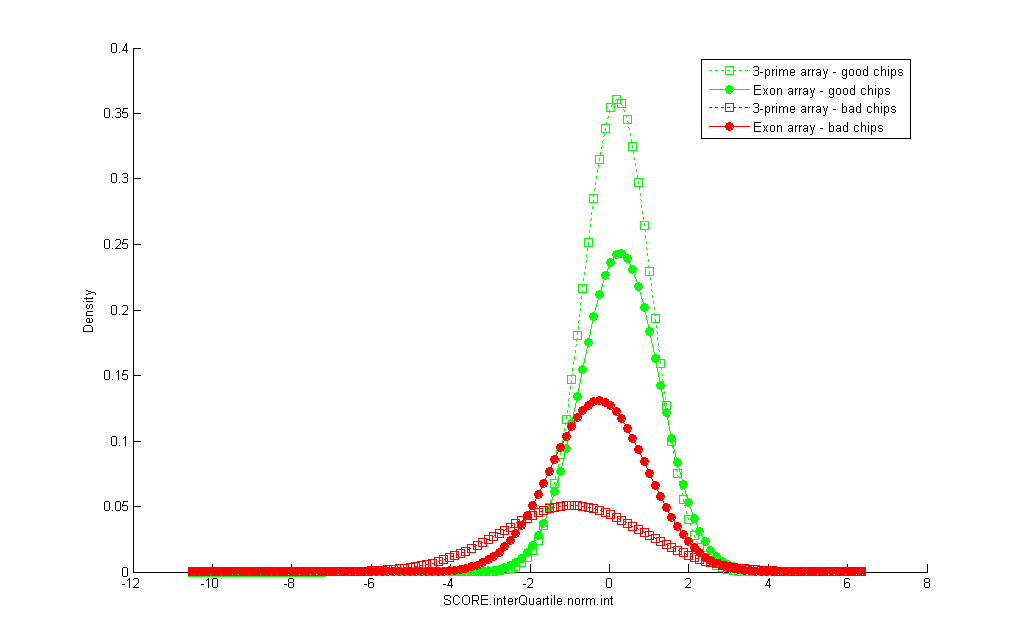

Supplement: Additional file 5 — – SourceCode. Zipped archive contains Matlab source code used for the analyses described in this paper. See the file "READ_ME.txt" for instructions explaining how to run the code. [file 1471-2105-10-191-S5.zip › Output/Distributions - exon versus 3-prime/SCORE.interQuartile.norm.int_Fig1.tif]

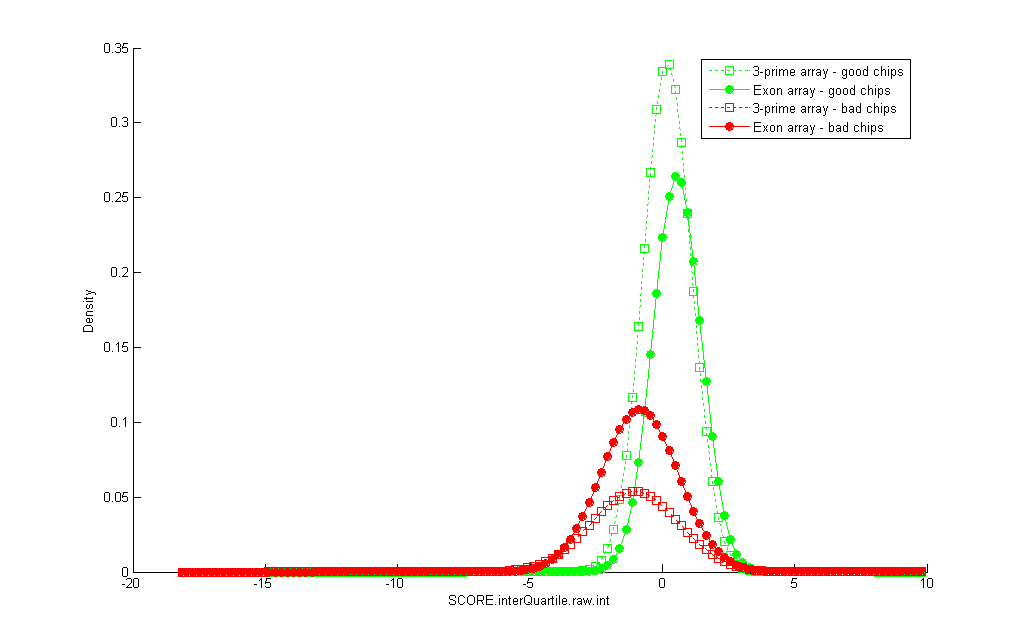

Supplement: Additional file 5 — – SourceCode. Zipped archive contains Matlab source code used for the analyses described in this paper. See the file "READ_ME.txt" for instructions explaining how to run the code. [file 1471-2105-10-191-S5.zip › Output/Distributions - exon versus 3-prime/SCORE.interQuartile.raw.int_Fig1.tif]

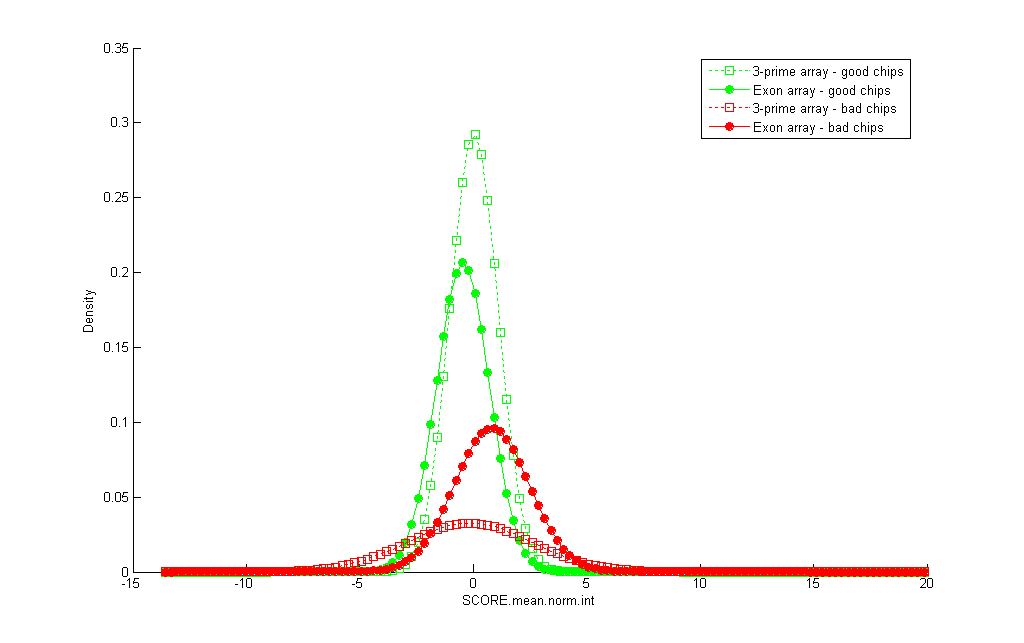

Supplement: Additional file 5 — – SourceCode. Zipped archive contains Matlab source code used for the analyses described in this paper. See the file "READ_ME.txt" for instructions explaining how to run the code. [file 1471-2105-10-191-S5.zip › Output/Distributions - exon versus 3-prime/SCORE.mean.norm.int_Fig1.tif]

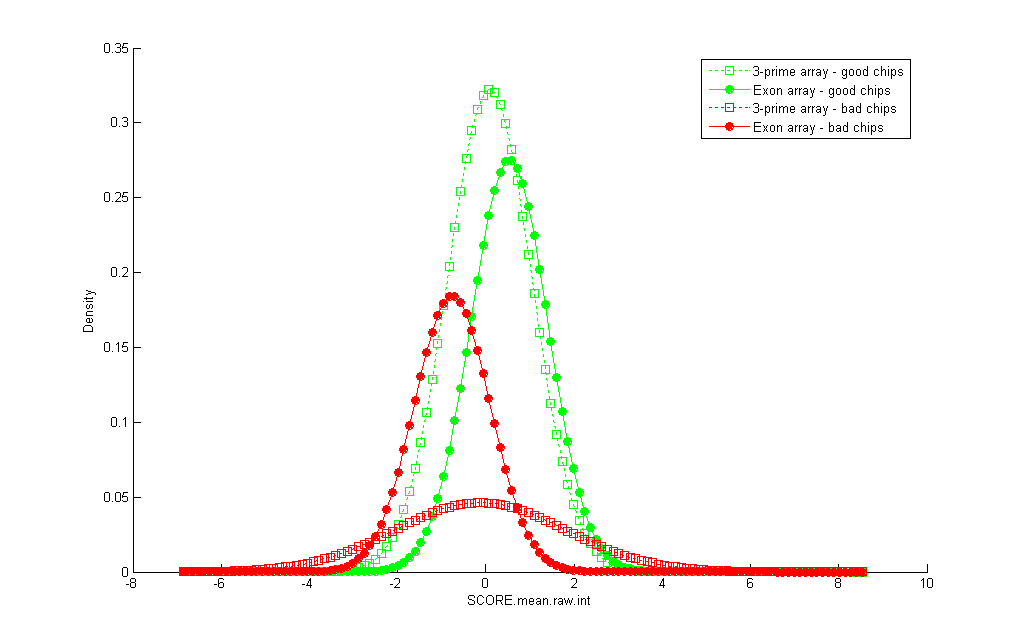

Supplement: Additional file 5 — – SourceCode. Zipped archive contains Matlab source code used for the analyses described in this paper. See the file "READ_ME.txt" for instructions explaining how to run the code. [file 1471-2105-10-191-S5.zip › Output/Distributions - exon versus 3-prime/SCORE.mean.raw.int_Fig1.tif]

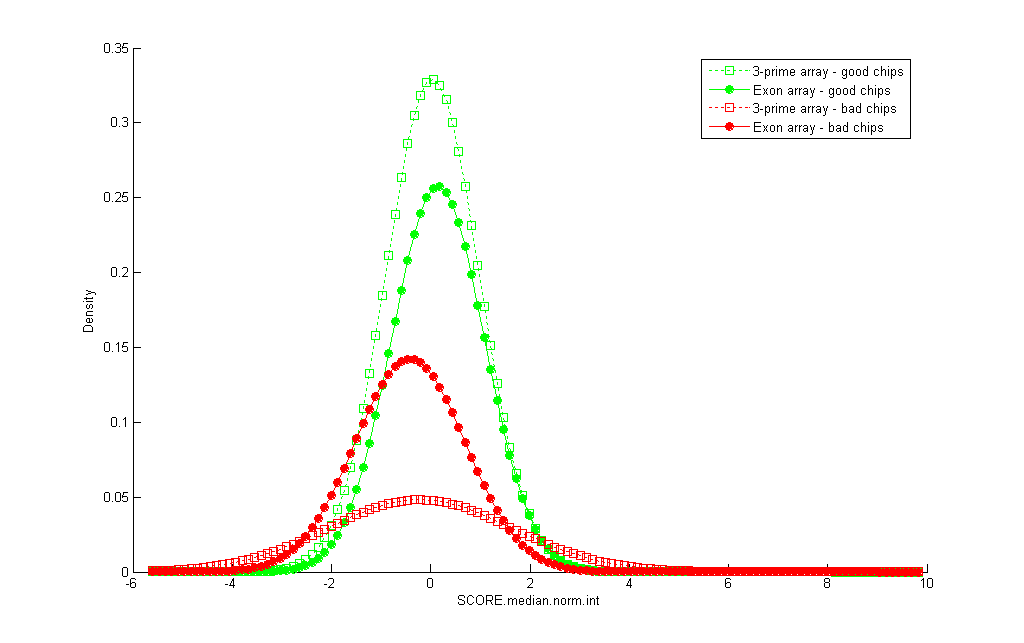

Supplement: Additional file 5 — – SourceCode. Zipped archive contains Matlab source code used for the analyses described in this paper. See the file "READ_ME.txt" for instructions explaining how to run the code. [file 1471-2105-10-191-S5.zip › Output/Distributions - exon versus 3-prime/SCORE.median.norm.int_Fig1.tif]

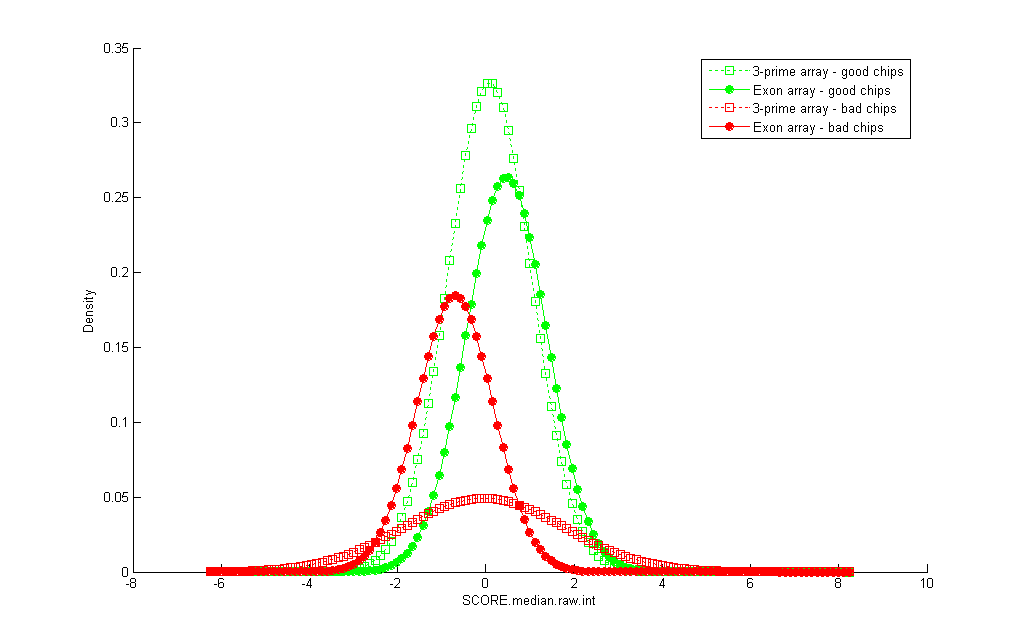

Supplement: Additional file 5 — – SourceCode. Zipped archive contains Matlab source code used for the analyses described in this paper. See the file "READ_ME.txt" for instructions explaining how to run the code. [file 1471-2105-10-191-S5.zip › Output/Distributions - exon versus 3-prime/SCORE.median.raw.int_Fig1.tif]

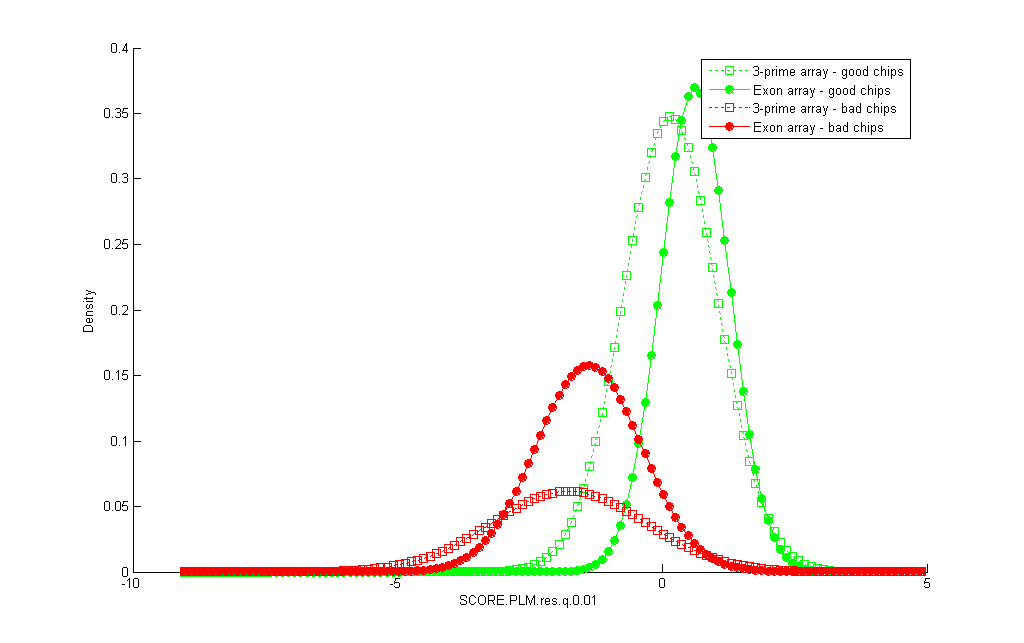

Supplement: Additional file 5 — – SourceCode. Zipped archive contains Matlab source code used for the analyses described in this paper. See the file "READ_ME.txt" for instructions explaining how to run the code. [file 1471-2105-10-191-S5.zip › Output/Distributions - exon versus 3-prime/SCORE.PLM.res.q.0.01_Fig1.tif]

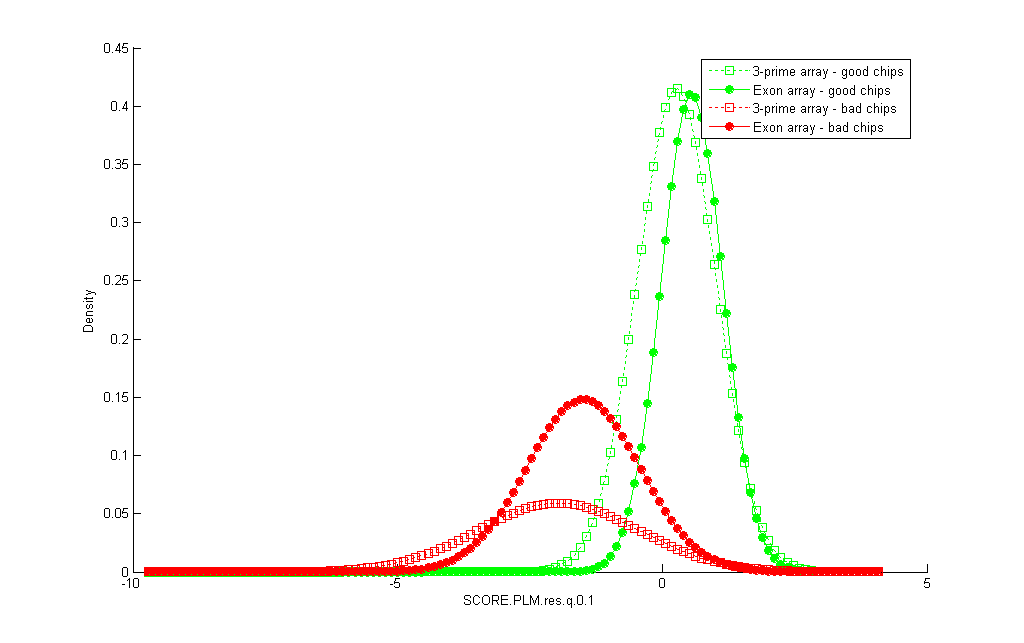

Supplement: Additional file 5 — – SourceCode. Zipped archive contains Matlab source code used for the analyses described in this paper. See the file "READ_ME.txt" for instructions explaining how to run the code. [file 1471-2105-10-191-S5.zip › Output/Distributions - exon versus 3-prime/SCORE.PLM.res.q.0.1_Fig1.tif]

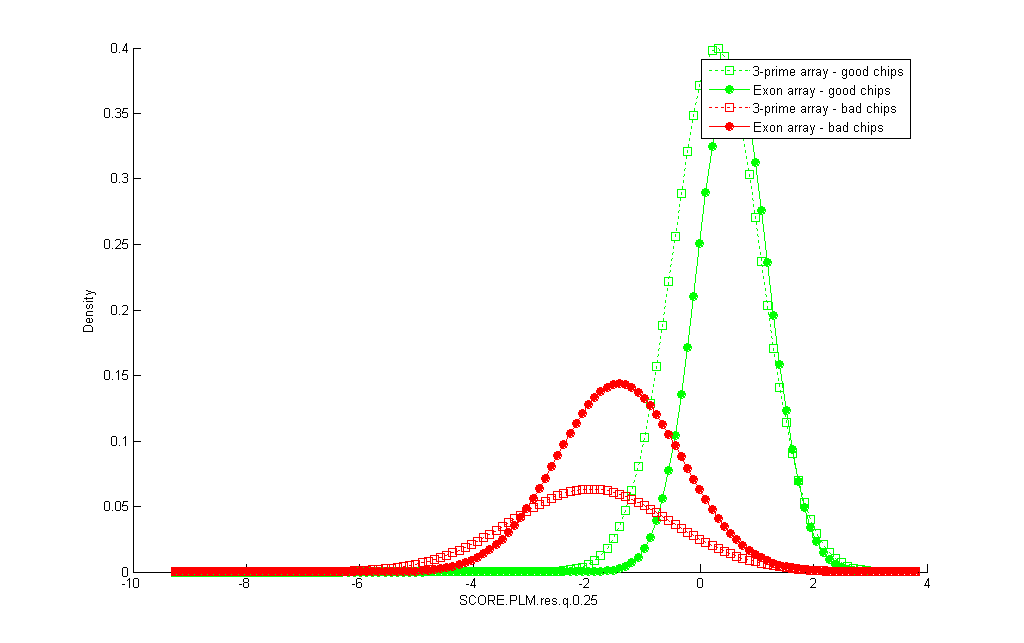

Supplement: Additional file 5 — – SourceCode. Zipped archive contains Matlab source code used for the analyses described in this paper. See the file "READ_ME.txt" for instructions explaining how to run the code. [file 1471-2105-10-191-S5.zip › Output/Distributions - exon versus 3-prime/SCORE.PLM.res.q.0.25_Fig1.tif]

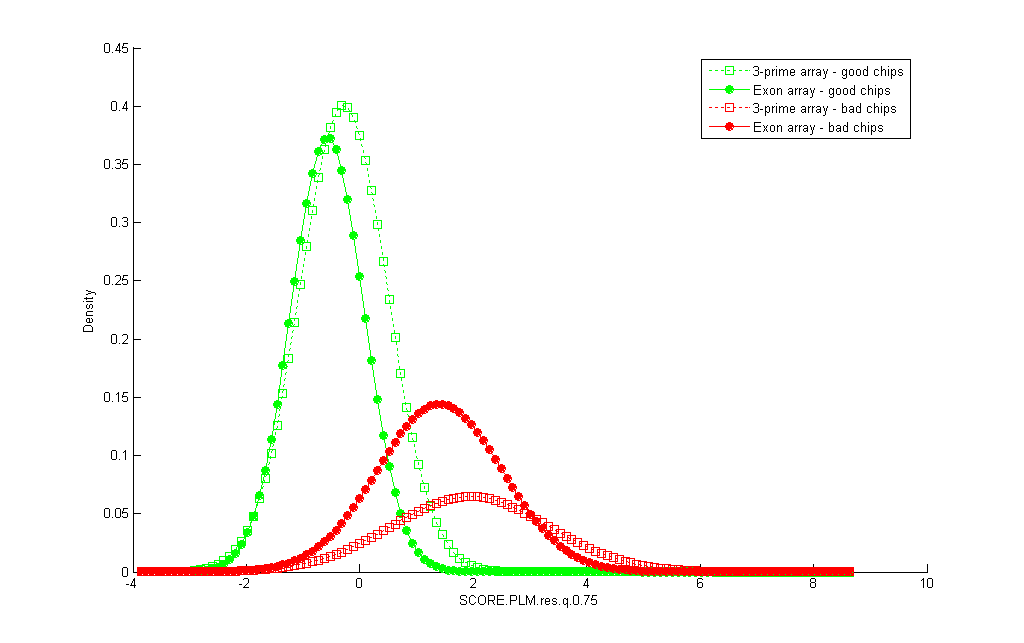

Supplement: Additional file 5 — – SourceCode. Zipped archive contains Matlab source code used for the analyses described in this paper. See the file "READ_ME.txt" for instructions explaining how to run the code. [file 1471-2105-10-191-S5.zip › Output/Distributions - exon versus 3-prime/SCORE.PLM.res.q.0.75_Fig1.tif]

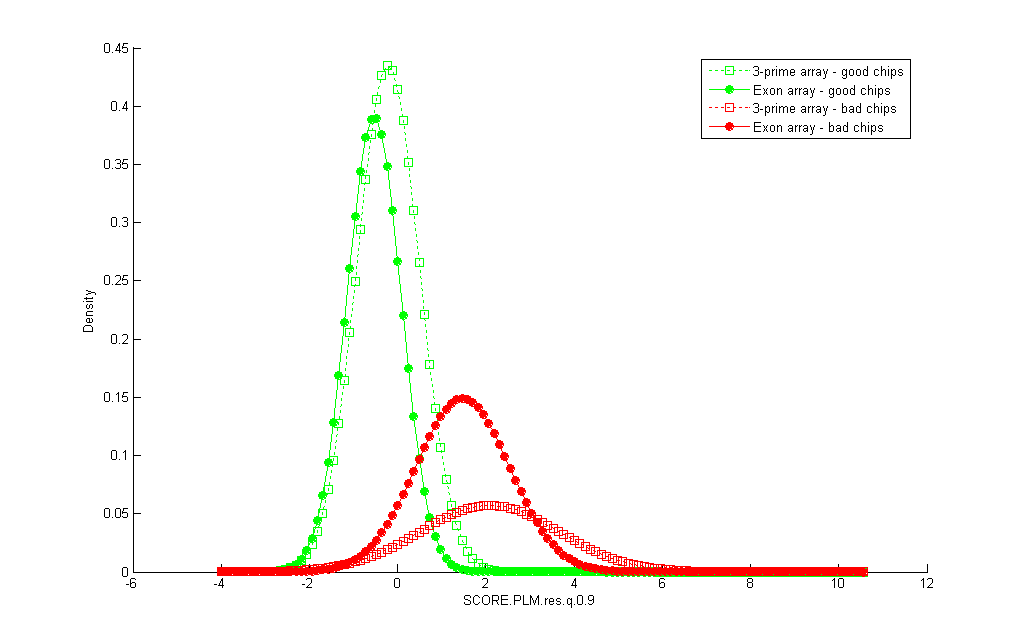

Supplement: Additional file 5 — – SourceCode. Zipped archive contains Matlab source code used for the analyses described in this paper. See the file "READ_ME.txt" for instructions explaining how to run the code. [file 1471-2105-10-191-S5.zip › Output/Distributions - exon versus 3-prime/SCORE.PLM.res.q.0.9_Fig1.tif]

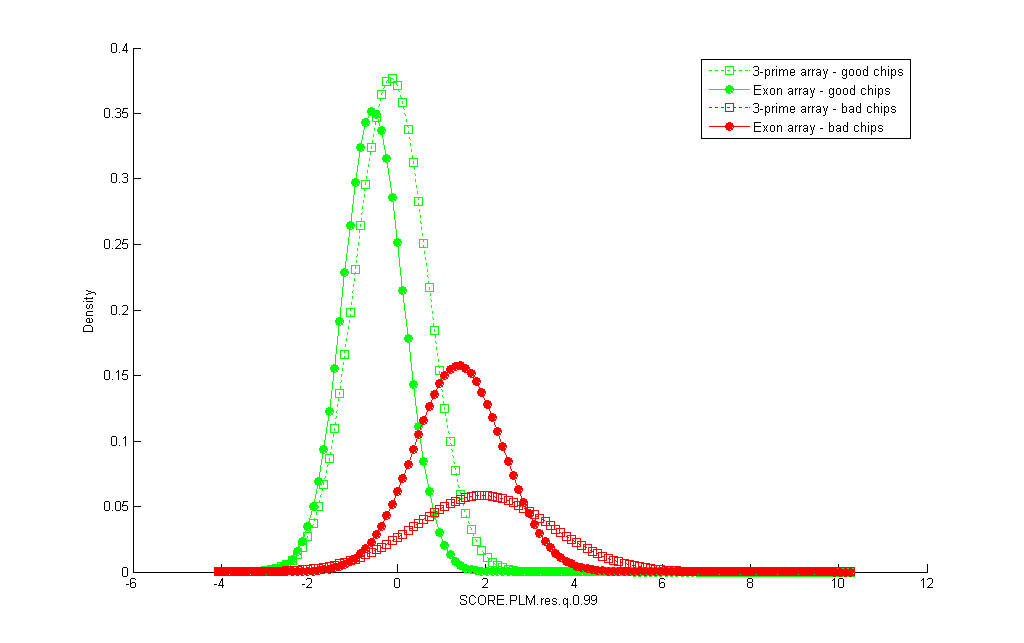

Supplement: Additional file 5 — – SourceCode. Zipped archive contains Matlab source code used for the analyses described in this paper. See the file "READ_ME.txt" for instructions explaining how to run the code. [file 1471-2105-10-191-S5.zip › Output/Distributions - exon versus 3-prime/SCORE.PLM.res.q.0.99_Fig1.tif]

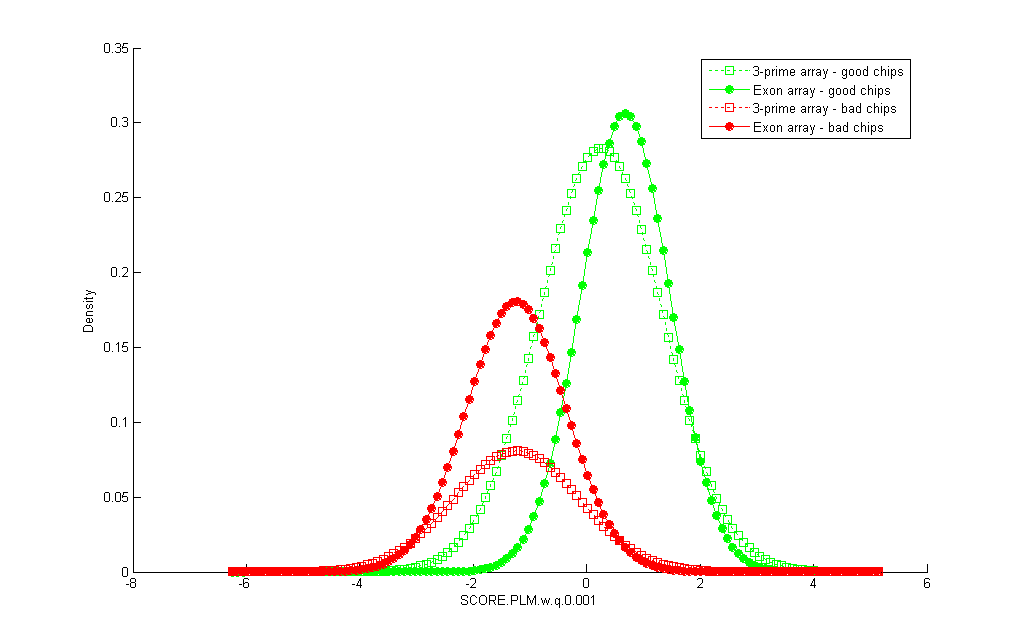

Supplement: Additional file 5 — – SourceCode. Zipped archive contains Matlab source code used for the analyses described in this paper. See the file "READ_ME.txt" for instructions explaining how to run the code. [file 1471-2105-10-191-S5.zip › Output/Distributions - exon versus 3-prime/SCORE.PLM.w.q.0.001_Fig1.tif]

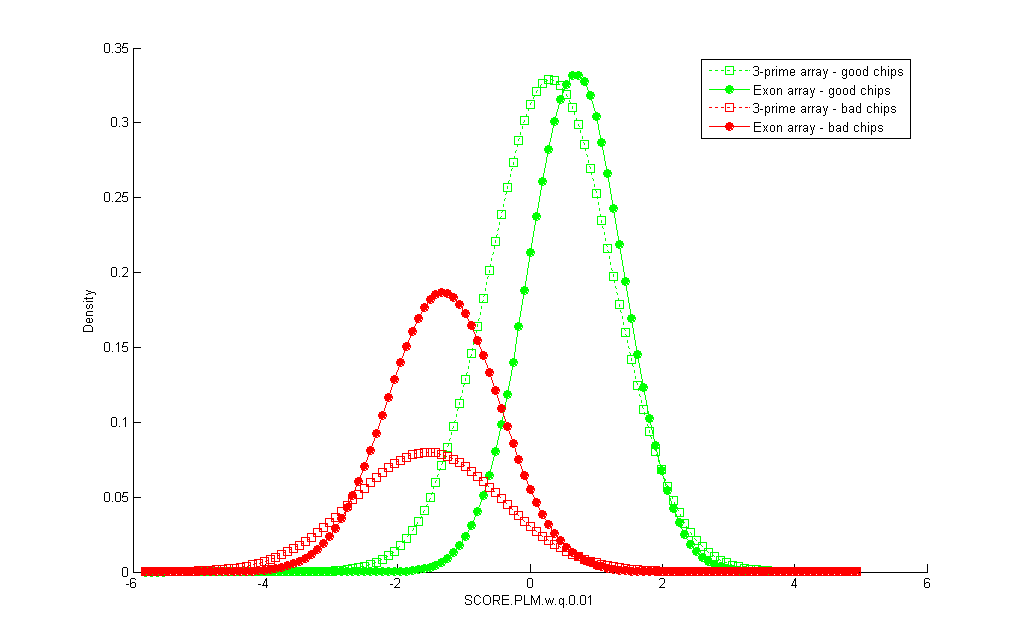

Supplement: Additional file 5 — – SourceCode. Zipped archive contains Matlab source code used for the analyses described in this paper. See the file "READ_ME.txt" for instructions explaining how to run the code. [file 1471-2105-10-191-S5.zip › Output/Distributions - exon versus 3-prime/SCORE.PLM.w.q.0.01_Fig1.tif]

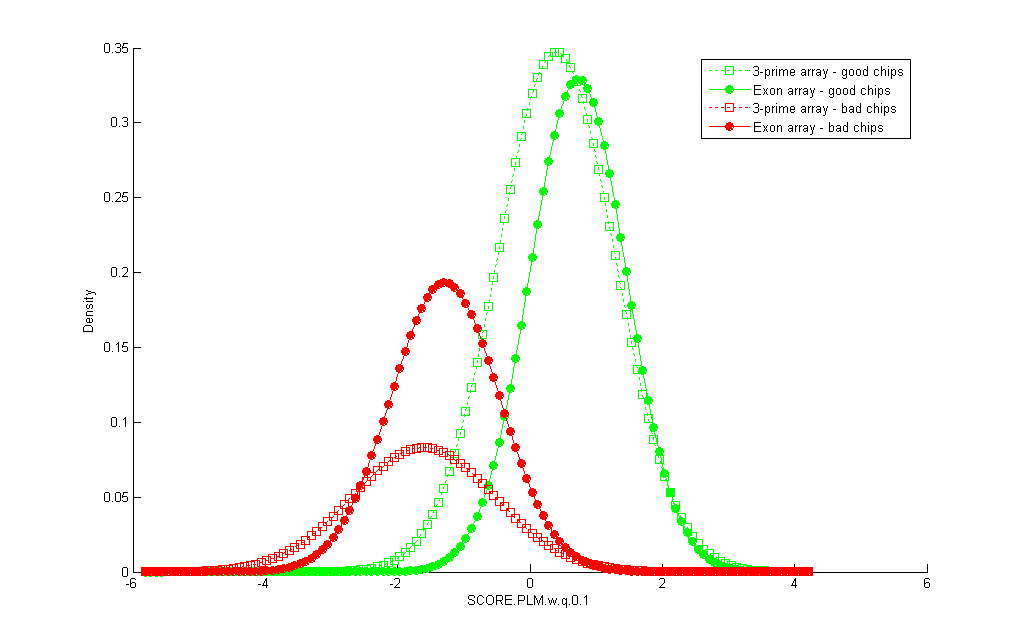

Supplement: Additional file 5 — – SourceCode. Zipped archive contains Matlab source code used for the analyses described in this paper. See the file "READ_ME.txt" for instructions explaining how to run the code. [file 1471-2105-10-191-S5.zip › Output/Distributions - exon versus 3-prime/SCORE.PLM.w.q.0.1_Fig1.tif]

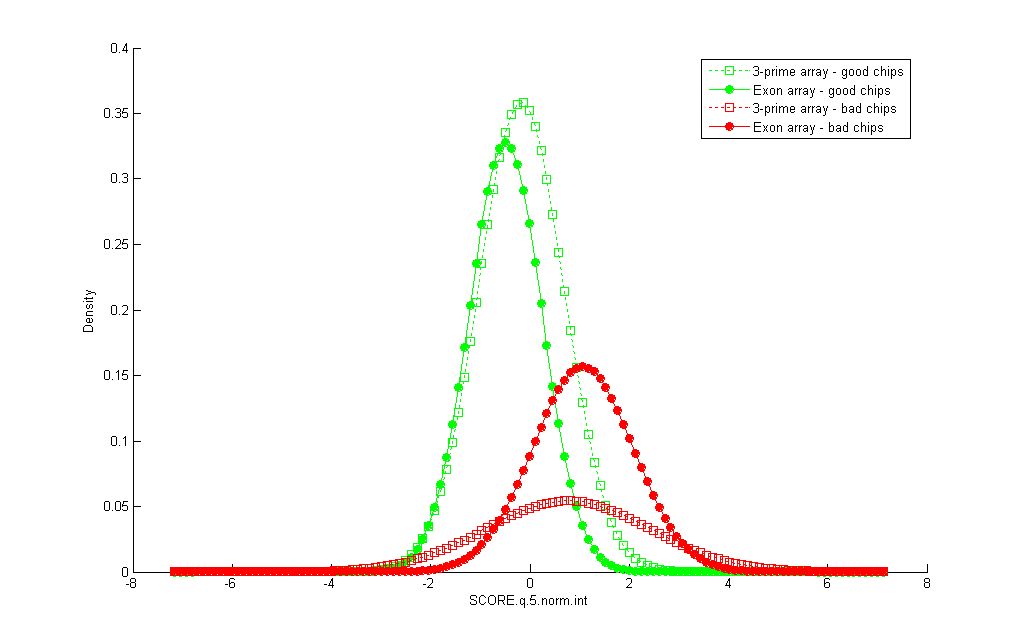

Supplement: Additional file 5 — – SourceCode. Zipped archive contains Matlab source code used for the analyses described in this paper. See the file "READ_ME.txt" for instructions explaining how to run the code. [file 1471-2105-10-191-S5.zip › Output/Distributions - exon versus 3-prime/SCORE.q.5.norm.int_Fig1.tif]

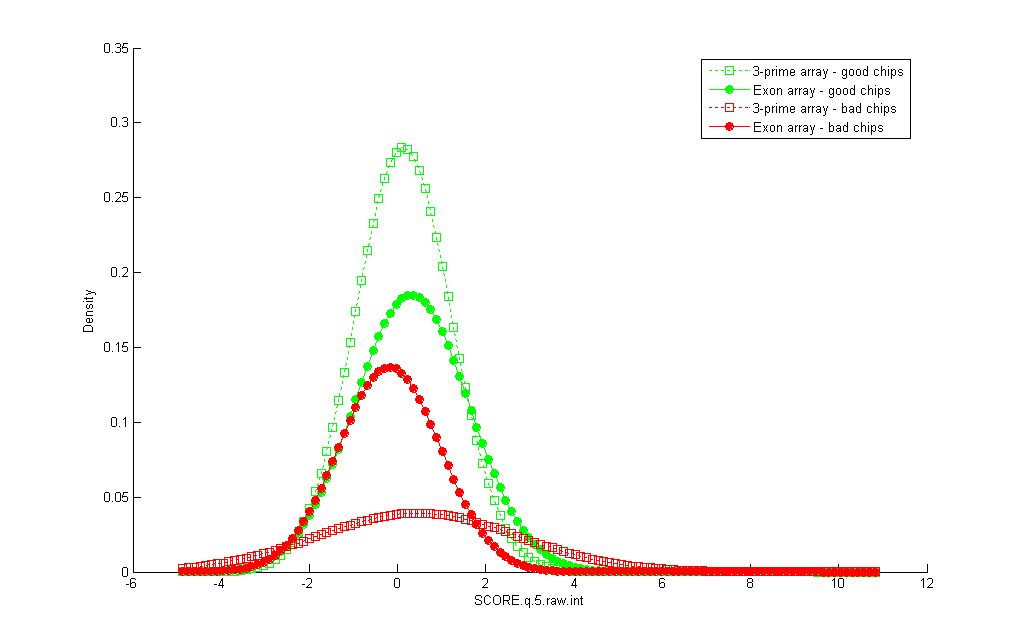

Supplement: Additional file 5 — – SourceCode. Zipped archive contains Matlab source code used for the analyses described in this paper. See the file "READ_ME.txt" for instructions explaining how to run the code. [file 1471-2105-10-191-S5.zip › Output/Distributions - exon versus 3-prime/SCORE.q.5.raw.int_Fig1.tif]

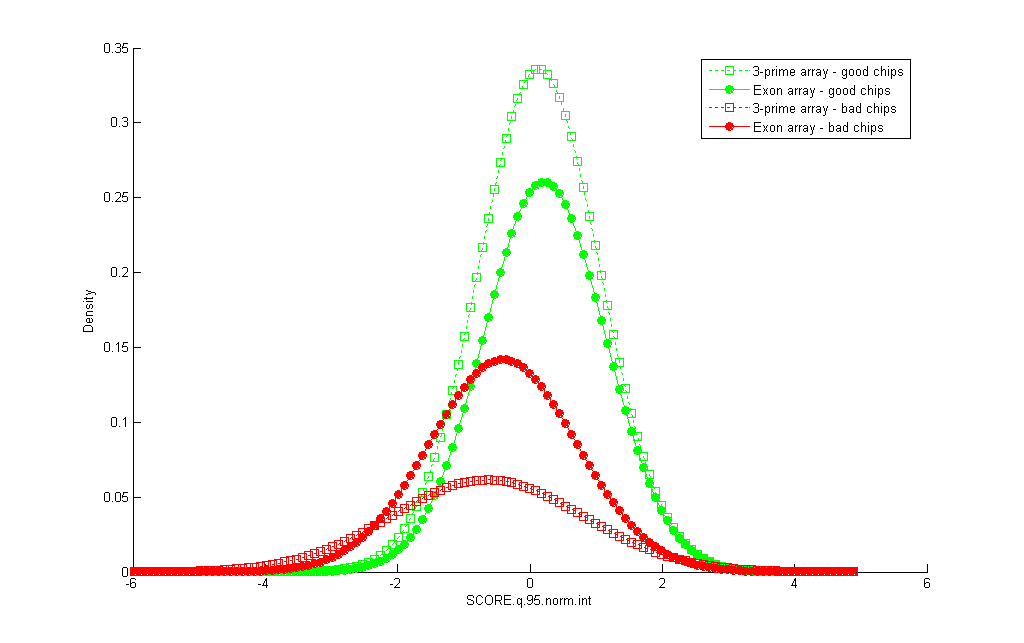

Supplement: Additional file 5 — – SourceCode. Zipped archive contains Matlab source code used for the analyses described in this paper. See the file "READ_ME.txt" for instructions explaining how to run the code. [file 1471-2105-10-191-S5.zip › Output/Distributions - exon versus 3-prime/SCORE.q.95.norm.int_Fig1.tif]

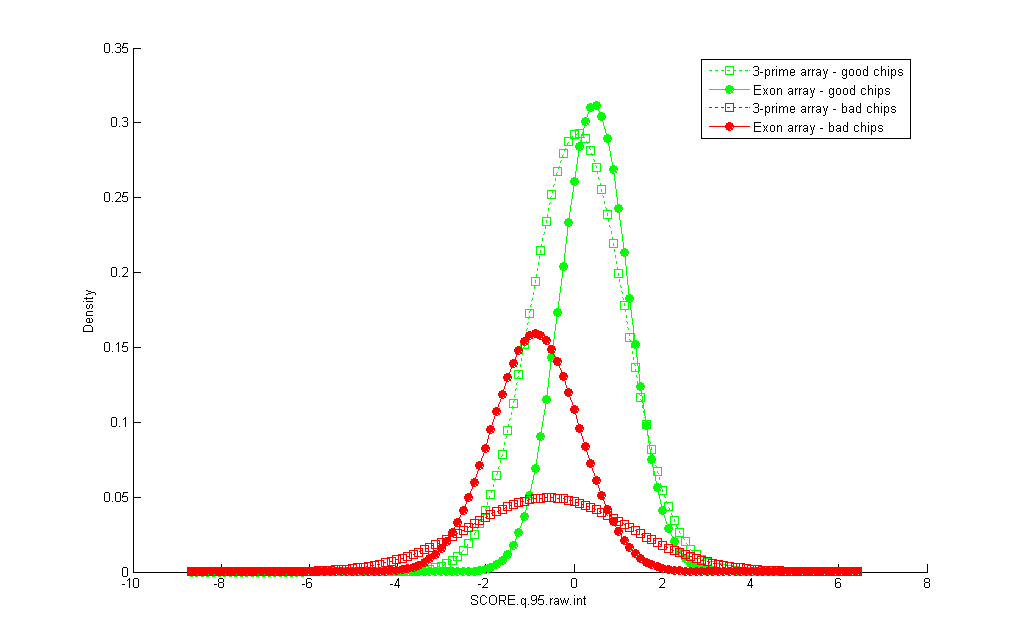

Supplement: Additional file 5 — – SourceCode. Zipped archive contains Matlab source code used for the analyses described in this paper. See the file "READ_ME.txt" for instructions explaining how to run the code. [file 1471-2105-10-191-S5.zip › Output/Distributions - exon versus 3-prime/SCORE.q.95.raw.int_Fig1.tif]

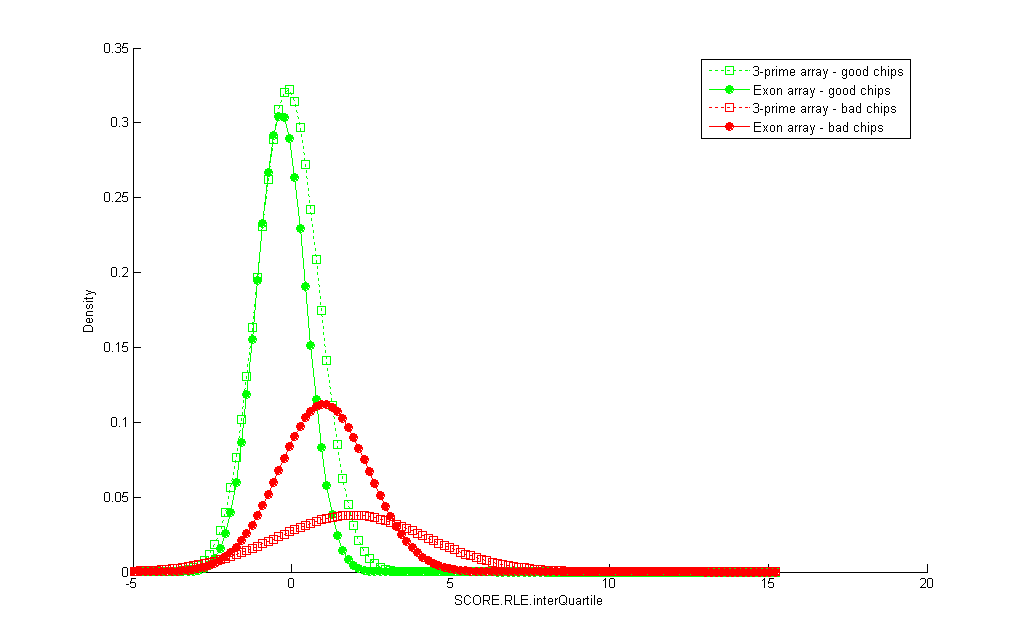

Supplement: Additional file 5 — – SourceCode. Zipped archive contains Matlab source code used for the analyses described in this paper. See the file "READ_ME.txt" for instructions explaining how to run the code. [file 1471-2105-10-191-S5.zip › Output/Distributions - exon versus 3-prime/SCORE.RLE.interQuartile_Fig1.tif]

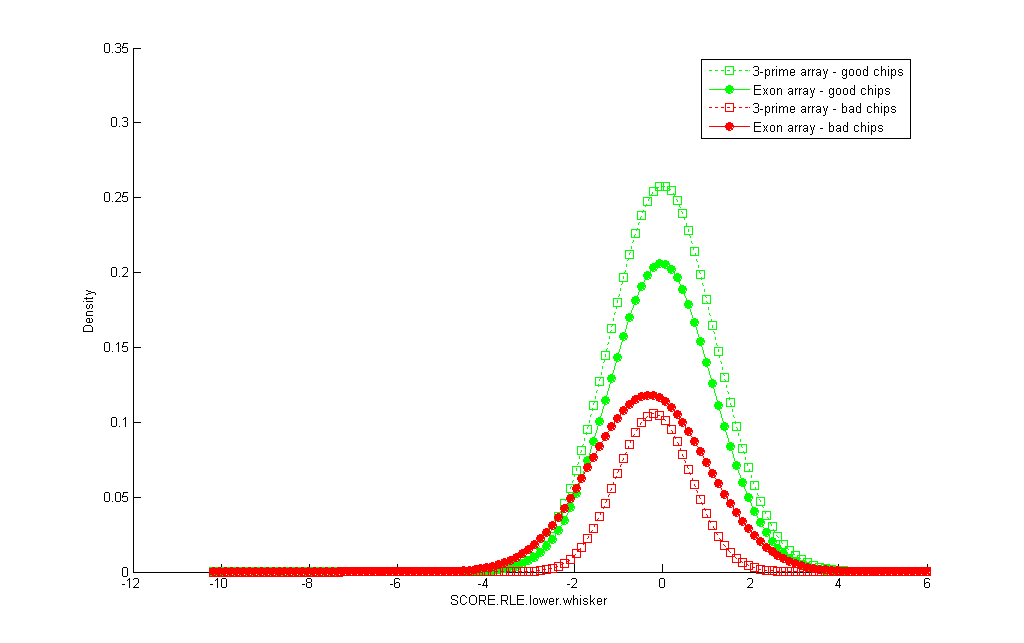

Supplement: Additional file 5 — – SourceCode. Zipped archive contains Matlab source code used for the analyses described in this paper. See the file "READ_ME.txt" for instructions explaining how to run the code. [file 1471-2105-10-191-S5.zip › Output/Distributions - exon versus 3-prime/SCORE.RLE.lower.whisker_Fig1.tif]

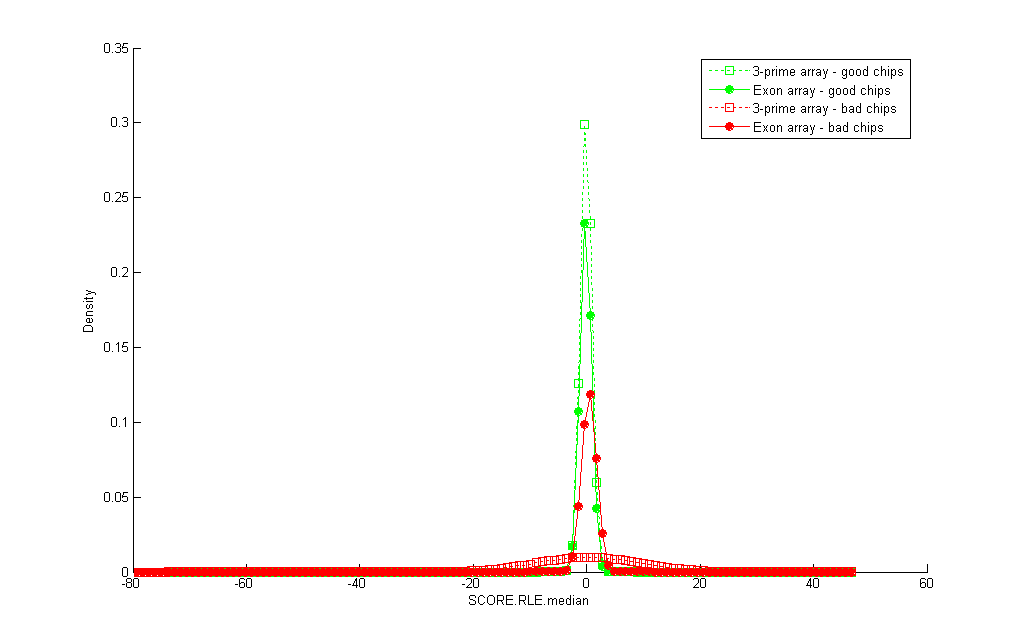

Supplement: Additional file 5 — – SourceCode. Zipped archive contains Matlab source code used for the analyses described in this paper. See the file "READ_ME.txt" for instructions explaining how to run the code. [file 1471-2105-10-191-S5.zip › Output/Distributions - exon versus 3-prime/SCORE.RLE.median_Fig1.tif]

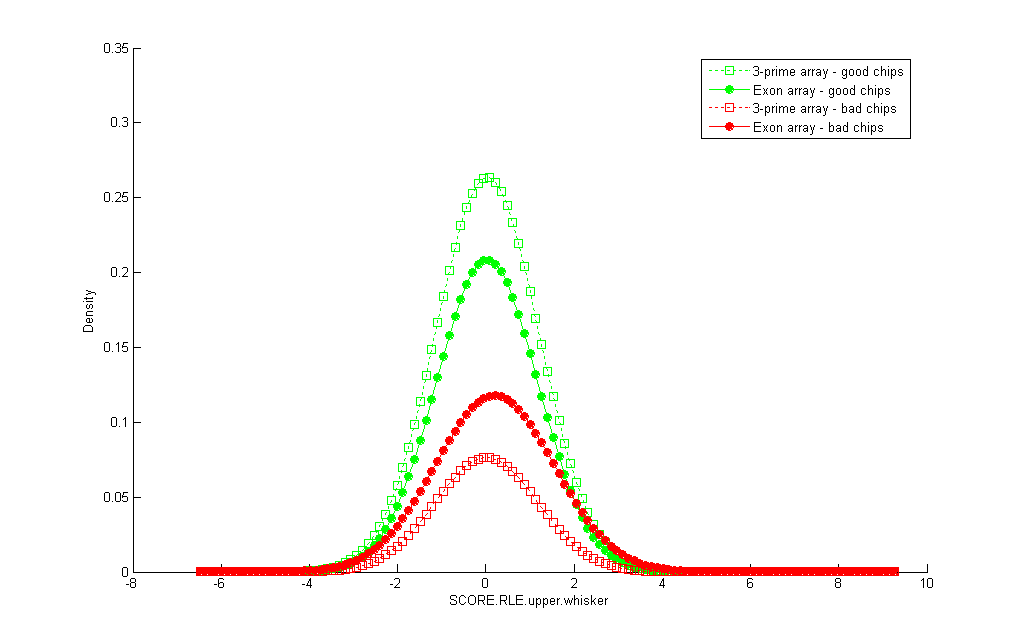

Supplement: Additional file 5 — – SourceCode. Zipped archive contains Matlab source code used for the analyses described in this paper. See the file "READ_ME.txt" for instructions explaining how to run the code. [file 1471-2105-10-191-S5.zip › Output/Distributions - exon versus 3-prime/SCORE.RLE.upper.whisker_Fig1.tif]

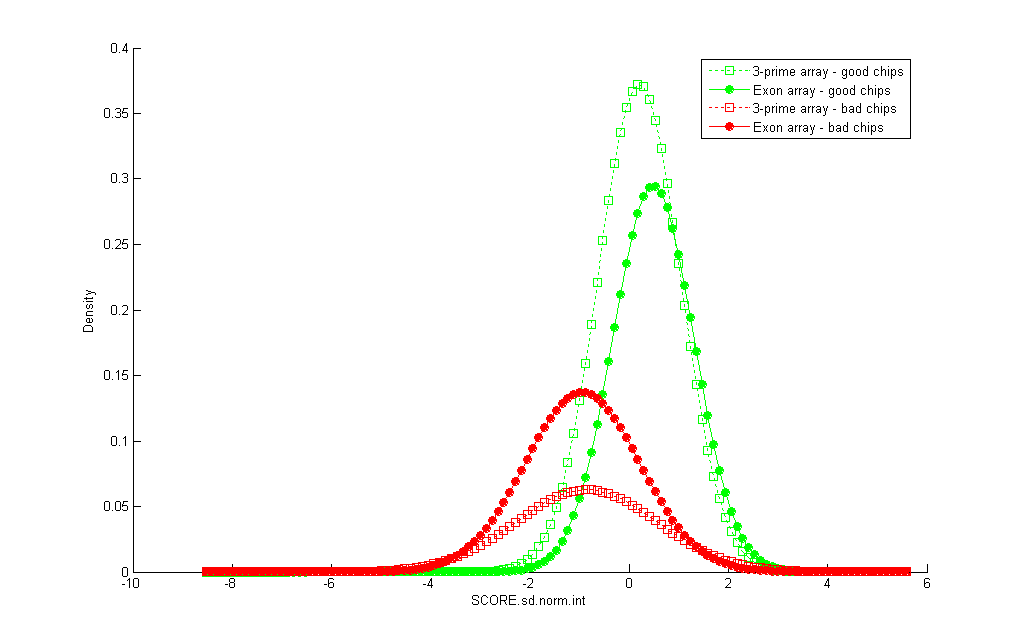

Supplement: Additional file 5 — – SourceCode. Zipped archive contains Matlab source code used for the analyses described in this paper. See the file "READ_ME.txt" for instructions explaining how to run the code. [file 1471-2105-10-191-S5.zip › Output/Distributions - exon versus 3-prime/SCORE.sd.norm.int_Fig1.tif]

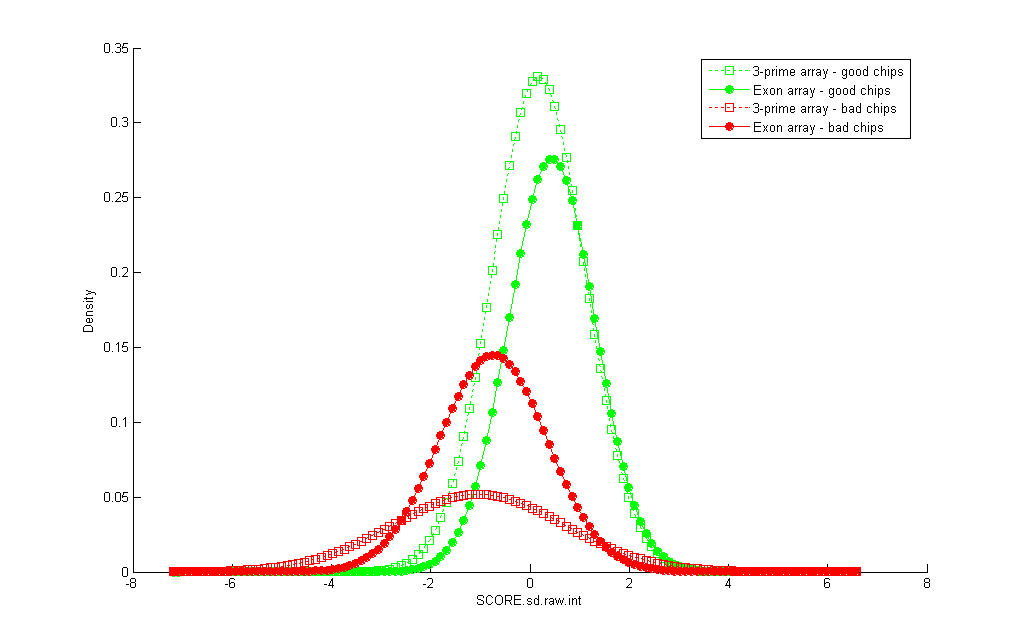

Supplement: Additional file 5 — – SourceCode. Zipped archive contains Matlab source code used for the analyses described in this paper. See the file "READ_ME.txt" for instructions explaining how to run the code. [file 1471-2105-10-191-S5.zip › Output/Distributions - exon versus 3-prime/SCORE.sd.raw.int_Fig1.tif]
